# Synthesis and Pharmacological Evaluation of Enantiopure N-Substituted Ortho-c Oxide-Bridged 5-Phenylmorphans

**DOI:** 10.3390/molecules27248808

**Published:** 2022-12-12

**Authors:** Fuying Li, Theresa A. Kopajtic, Jonathan L. Katz, Dan Luo, Thomas E. Prisinzano, Gregory H. Imler, Jeffrey R. Deschamps, Arthur E. Jacobson, Kenner C. Rice

**Affiliations:** 1 Drug Design and Synthesis Section, Molecular Targets and Medications Discovery Branch, National Institute on Drug Abuse and National Institute on Alcohol Abuse and Alcoholism, National Institutes of Health, Department of Health and Human Services, Rockville, MD 20852, USA; 2 Psychobiology Section, Molecular Neuropsychiatry Research Branch, Intramural Research Program, National Institute on Drug Abuse, National Institutes of Health, Baltimore, MD 21224, USA; 3 Department of Pharmaceutical Sciences, College of Pharmacy, University of Kentucky, Lexington, KY 40536, USA; 4 Center for Biomolecular Science and Engineering, Naval Research Laboratory, Washington, DC 20375, USA

**Keywords:** ortho-c oxide-bridged 5-phenylmorphans, fluorescent Ca2+ mobilization assays, MOR, μ-opioid receptor, DOR, δ-opioid receptor, KOR, κ-opioid receptor, cAMP, cyclic adenosine monophosphate, DAMGO, [D-Ala2; N-Me-Phe4; Gly5-ol]-enkephalin

## Abstract

The design of enantiopure stereoisomers of *N*-2-phenylcyclopropylmethyl-substituted ortho-c oxide-bridged phenylmorphans, the *E* and *Z* isomers of an *N*-cinnamyl moiety, and *N*-propyl enantiomers were based on combining the most potent oxide-bridged phenylmorphan (the ortho-c isomer) with the most potent N-substituent that we previously found with a 5-(3-hydroxy)phenylmorphan (i.e., *N*-2-phenylcyclopropyl methyl moieties, *N*-cinnamyl, and *N*-propyl substituents). The synthesis of the eight enantiopure *N*-2-phenylcyclopropylmethyl ortho-c oxide-bridged phenylmorphans and six additional enantiomers of the N-substituted ortho-c oxide-bridged phenylmorphans (*N*-E and Z-cinnamyl compounds, and *N*-propyl compounds) was accomplished. The synthesis started from common intermediates (3*R*,6a*S*,11a*S*)-10-methoxy-1,3,4,5,6,11a-hexahydro-2*H*-3,6a-methano-benzofuro[2,3-*c*]azocine **(+)-6** and its enantiomer, (3*S*, 6a*R*, 11a*R*)***-*(-)-6**, respectively. The enantiomers of **±-6** were obtained through salt formation with (*S*)-(+)- and (*R*)-(-)-*p*-methylmandelic acid, and the absolute configuration of the (*R*)-(-)-*p*-methylmandelate salt of (3*S*, 6a*R*, 11a*R*)*-***(-)-6** was determined by single-crystal X-ray analysis. The enantiomeric secondary amines were reacted with *N*-(2-phenylcyclopropyl)methyl derivatives, 2-(*E*)-cinnamyl bromide, and (Z)-3-phenylacrylic acid. These products led to all of the desired N-derivatives of the ortho-c oxide-bridged phenylmorphans. Their opioid receptor binding affinity was measured. The compounds with MOR affinity < 50 nM were examined for their functional activity in the forskolin-induced cAMP accumulation assay. Only the enantiomer of the *N*-phenethyl ortho-c oxide-bridged phenylmorphan (**(-)-1**), and only the (3S,6aR,11aR)-2-(((1S,2S)-2-phenylcyclopropyl)methyl)-1,3,4,5,6,11a-hexahydro-2H-3,6a-methanobenzofuro[2,3-c]azocin-10-ol isomer (**(+)-17**), and the N-phenylpropyl derivative (**(-)-25**) had opioid binding affinity < 50 nM. Both **(-)-1** and **(-)-25** were partial agonists in the cAMP assay, with the former showing high potency and low efficacy, and the latter with lower potency and less efficacy. Most interesting was the *N*-2-phenylcyclopropylmethyl (3S,6aR,11aR)-2-(1S,2S)-enantiomer (**(+)-17**). That compound had good MOR binding affinity (Ki = 11.9 nM) and was found to have naltrexone-like potency as a MOR antagonist (IC_50_ = 6.92 nM).

## 1. Introduction

The search for improved analgesics has been ongoing for a century [1,2,3,4]. Those that are now clinically used have side effects that cause concern. These side effects, respiratory depression, gastrointestinal effects, tolerance, dependence, etc., make the use of these analgesics problematic. We have sought a new scaffold that differed from that of the morphine-like compounds, with the hope that it might provide antinociceptives with fewer opioid-like side effects. One of these scaffolds was the ortho-C oxide-bridged phenylmorphan [5,6,7,8,9,10,11,12,13,14]. We have modified the N-substituent with the hope of influencing both opioid receptor affinity (Table 1) and efficacy and potency as determined in the forskolin-induced cAMP accumulation assay (Table 2).

**Figure 1:**
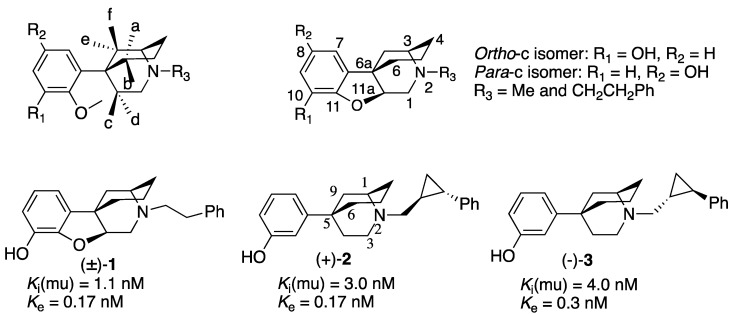
Structures of *N*-substituted ortho- and para-a through f oxide-bridged phenylmorphans and selected ortho-c oxide-bridged phenylmorphan and 5-phenylmorphan ligands.

We have synthesized and pharmacologically evaluated the 12 possible structurally rigid ortho-*a* and para-*a* through -*f* oxide-bridged phenylmorphans in racemic or enantiomeric form [5,6,7,8,9,10,11,12,13,14]. Of all of the a- through f- oxide-bridged phenylmorphans, the racemic *N*-phenethyl ortho-c oxide-bridged phenylmorphan (±-**1**, Figure 1) was found to have the highest mu-opioid receptor affinity (*K*_i_ = 1.1 nM) [14]. The N-substituents in opioids play a major role in affinity and efficacy. We previously investigated the effects of N-substituents on a 5-phenylmorphan scaffold [14,15,16]. The 1*R*,5*S*-*N*-phenylcyclopropylmethyl and 1*S*,5*R*-*N*-phenylcyclopropylmethyl)-5-(3-hydroxyphenyl)morphans were found to have varying affinities at the mu-opioid receptor (MOR) (*K*_i_ = 2–450 nM). Interestingly, compounds acted unusually as inverse agonists in the [^35^S]GTPγS functional assay using nondependent cells that stably express the cloned human mu-opioid receptor [15,16]. Two of the *N*-substituted 5-phenylmorphan compounds with *trans*-2-phenyl-cyclopropylmethyl groups (**(+)-2** and **(-)-3**, Figure 1) showed the highest affinity at MOR (*K*_i_ = 3 and 4 nM, respectively), and possessed very potent mu-opioid antagonist activity (*K*_e_ = 0.17 and 0.3 nM, respectively). We were interested in determining whether the combination of the most potent (ortho-c) oxide-bridged phenylmorphan scaffold and the conformationally restrained phenylcyclopropylmethyl moieties would have modifed potency and efficacy in functional assays. Herein, we report the synthesis of enantiopure *N*-2-phenylcyclopropylmethyl ortho-c oxide-bridged phenylmorphans, their binding affinities at opioid receptors, and their functional activity as agonists or antagonists. The chiral atoms in the ortho-c oxide-bridged phenylmorphans were either 3*S*,6a*R*,11a*R* or a 3*R*,6a*S*,11a*S* (see Figure 1 for atom numbering). For each of those ortho-c oxide-bridged phenylmorphan enantiomers, four *N*-2-phenylcyclopropylmethyl diastereomers needed to be synthesized. These eight stereoisomers and other N-substituted enantiomeric 5-phenylmorphans (*N*-E and Z-cinnamyl compounds, and *N*-propyl compounds) were synthesized. The opioid binding affinity and functional activity of these compounds were compared with the resolved (-)-*N*-phenethyl-ortho-c oxide-bridged 5-phenylmorphan (-)-**1**.

## 2. Results and Discussion

### 2.1. Chemistry

**Scheme 1:**
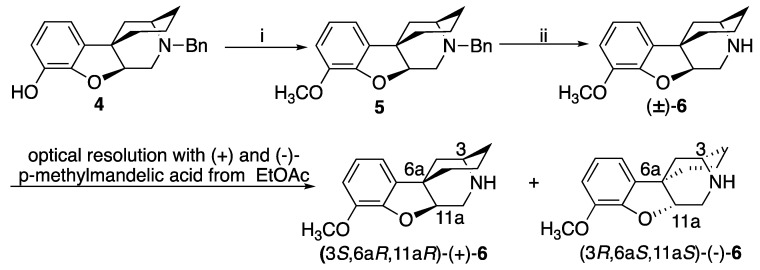
Synthesis and optical resolution of (3R*,6aS*,11aS*)-10-methoxy-1,3,4,5,6,11a-hexahydro-2*H*-3,6a-methanobenzo-furo[2,3-*c*]azocine (±)-**6**. Reagents and conditions: (i) MeI, K_2_CO_3_, DMF, rt, overnight, 87.7%; (ii) H_2_, 5% Pd/C, 50 °C, MeO/AcOH (10:1, *v*/*v*), overnight, 92.9%.

The synthesis started from common intermediates (3*R*,6a*S*,11a*S*)-10-methoxy-1,3,4,5,6,11a-hexahydro-2*H*-3,6a-methano-benzofuro[2,3-*c*]azocine **(+)-6** and its enantiomer, (3*S*, 6a*R*, 11a*R*)*-***(-)-6**, respectively. The racemic **(±)-6** was prepared from the known compound **4** (Scheme 1, see Supplementary Materials) [11], which was synthesized in 11 steps (4.5% overall yield) from commercially available reagents. The methylation of the free phenolic hydroxyl group with MeI gave methyl ether **5** in 88% yield, which was *N*-debenzylated through hydrogenation to afford the desired secondary amine **(±)-6** in 93% yield.

**Figure 2:**
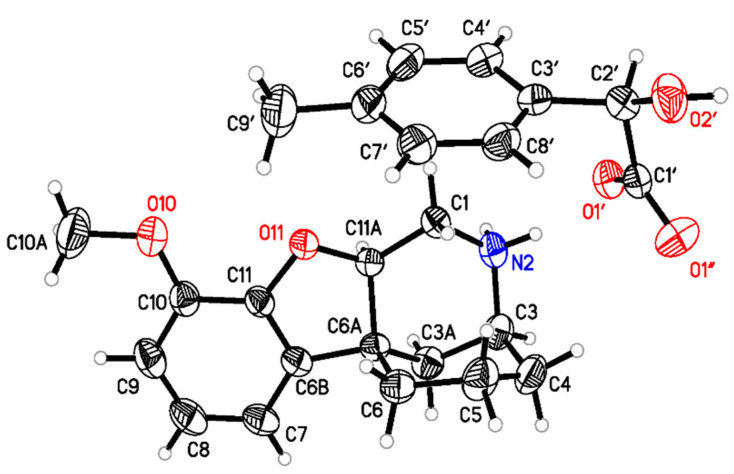
X-ray crystal structure of the (*R*)-(-)-*p*-methylmandelate of (3*S*,6a*R*,11a*R*)-(-)-10-methoxy-1,3,4,5,6,11a-hexahydro-2*H*-3,6a-methano-benzofuro[2,3-*c*]azocine **(-)-6**. The ellipsoids are shown at the 50% probability level. Crystal data and atomic coordinates can be found in the Supplementary Materials.

Both enantiomers of (±)-**6** were resolved via diastereomeric salt formation with (*S*)-(+)- and (*R*)-(-)-*p*-methylmandelic acid (Scheme 1). The absolute configuration of the (*R*)-(-)-*p*-methylmandelate salt of (3*S*, 6a*R*, 11a*R*)*-***(-)-6** was determined by single-crystal X-ray analysis, based on the absolute configuration of the known (*R*)-(-)-*p*-methylmandelic acid used for the preparation of the salt (Figure 2). Different chiral acids were attempted to resolve the racemic amine. **(±)-6**. L-(+)-Tartaric acid, di-p-toluoyl-L-tartaric acid and (1*R*,3*S*)-(+)-camphoric acid failed to resolve the racemate. (*S*)-Mandelic acid and (1*R*)-(-)-camphorsulfonic acid did not afford crystals. Only *p*-bromo- and *p*-methylmandelic acid succeeded in resolving the racemate. The substituent on the phenolic hydroxyl group also affected the optical resolution. The racemic *N*-cyclopropylmethyl (CPM) substituent combined with a phenolic ether failed to form crystals, even with *p*-bromo- and *p*-methylmandelic acid.

**Scheme 2:**
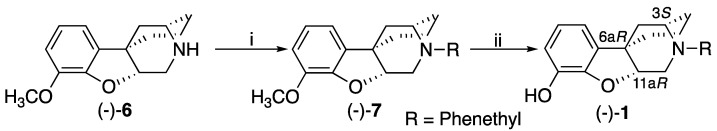
Synthesis of (3*S*,6a*R*,11a*R*)-2-phenethyl-1,3,4,5,6,11a-hexahydro-2*H*-3,6a-methanobenzofuro[2,3-*c*]azocin-10-ol (**(-)-1**). Reagents and conditions: (i): PhCH_2_CH_2_Br, K_2_CO_3_, CH_3_CN, 80 °C, overnight, 84.9%; (ii): BBr_3_, CHCl_3_, −78 °C to rt, 1 h, 87.1%.

Having both enantiomers (**(+)-6** and **(-)-6**) in hand, we focused our attention on the synthesis of their *N*-phenethyl enantiomers. Alkylation with phenethyl bromide afforded (3*S*,6a*R*,11a*R*)-**(-)-**10-methoxy-2-phenethyl-1,3,4,5,6,11a-hexahydro-2*H*-3,6a-methanobenzofuro[2,3-*c*]azocine, **(-)-7** in 85% yield, followed by *O*-demethylation of the aryl methoxy group with BBr_3_ giving the enantiomeric **(-)-1** in 87% yield (Scheme 2).

**Scheme 3:**
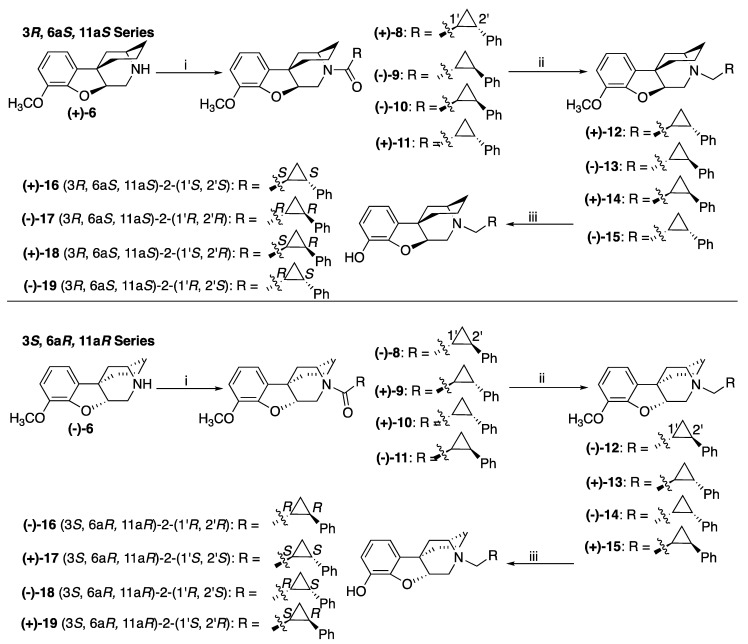
Synthesis of the *N*-(2-phenylcyclopropyl)methyl enantiomers of **(+)-** and **(-)-**1,3,4,5,6,11a-hexahydro-2*H*-3,6a-methano-benzofuro[2,3-*c*]azocin-10-ol (**(+)-** and **(-)-14** through **17**). Reagents and conditions: (i) corresponding enantiomeric carboxylic acid, TBTU, Et_3_N, CHCl_3_, rt, 1 h; (ii) LiAlH_4_, THF, reflux, 3 h; (iii) BBr_3_, CHCl_3_, −78 °C to rt, 1 h.

All of the *N*-(2-phenylcyclopropyl)methyl derivatives (Scheme 3) were prepared using the slightly modified procedures we reported for preparing *N*-substituents [15]. The enantiomeric secondary amines **(+)-** and **(-)-6** were coupled to yield various amides **(+)-** and **(-)-8** through **11** with the corresponding chiral 2-phenylcyclopropane-1-carbolic acids, which were resolved using literature procedures [14]. Reduction of the amides (+)- and **(-)-8** through **11** to the tertiary amines **(+)-** and **(-)-12** through **15** with LiAlH_4_, followed by *O*-demethylation of the aryl methoxy group with BBr_3_ to corresponding free phenols resulted in two sets of analogs, 3*R*,6a*S*,11a*S* series (Scheme 3, top) and 3*S*,6a*R*,11a*R* series (Scheme 3, bottom).

**Scheme 4:**
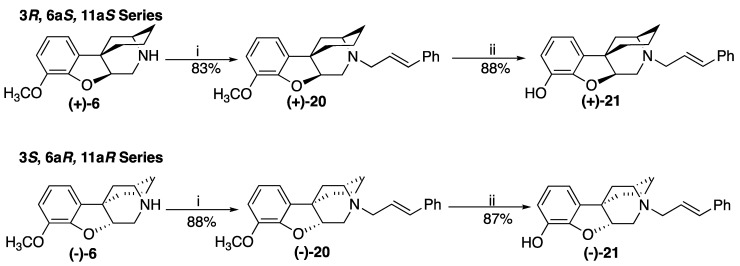
Synthesis of (*E*)-cinnamyl enantiomers of **(+)-** and **(-)-**1,3,4,5,6,11a-hexahydro-2*H*-3,6a-methano-benzofuro[2,3-*c*]azocin-10-ol (**(+)-** and **(-)-21**). Reagents and conditions: (i) 2-(*E*)-cinnamyl bromide, K_2_CO_3_, DMF, rt, overnight; (ii) BBr3, CHCl3, −78 °C to rt, 1 h.

*N-*(*E*)-Cinnamyl enantiomers **(+)-** and **(-)-21** were prepared using a similar procedure with the synthesis of compound **(-)-1** (Scheme 4). The compounds **(+)-** and **(-)-6** were alkylated with (*E*)-cinnamyl bromide afforded **(+)-** and **(-)-20** in 83 and 88% yield, respectively, followed by *O*-demethylation of the aryl methoxy group with BBr_3_ giving the enantiomeric **(+)-** and **(-)-21** in 88% and 87% yield, respectively.

**Scheme 5:**
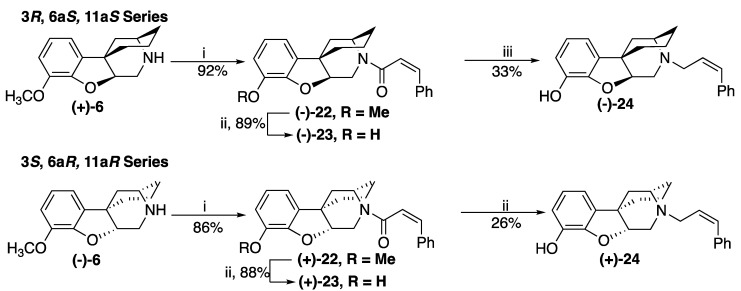
Synthesis of 2-(*Z*)-3-phenylallyl enantiomers of **(+)-** and **(-)-**1,3,4,5,6,11a-hexahydro-2*H*-3,6a-methano-benzofuro[2,3-*c*]azocin-10-ol (**(+)-** and **(-)-24**). Reagents and conditions: (i) (*Z*)-3-phenylacrylic acid, TBTU, CHCl3, rt, 3 h; (ii) BBr_3_, CHCl_3_, −78 °C to rt, 1 h; (iii) LiAlH_4_ or Red-Al, THF, 0 °C to reflux.

*N-*(*Z*)-3-Phenylallyl enantiomers **(+)-** and **(-)-24** (Scheme 5) were prepared through amidation, O-demethylation, and reduction sequence. Coupling of the secondary amines (+) and **(-)-6** with (*Z*)-3-phenylacrylic acid gave the corresponding amides (+) and **(-)-22** in 92% and 86% yields, respectively. The *O*-demethylation of the amides with BBr_3_ went smoothly to afford phenols (+) and **(-)-23** in 89% and 88% yields, respectively, without isomerization observed. The reduction of phenolic amides with LiAlH_4_ yielded the target enantiomers **(+)-** and **(-)-24** in 33% and 25.8% yields, respectively, and the corresponding over-reduced side-products **(+)-** and **(-)-25**. The sequence of demethylation and reduction is important to avoid the isomerization of *Z*-phenylpropene to *E*-phenylpropene.

**Scheme 6:**
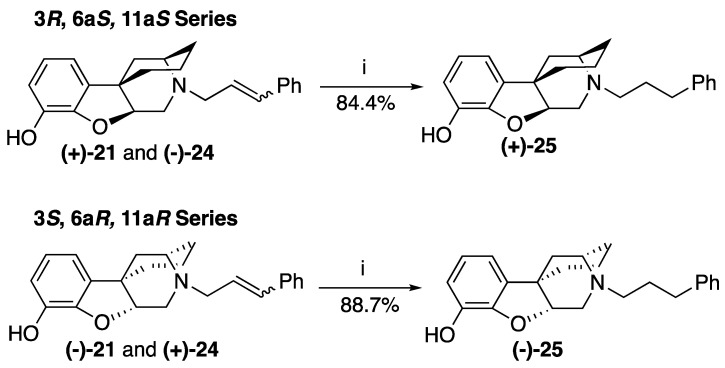
Synthesis of enantiomers of **(+)-** and **(-)-**2-(3-phenylpropyl)-1,3,4,5,6,11a-hexahydro-2*H*-3,6a-methanobenzofuro[2,3-*c*]azocin-10-ol (**(+)-** and **(-)-25**). Reagents and conditions: (i) H_2_ (balloon), 5% Pd/C, MeOH, rt, overnight.

The *N*-phenylpropyl derivatives **(+)-** and **(-)-25** were also synthesized from either *N-*(*E*)-3-phenylallyl analogs **(+)-** and **(-)-24** or *N-*(*Z*)-3-phenylallyl analogs **(+)-** and **(-)-24** through palladium-catalyzed hydrogenation in 84% and 89% yields, respectively (Scheme 6).

### 2.2. In Vitro Studies

#### 2.2.1. Opioid Receptor Binding Data

The binding affinity of the compounds for the opioid receptors, MOR, DOR, and KOR, was determined and only three compounds were found to have MOR affinity < 50 nM (Table 1). The (-)-*N*-phenethyl ortho-c oxide bridged phenylmorphan enantiomer **(-)-1** had a low nanomolar affinity at MOR (*K*i = 1.1 nM), about 10-fold lower KOR affinity, and approximately 30-fold lower affinity at DOR. The (-)-*N*-phenylpropyl enantiomer **(-)-25**, had a moderate affinity at MOR (*K*i = 21 nM), approximately 3-fold lower affinity at DOR, and 6-fold lower affinity at KOR. One of the *N*-cyclopropylmethyl enantiomers, **(+)-17**, had a moderate affinity (*K*i = 11.9 nM) at MOR, and approximately 4- and 10-fold lower affinity at DOR and KOR, respectively.

#### 2.2.2. Ligand Efficacy and Potency (Forskolin-Induced cAMP Accumulation Assay)

The functional activity of these three compounds, **(-)-1**, **(+)-17**, and **(-)-25,** was examined in the forskolin-induced cAMP accumulation assay (Table 2). The most interesting compound was **(+)-17** which was found to be more potent than naltrexone, with an IC_50_ = 6.9 nM at MOR (naltrexone IC_50_ = 10.78) and which, unlike naltrexone, did not have functional agonist activity at KOR or DOR. In accordance with their binding affinity (Table 1), **(-)-1** was a potent (2.66 nM), albeit low efficacy (%Efficacy = 35) partial agonist, and **(-)-25** was less potent (21.62 nM) and had lower efficacy (%Efficacy = 22.5). These low efficacy partial agonists, **(-)-1** or **(-)-25** would not be expected to have much antinociceptive activity.

#### 2.2.3. Pharmacokinetic Assay of MOR Antagonist **(+)-17**

The in vitro data from the forskolin-induced cAMP accumulation assay (Table 2) indicated that **(+)-17** was a little more potent than naltrexone and had a higher degree of antagonism than naloxone. To determine whether its enzymatic effect was similar to or different than naltrexone, a pharmacokinetic study was undertaken.

Compound **(+)-17**, naltrexone, and naloxone were analyzed for their inhibitory effects on the activity of selected human liver microsomal cytochrome P450 isozymes (CYP1A2, CYP2C9, CYP2C19, CYP2D6, and CYP3A) (Table 3). At 10 µM, **(+)-17** showed significant (>50%) inhibitory activity on the 2D6 isozyme. It also displayed inhibitory effects on 1A2, 2C9, 2C19, and 3A to various extents (ranging from 6.8% to 46.6%) indicating this molecule may cause potential drug–drug interactions. Unfortunately, the level of inhibition caused by **(+)-17** was greater than that seen with naltrexone and naloxone. Microsomal metabolic stability of compound **(+)-17**, naltrexone, and naloxone were also evaluated using both human and mouse liver microsomes (Table 4). In human liver microsomes, naltrexone and naloxone showed a much longer half-life than **(+)-17**. A similar pattern was also observed in mouse liver microsome but to a lesser extent (57.4 and 14.4 min vs. 6.9 min). The metabolism of **(+)-17** in the human and mouse microsomes appears to be mostly through NADPH-dependent mechanisms.

## 3. Materials and Methods

### 3.1. General Information

TLC analyses were carried out on Analtech silica gel GHLF 0.25 mm plates with UV and I_2_ detection. Melting points were determined in open glass capillaries on a Thomas Hoover melting point apparatus or MP70 melting point system (manufactured by Mettler Toledo) and were uncorrected. Elemental analyses (C, H, N) were performed by Micro-Analysis, Inc, Wilmington, DE, and were within ±0.4% of the theoretical values. ^1^H NMR and ^13^C NMR spectra were recorded on a Bruker DMX wide-bore spectrometer in CDCl_3_ (unless otherwise noted) at 400 or 500 MHz and 100 or 125 MHz, respectively, with the values given in ppm and J (Hz) assignments of ^1^H resonance coupling. For ^1^H NMR spectra (CDCl_3_), the residual solvent peak was used as the reference (7.26 ppm) while the central solvent peak was used as the ^13^C NMR reference (77.0 ppm in CDCl_3_). The high-resolution electrospray ionization (ESI) mass spectra were obtained on a Waters LCT Premier time-off light (TOF) mass spectrometer. Flash column chromatography was performed with Bodman silica gel LC 60 A. The chiral HPLC was performed on an Agilent 1100 series analytical instrument equipped with UV detector G1315-DAD using (*R*, *R*)-WHELK-O1 column (manufactured by Regis Technologies Int.), 250 × 4.6 mm. The samples for HPLC analyses were dissolved in CH_2_Cl_2_. A mixture of hexane, CH_2_Cl_2_ and 2-propanol (80/15/5), and 0.1% v/v TFA was used as eluent and the flow rate was 1.5 mL/min. The optical rotation was measured with a PerkinElmer 341 polarimeter.

### 3.2. Syntheses

(3R*,6aS*,11aS*)-2-Benzyl-10-methoxy-1,3,4,5,6,11a-hexahydro-2H-3,6a-methano-benzofuro[2,3-c]azocine **((±)-5).** To a stirred suspension of compound (**±**)-**4** (3.72 g, 11.6 mmol) and K_2_CO_3_ (3.2 g, 23.2 mmol) in DMF (200 mL) was added a solution of MeI (1.81 g, 0.79 mL, 12.8 mmol) dropwise and the mixture was stirred at rt overnight. The solvent was removed under reduced pressure and the residue was treated with H_2_O. The mixture was extracted with CH_2_Cl_2_ (3 × 30 mL) and the combined extracts were washed with brine and dried over anhydrous Na_2_SO_4_. After filtration and evaporation, the crude product was purified by flash chromatography (0–10% EtOAc in hexane) to give racemic (**±**)-**5** (3.41 g, 87.7%) as white solid. ^1^H NMR (400 MHz, CDCl_3_): δ 7.34 (d, *J* = 6.8 Hz, 2H), 7.29 (t, *J* = 6.8 Hz, 2H), 7.22 (d, *J* = 8.0 Hz, 1H), 6.85 (t, *J* = 7.6 Hz, 1H), 6.72 (t, *J* = 7.6 Hz, 2H), 4.30 (m, 1H), 3.92 (d, *J* = 13.6 Hz, 1H), 3.84 (s, 3H), 3.80 (d, *J* = 14.0 Hz, 1H), 3.42 (t, *J* = 11.2 Hz, 1H), 3.29 (m, 1H), 3.10 (s, 1H), 2.24 (d, *J* = 12.8 Hz, 1H), 2.08 (d, *J* = 12.0 Hz, 1H), 1.99 (t, *J* = 12.0 Hz, 1H), 1.82 (m, 2H), 1.63 (m, 1H), 1.43 (m, 2H); ^13^C NMR (100 MHz, CDCl_3_) δ 147.4, 145.1, 139.9, 139.5, 128.4 (2), 128.2 (2), 126.9, 121.8, 114.1, 111.2, 89.3, 58.6, 55.9, 52.4, 51.0, 44.5, 36.7, 31.9, 26.6, 21.8; ESI-MS 336.2 (M^+^ + H); HRMS (ES^+^) calcd for C_22_H_26_NO_2_ (M^+^ + H) 336.1958; found 336.1958.

(3R*,6aS*,11aS*)-10-Methoxy-1,3,4,5,6,11a-hexahydro-2H-3,6a-methanobenzofuro-[2,3-c]azocine **((±)-6).** A flask charged with compound (**±**)-**5** (3.82 g, 11.4 mmol), 10% Pd/C (0.8 g), AcOH (10 mL), and MeOH (100 mL) was evacuated and backfilled with H_2_ three times. The mixture was hydrogenated under H_2_ (50 psi) at 50 °C overnight. The mixture was filtered, and the filtrate was concentrated. The residue was basified with 28% NH_4_OH and extracted with CH_2_Cl_2_ (3 × 30 mL) and the combined extracts were washed with brine and dried over anhydrous Na_2_SO_4_. After filtration and evaporation, the crude product was purified by flash chromatography (CHCl_3_:MeOH:NH_4_OH = 90:9:1) to give racemic (**±**)-**6** (2.6 g, 92.9%) as clear oil. ^1^H NMR (400 MHz, CDCl_3_): δ 6.85 (t, *J* = 7.6 Hz, 1H), 6.74 (d, *J* = 8.0 Hz, 1H), 6.69 (d, *J* = 7.2 Hz, 1H), 4.10 (dd, *J* = 12.0, 5.2 Hz, 1H), 3.85 (s, 3H), 3.71 (t, *J* = 12.0 Hz, 1H), 3.35 (dd, *J* = 12.0, 5.6 Hz, 1H), 3.27 (s, 1H), 2.16 (d, *J* = 12.0 Hz, 1H), 2.02 (m, 1H), 1.86 (m, 2H), 1.81 (m 2H), 1.67 (m, 2H), 1.44 (m, 1H); ^13^C NMR (100 MHz, CDCl_3_) δ 146.7, 145.1, 139.9, 121.8, 114.0, 111.1, 90.3, 55.9, 47.7, 45.8, 44.6, 38.8, 33.2, 32.0, 21.7; ESI-MS 246.1 (M^+^ + H); HRMS (ES^+^) calcd for C_15_H_20_NO_2_ (M^+^ + H) 246.1489; found 246.1489.

Optical resolution of (3R*,6aS*,11aS*)-10-Methoxy-1,3,4,5,6,11a-hexahydro-2H-3,6a-methano-benzofuro[2,3-c]azocine **((±)-6)**. To a solution of the racemate (±)-**6** (3.3 g, 13.4 mmol) in acetone (30 mL) was added (*S*)-(+)-*p*-methylmandelic acid (2.24 g, 13.5 mmol), and a clear solution was obtained. The solvent was evaporated under reduced pressure and the salt was treated with EtOAc (40 mL). The solution was heated up to reflux and the solvent was distilled with a Dean–Stark trap until around 20 mL of EtOAc was distilled off and a white solid appeared. The solution was cooled to room temperature overnight. A foam solid was collected (2.1 g, 37.9%). The salt was recrystallized from EtOAc (40 mL) to yield a white solid (1.61 g, 28.9%). A small portion was free-based and analyzed by chiral HPLC (ee > 99%, retention time 12.56 min): [α]^20^_D_ = + 79.8° (CHCl_3_, c 1.04), mp 106.4–109.5 °C. The initial filtrate and mother liquors were recovered, evaporated, and free-based to give **(-)-6**-enriched free-base (2.34 g, 70.9%), which was dissolved in acetone (30 mL) and (*R*)-(-)-p-methylmandelic acid (1.59 g, 9.6 mmol) was added in one portion. The solution was concentrated, and the salt was crystallized from EtOAc (40 mL and 60 mL) twice to yield a white foam (2.6 g, 46.9%). A small portion was free-based and analyzed by chiral HPLC (ee > 99%, retention time 8.53 min): [α]^20^_D_ = −77.6° (CHCl_3_, c 1.02), mp 106.4–108.8 °C. The absolute stereochemistry of (3*S*, 6a*R*, 11a*R*)*-***(-)-6** was established by single crystal X-ray analysis of the (*R*)-(-)-*p*-methylmandelate salt.

(3S,6aR,11aR)-10-Methoxy-2-phenethyl-1,3,4,5,6,11a-hexahydro-2H-3,6a-methano-benzofuro[2,3-c]-azocine **((-)-7).** A mixture of **(-)-6** (60 mg, 0.24 mmol), K_2_CO_3_ (101 mg, 0.73 mmol), phenethyl bromide (89 mg, 66 μL, 0.48 mmol), and CH_3_CN (5 mL) was heated at 80 °C overnight. The mixture was filtered, and the filtrate was concentrated. The crude product was purified by flash chromatography (10–30% EtOAc in hexane) to give **(-)-7** (73 mg, 84.9%) as a clear oil. ^1^H NMR (400 MHz, CDCl_3_): δ 7.28 (t, *J* = 7.2 Hz, 2H), 7.22 (m, 3H), 6.88 (t, *J* = 7.6 Hz, 1H), 6.77 (d, *J* = 8.0 Hz, 1H), 6.73 (d, *J* = 6.8 Hz, 1H), 4.10 (dd, *J* = 11.2, 5.6 Hz, 1H), 3.89 (s, 3H), 3.43 (m, 2H), 3.20 (s, 1H), 2.93 (m, 2H), 2.84 (m, 2H), 2.20 (d, *J* = 13.2 Hz, 1H), 2.14 (d, *J* = 12.0 Hz, 1H), 1.99 (d, *J* = 12.0 Hz, 1H), 1.79 (m, 2H), 1.62 (m, 1H), 1.46 (m, 2H); ^13^C NMR (100 MHz, CDCl_3_) δ 147.5, 145.2, 140.5, 139.8, 128.8 (2), 128.4 (2), 126.1, 122.0, 114.2, 111.1, 89.2, 56.2, 56.0, 53.0, 51.3, 44.6, 36.9, 35.1, 31.9, 26.7, 21.9; [α]^20^_D_ = −72.7° (CHCl_3_, c 1.05); ESI-MS 350.2 (M^+^ + H); HRMS (ES^+^) calcd for C_23_H_28_NO_2_ (M^+^ + H) 350.2115; found 350.2113.

(3S,6aR,11aR)-2-Phenethyl-1,3,4,5,6,11a-hexahydro-2H-3,6a-methano-benzofuro[2,3-c]azocin-10-ol **((-)-1)**. To a solution of BBr_3_ (0.26 g, 0.1 mL, 1.04 mmol) in CHCl_3_ (10 mL) at −78 °C under N_2_ was added a solution of **(-)-7** (73 mg, 0.21 mmol) and the resulting solution was warmed to rt gradually and stirred for 1 h at rt. The solution was cooled to −78 °C and the reaction was quenched with 28% NH_4_OH. The mixture was extracted with CHCl_3_ (3 × 10 mL) and the combined extracts were washed with brine and dried over anhydrous Na_2_SO_4_. After filtration and evaporation, the crude product was purified by flash chromatography (25% EtOAc in hexane) to yield **(-)-1** (63 mg, 87.1%) as a clear oil. ^1^H NMR (400 MHz, CDCl_3_): δ 7.27 (m, 2H), 7.20 (m, 3H), 6.78 (t, *J* = 7.2 Hz, 1H), 6.73 (d, *J* = 7.2 Hz, 1H), 6.62 (d, *J* = 6.0 Hz, 1H), 4.28 (m, 1H), 3.54 (m, 1H), 3.37 (t, *J* = 10.8 Hz, 1H), 3.28 (s, 1H), 2.92 (m, 4H), 2.25 (d, *J* = 12.8 Hz, 1H), 2.12 (d, *J* = 12.0 Hz, 1H), 2.01 (d, *J* = 12.0 Hz, 1H), 1.78 (m, 2H), 1.63 (m, 1H), 1.48 (m, 2H); ^13^C NMR (100 MHz, CDCl_3_) δ 146.5, 141.5, 140.3, 139.6, 128.9 (2), 128.5 (2), 126.2, 122.3, 116.2, 113.6, 89.0, 56.6, 52.6, 51.5, 44.4, 36.7, 34.6, 31.7, 26.3, 21.8; [α]^20^_D_ = −46.2° (CHCl_3_, c 1.0); ESI-MS 336.2 (M^+^ + H); HRMS (ES^+^) calcd for C_22_H_26_NO_2_ (M^+^ + H) 336.1958; found 336.1956; Anal. Calcd for C_22_H_25_NO_2_•HCl•0.5H_2_O: C 69.37, H 7.14, N 3.68; found C 69.33, H 7.18, N 3.72; Mp 259.0–261.7 °C (HCl salt).

*General procedure for synthesis of amides* (**(+)-** *and* **(-)-8** *through* **11**). To a solution of **(+)-** or **(-)-**6 (92 mg, 0.38 mmol, 1 eq.) in CHCl_3_ (10 mL) was added the corresponding carbolic acids (91 mg, 0.57 mmol, 1.5 eq.), Et_3_N (0.16 mL, 1.14 mmol, 3 eq.) and the coupling reagent *O*-(benzotriazol-1-yl)-*N,N,N′,N*′-tetramethyluronium tetrafluoroborate (TBTU, 244 mg, 0.76 mmol, 2 eq) and the resulting solution was stirred at rt for 1 h. The solution was diluted with CHCl_3_ (20 mL) and washed with aqueous HCl solution (2 M, 10 mL), water (10 mL), saturated NaHCO_3_ and brine, successively, and then dried over anhydrous Na_2_SO_4_. After filtration and evaporation, the crude product was purified by flash chromatography (10–30% EtOAc in hexane) to afford the corresponding amides.

((3R,6aS,11aS)-10-Methoxy-1,3,4,5,6,11a-hexahydro-2H-3,6a-methanobenzofuro[2,3-c]azocin-2-yl)((1S,2S)-2-phenylcyclopropyl)methanone (**(+)-8**): clear oil; yield 85.6%; ^1^H NMR (400 MHz, CDCl_3_): δ 7.29 (m, 2H), 7.21 (m, 1H), 7.13 (m, 2H), 6.90 (m, 1H), 6.79 (d, J = 8.0 Hz, 1H), 6.73 (d, J = 7.2 Hz, 1H), 5.01 (m, 1H), 4.60 (m, 1H), 4.10 (m, 1H), 3.88 (s, 3H), 3.66 (m, 1H), 2.56 (m, 1H), 2.18 (m, 1H), 2.00 (m, 3H), 1.88 (m, 2H), 1.67 (m, 3H), 1.52 (m, 1H), 1.30 (m, 1H); ^13^C NMR (100 MHz, CDCl_3_) δ 172.6, 172.2, 147.2, 147.0, 145.2, 145.1, 140.89, 140.86, 138.5, 138.3, 128.6, 128.5, 126.37, 126.35, 126.1, 126.0, 122.5, 122.3, 114.2, 114.1, 111.6, 111.5, 87.7, 87.6, 56.0, 50.0, 47.2, 47.0, 44.8, 44.7, 44.4, 36.3, 35.5, 32.3, 31.4, 31.3, 30.2, 25.6, 25.4, 24.4, 24.1, 21.1, 21.0, 16.9, 16.6; [α]^20^_D_ = +116.5° (CHCl_3_, c 0.95); ESI-MS 390.2 (M^+^ + H); HRMS (ES^+^) calcd for C_25_H_28_NO_3_ (M^+^ + H) 390.2064; found 390.2067.

((3S,6aR,11aR)-10-Methoxy-1,3,4,5,6,11a-hexahydro-2H-3,6a-methanobenzofuro[2,3-c]azocin-2-yl)((1R,2R)-2-phenylcyclopropyl)methanone **((-)-8)**: clear oil; yield 88.9%; ^1^H NMR (400 MHz, CDCl_3_): δ 7.28 (m, 2H), 7.21 (m, 1H), 7.14 (m, 2H), 6.90 (m, 1H), 6.80 (d, J = 8.0 Hz, 1H), 6.74 (d, J = 7.2 Hz, 1H), 5.02 (m, 1H), 4.60 (m, 1H), 4.10 (m, 1H), 3.89 (s, 3H), 3.67 (m, 1H), 2.56 (m, 1H), 2.19 (m, 1H), 2.03 (m, 3H), 1.88 (m, 2H), 1.68 (m, 3H), 1.52 (m, 1H), 1.32 (m, 1H); ^13^C NMR (100 MHz, CDCl_3_) δ 172.6, 172.2, 147.3, 147.1, 145.23, 145.16, 140.92, 140.89, 138.5, 138.3, 128.6, 128.5, 126.4, 126.37, 126.2, 126.0, 122.5, 122.3, 114.2, 114.1, 111.6, 111.5, 87.7, 87.6, 56.0, 50.0, 47.2, 47.0, 44.8, 44.7, 44.4, 36.3, 35.5, 32.4, 31.4, 31.3, 30.2, 25.6, 25.4, 24.4, 24.1, 21.1, 21.0, 16.9, 16.6; [α]^20^_D_ = −117.7° (CHCl_3_, c 0.65); ESI-MS 390.2 (M^+^ + H); HRMS (ES^+^) calcd for C_25_H_28_NO_3_ (M^+^ + H) 390.2064; found 390.2064.

((3S,6aR,11aR)-10-Methoxy-1,3,4,5,6,11a-hexahydro-2H-3,6a-methanobenzofuro[2,3-c]azocin-2-yl)((1S,2S)-2-phenylcyclopropyl)methanone **((+)-9)**: light yellow oil; yield 82.0%; ^1^H NMR (400 MHz, CDCl_3_): δ 7.28 (m, 2H), 7.20 (m, 1H), 7.11 (m, 2H), 6.90 (t, J = 7.2 Hz, 1H), 6.78 (t, J = 7.2 Hz, 1H), 6.74 (m, 1H), 5.01 (m, 1H), 4.56 (m, 1H), 4.08 (m, 1H), 3.89 and 3.86 (s, 3H), 3.69 (m, 1H), 2.46 (m, 1H), 2.19 (m, 1H), 2.03 (m, 2H), 1.83 (m, 5H), 1.66 (m, 1H), 1.53 (m, 1H), 1.28 (m, 1H); ^13^C NMR (100 MHz, CDCl_3_) δ 172.6, 172.3, 147.3, 147.1, 145.2, 145.1, 140.8, 140.7, 138.45, 138.37, 128.65, 128.58, 126.5, 126.4, 126.2, 125.9, 122.5, 122.3, 114.2, 114.1, 111.6, 111.5, 87.8, 87.6, 56.03, 55.97, 50.0, 47.4, 47.0, 44.8, 44.4, 36.3, 35.5, 32.5, 31.4, 31.3, 30.6, 26.23, 26.21, 24.3, 24.0, 21.3, 21.0, 16.7, 16.0; [α]^20^_D_ = +100.4° (CHCl_3_, c 1.46); ESI-MS 390.2 (M^+^ + H); HRMS (ES^+^) calcd for C_25_H_28_NO_3_ (M^+^ + H) 390.2064; found 390.2065.

((3R,6aS,11aS)-10-Methoxy-1,3,4,5,6,11a-hexahydro-2H-3,6a-methanobenzofuro[2,3-c]azocin-2-yl)((1R,2R)-2-phenylcyclopropyl)methanone **((-)-9)**: clear oil; yield 90.5%; ^1^H NMR (400 MHz, CDCl_3_): δ 7.28 (m, 2H), 7.21 (m, 1H), 7.11 (m, 2H), 6.91 (t, J = 7.2 Hz, 1H), 6.79 (t, J = 7.2 Hz, 1H), 6.74 (m, 1H), 5.02 (m, 1H), 4.56 (m, 1H), 4.11 (m, 1H), 3.90 and 3.87 (s, 3H), 3.69 (m, 1H), 2.46 (m, 1H), 2.20 (m, 1H), 2.04 (m, 2H), 1.83 (m, 5H), 1.66 (m, 1H), 1.53 (m, 1H), 1.28 (m, 1H); ^13^C NMR (100 MHz, CDCl_3_) δ 172.7, 172.4, 147.4, 147.1, 145.3, 145.2, 140.9, 140.8, 138.5, 138.4, 128.7, 128.6, 126.5, 126.4, 126.3, 160.0, 122.6, 122.4, 114.3, 114.2, 111.6, 111.4, 87.8, 87.6, 56.1, 56.0, 50.0, 47.4, 47.0, 44.9, 44.5, 36.3, 35.5, 32.6, 31.5, 31.4, 30.6, 26.3, 24.4, 24.1, 21.4, 21.1, 16.8, 16.0; [α]^20^_D_ = −99.1° (CHCl_3_, c 1.0); ESI-MS 390.2 (M^+^ + H); HRMS (ES^+^) calcd for C_25_H_28_NO_3_ (M^+^ + H) 390.2069; found 390.2066.

((3S,6aR,11aR)-10-Methoxy-1,3,4,5,6,11a-hexahydro-2H-3,6a-methanobenzofuro[2,3-c]azocin-2-yl)((1R,2S)-2-phenylcyclopropyl)methanone **((+)-10):** clear oil; yield 88.5%; ^1^H NMR (400 MHz, CDCl_3_): δ 7.13 (m, 5H), 6.86 (m, 1H), 6.75 (m, 1H), 6.64 (m, 1H), 4.75 (m 1H), 4.58 (m, 1H), 4.08 (m, 1H), 3.86 (m, 3H), 3.44 (m, 1H) 2.46 (m, 1H), 2.20 (m, 1H), 2.00 (m, 1H), 1.85 (m, 1H), 1.73 (m, 2H), 1.50 (m, 1H), 1.30 (m, 3H), 1.11 (m, 1H), 0.50 (m, 1H); ^13^C NMR (100 MHz, CDCl_3_) δ 169.5, 168.8, 147.1, 147.0, 145.1, 138.6, 138.4, 137.24, 137.19, 128.2, 128.1, 127.4, 127.1, 126.5, 126.4, 122.4, 122.1, 114.2, 114.0, 111.5, 111.3, 87.4, 87.3, 56.0, 55.9, 49.2, 47.0, 46.7, 44.8, 44.2, 43.6, 36.3, 35.4, 31.9, 31.3, 31.2, 29.8, 26.3, 25.2, 24.0, 23.4, 21.0, 20.1, 10.8, 10.2; [α]^20^_D_ = +130.0° (CHCl_3_, c 1.04); ESI-MS 390.2 (M^+^ + H); HRMS (ES^+^) calcd for C_25_H_28_NO_3_ (M^+^ + H) 390.2064; found 390.2066.

((3R,6aS,11aS)-10-Methoxy-1,3,4,5,6,11a-hexahydro-2H-3,6a-methanobenzofuro[2,3-c]azocin-2-yl)((1S,2R)-2-phenylcyclopropyl)methanone **((-)-10)**: clear oil; yield 89.2%; ^1^H NMR (400 MHz, CDCl_3_): δ 7.15 (m, 5H), 6.85 (m, 1H), 6.75 (m, 1H), 6.64 (m, 1H), 4.75 (m 1H), 4.58 (m, 1H), 4.08 (m, 1H), 3.86 (m, 3H), 3.44 (m, 1H) 2.46 (m, 1H), 2.20 (m, 1H), 2.00 (m, 1H), 1.85 (m, 1H), 1.73 (m, 2H), 1.50 (m, 1H), 1.34 (m, 1H), 1.23 (m, 2H), 1.12 (m, 1H), 0.50 (m, 1H); ^13^C NMR (100 MHz, CDCl_3_) δ 169.5, 168.8, 147.1, 147.0, 145.1, 138.5, 138.4, 137.23, 137.17, 128.2, 128.1, 127.4, 127.1, 126.5, 126.4, 122.4, 122.1, 114.2, 114.0, 111.5, 111.3, 87.4, 87.3, 56.0, 55.9, 49.2, 47.0, 46.7, 44.8, 44.2, 43.6, 36.3, 35.4, 31.9, 31.3, 31.2, 29.8, 26.3, 25.2, 24.0, 23.4, 21.0, 20.1, 10.8, 10.2; [α]^20^_D_ = −131.0° (CHCl_3_, c 0.76); ESI-MS 390.2 (M^+^ + H); HRMS (ES^+^) calcd for C_25_H_28_NO_3_ (M^+^ + H) 390.2064; found 390.2064.

((3R,6aS,11aS)-10-Methoxy-1,3,4,5,6,11a-hexahydro-2H-3,6a-methanobenzofuro[2,3-c]azocin-2-yl)((1R,2S)-2-phenylcyclopropyl)methanone **((+)-11)**: clear oil; yield 82.0%; ^1^H NMR (400 MHz, CDCl_3_): δ ^1^H NMR (400 MHz, CDCl_3_): δ 7.14 (m, 5H), 6.87 (m, 1H), 6.76 (m, 1H), 6.64 (m, 1H), 4.80 (m, 1H), 4.61 (m, 1H), 3.96 (m, 1H), 3.90 (m, 3H), 3.44 (m, 1H), 2.50 (m, 1H), 2.15 (m, 1H), 1.91 (m, 3H), 1.75 (m, 2H), 1.65 (m, 1H), 1.52 (m, 1H), 1.43 (m, 1H), 1.31 (m, 2H); ^13^C NMR (100 MHz, CDCl_3_) δ 169.6, 169.0, 147.2, 147.0, 145.2, 145.1, 138.7, 138.6, 137.5, 137.0, 128.2, 128.1, 127.9, 127.5, 126.4, 122.4, 122.3, 114.2, 114.0, 111.5, 111.4, 88.1, 87.6, 56.0, 49.9, 46.9, 46.3, 44.6, 44.43, 44.35, 36.2, 35.3, 32.1, 31.5, 31.4, 30.3, 25.4, 25.2, 25.0, 24.1, 21.2, 20.7, 11.6, 10.4; [α]^20^_D_ = +188.3° (CHCl_3_, c 1.11); ESI-MS 390.2 (M^+^ + H); HRMS (ES^+^) calcd for C_25_H_28_NO_3_ (M^+^ + H) 390.2064; found 390.2064.

((3S,6aR,11aR)-10-Methoxy-1,3,4,5,6,11a-hexahydro-2H-3,6a-methanobenzofuro[2,3-c]azocin-2-yl)((1S,2R)-2-phenylcyclopropyl)methanone **((-)-11)**: clear oil; yield 91.2%; ^1^H NMR (400 MHz, CDCl_3_): δ 7.14 (m, 5H), 6.87 (m, 1H), 6.76 (m, 1H), 6.65 (m, 1H), 4.80 (m, 1H), 4.61 (m, 1H), 3.96 (m, 1H), 3.90 (m, 3H), 3.44 (m, 1H), 2.50 (m, 1H), 2.14 (m, 1H), 1.91 (m, 3H), 1.75 (m, 2H), 1.65 (m, 1H), 1.52 (m, 1H), 1.43 (m, 1H), 1.32 (m, 2H); ^13^C NMR (100 MHz, CDCl_3_) δ 169.6, 169.0, 147.2, 147.0, 145.2, 145.1, 138.7, 138.6, 137.5, 137.0, 128.2, 128.1, 127.9, 127.5, 126.4, 122.4, 122.2, 114.2, 114.0, 111.5, 111.4, 88.1, 87.6, 56.0, 49.9, 46.8, 46.6, 44.6, 44.4, 44.3, 36.2, 35.3, 32.1, 31.5, 31.4, 30.3, 25.4, 25.2, 25.0, 24.1, 21.2, 20.7, 11.6, 10.4; [α]^20^_D_ = −189.6° (CHCl_3_, c 1.12); ESI-MS 390.2 (M^+^ + H); HRMS (ES^+^) calcd for C_25_H_28_NO_3_ (M^+^ + H) 390.2064; found 390.2065.

*General procedure for synthesis of tertiary amines* (**(+)-** *and* **(-)-12** *through* **15**) via *LiAlH*_4_ *reduction of amides*. To a solution of amides in THF (10 mL) at 0 °C under N_2_ was added dropwise a solution of LiAlH_4_ (3 eq) in THF (1 M, purchased from Sigma-Aldrich). The resulting solutions was warmed to room temperature and heated to 80 °C for 3 h. The solution was cooled to 0 °C and the reaction was quenched with a solution of Rochelle salt (1 M). The organic solvent was evaporated under reduced pressure and the aqueous mixture was extracted with CH_2_Cl_2_ (3 × 15 mL) and the combined extracts were washed with brine and dried over Na_2_SO_4_. After filtration and evaporation, the crude product was purified by flash chromatography (10–30% EtOAc in hexane) to give the corresponding tertiary amines.

(3R,6aS,11aS)-10-Methoxy-2-(((1S,2S)-2-phenylcyclopropyl)methyl)-1,3,4,5,6,11a-hexahydro-2H-3,6a-methanobenzofuro[2,3-c]azocine **((+)-12)**: clear oil; yield 89.2%; ^1^H NMR (400 MHz, CDCl_3_): δ 7.26 (m, 2H), 7.15 (m, 1H), 7.06 (m, 2H), 6.88 (t, *J* = 7.2 Hz, 1H), 6.75 (m, 2H), 4.30 (m, 1H), 3.88 (s, 3H), 3.57 (m, 1H), 3.36 (t, *J* = 10.8 Hz, 1H), 3.26 (s, 1H), 3.03 (d, *J* = 12.4 Hz, 1H), 2.55 (t, *J* = 13.2 Hz, 1H), 2.21 (d, *J* = 12.8 Hz, 1H), 2.14 (d, *J* = 12.0 Hz, 1H), 2.02 (d, *J* = 12.0 Hz, 1H), 1.76 (m, 3H), 1.62 (m, 1H), 1.46 (m, 2H), 1.28 (m, 1H), 0.96 (m, 1H), 0.87 (m, 1H); ^13^C NMR (100 MHz, CDCl_3_) δ 147.4, 145.1, 142.8, 139.8, 128.4 (2), 125.7 (2), 125.6, 121.9, 114.1, 111.0, 89.4, 59.2, 55.9, 53.7, 51.0, 44.6, 36.9, 32.0, 26.8, 23.6, 22.3, 21.8, 14.4; [α]^20^_D_ = +139.1° (CHCl_3_, c 1.15); ESI-MS 376.2 (M^+^ + H); HRMS (ES^+^) calcd for C_25_H_30_NO_2_ (M^+^ + H) 376.2271; found 376.2272.

(3S,6aR,11aR)-10-Methoxy-2-(((1R,2R)-2-phenylcyclopropyl)methyl)-1,3,4,5,6,11a-hexahydro-2H-3,6a-methanobenzofuro[2,3-c]azocine **((-)-12)**: clear oil; yield 88.7%; ^1^H NMR (400 MHz, CDCl_3_): δ 7.26 (m, 2H), 7.16 (m, 1H), 7.06 (m, 2H), 6.88 (t, J = 7.6 Hz, 1H), 6.75 (m, 2H), 4.30 (m, 1H), 3.88 (s, 3H), 3.57 (m, 1H), 3.36 (t, J = 10.8 Hz, 1H), 3.26 (s, 1H), 3.03 (d, J = 12.4 Hz, 1H), 2.56 (t, J = 10.8 Hz, 1H), 2.21 (d, J = 13.2 Hz, 1H), 2.14 (d, J = 12.0 Hz, 1H), 2.02 (d, J = 12.0 Hz, 1H), 1.76 (m, 3H), 1.62 (m, 1H), 1.46 (m, 2H), 1.28 (m, 1H), 0.96 (m, 1H), 0.87 (m, 1H); ^13^C NMR (100 MHz, CDCl_3_) δ 147.5, 145.1, 142.8, 139.8, 128.4 (2), 125.7 (2), 125.6, 121.9, 114.1, 111.0, 89.4, 59.2, 55.9, 53.7, 51.0, 44.6, 36.9, 32.0, 26.8, 23.6, 22.3, 21.8, 14.4; [α]^20^_D_ = −136.8° (CHCl_3_, c 0.71); ESI-MS 376.2 (M^+^ + H); HRMS (ES^+^) calcd for C_25_H_30_NO_2_ (M^+^ + H) 376.2271; found 376.2269.

(3S,6aR,11aR)-10-Methoxy-2-(((1S,2S)-2-phenylcyclopropyl)methyl)-1,3,4,5,6,11a-hexahydro-2H-3,6a-methanobenzofuro[2,3-c]azocine **((+)-13)**: clear oil; yield 87.8%; ^1^H NMR (400 MHz, CDCl_3_): δ 7.24 (t, J = 7.2 Hz, 2H), 7.14 (t, J = 7.2 Hz, 1H), 7.05 (d, J = 7.2 Hz, 2H), 6.87 (t, J = 8.0 Hz, 1H), 6.75 (t, J = 8.0 Hz, 1H), 6.72 (t, J = 7.2 Hz, 1H), 4.32 (dd, J = 11.6, 5.2 Hz, 1H), 3.88 (s, 3H), 3.56 (m, 1H), 3.37 (t, J = 11.2 Hz, 1H), 3.25 (s, 1H), 2.91 (dd, J = 12.4, 6.0 Hz, 1H), 2.67 (dd, J = 12.4, 6.8 Hz, 1H), 2.22 (d, J = 12.8 Hz, 1H), 2.11 (d, J = 12.0 Hz, 1H), 1.98 (d, J = 11.6 Hz, 1H), 1.76 (m, 3H), 1.60 (m, 1H), 1.45 (m, 2H), 1.26 (m, 1H), 1.00 (m, 1H), 0.88 (m, 1H); ^13^C NMR (100 MHz, CDCl_3_) δ 147.5, 145.2, 143.0, 139.9, 128.4 (2), 125.8 (2), 125.5, 121.9, 114.2, 111.1, 89.3, 58.9, 56.0, 52.8, 51.5, 44.6, 36.9, 32.0, 26.7, 22.4, 22.0, 21.8, 15.6; [α]^20^_D_ = +40.1° (CHCl_3_, c 1.0); ESI-MS 376.2 (M^+^ + H); HRMS (ES^+^) calcd for C_25_H_30_NO_2_ (M^+^ + H) 376.2271; found 376.2272.

(3R,6aS,11aS)-10-Methoxy-2-(((1R,2R)-2-phenylcyclopropyl)methyl)-1,3,4,5,6,11a-hexahydro-2H-3,6a-methanobenzofuro[2,3-c]azocine **((-)-13)**: clear oil; yield 75.0%; ^1^H NMR (400 MHz, CDCl_3_): δ 7.25 (m, 2H), 7.15 (t, J = 7.2 Hz, 1H), 7.06 (d, J = 7.2 Hz 2H), 6.88 (t, J = 7.2 Hz, 1H), 6.77 (d, J = 8.0 Hz, 1H), 6.73 (d, J = 6.8 Hz, 1H), 4.34 (m, 1H), 3.89 (s, 3H), 3.57 (m, 1H), 3.34 (t, J = 10.8 Hz, 1H), 3.26 (s, 1H), 2.92 (dd, J = 12.0, 6.8 Hz, 1H), 2.68 (dd, J = 12.0, 6.4 Hz, 1H), 2.23 (d, J = 12.8 Hz, 1H), 2.13 (d, J = 12.0 Hz, 1H), 1.99 (d, J = 12.0 Hz, 1H), 1.76 (m, 3H), 1.62 (m, 1H), 1.45 (m, 2H), 1.27 (m, 1H), 1.01 (m, 1H), 0.89 (m, 1H); ^13^C NMR (100 MHz, CDCl_3_) δ 147.5, 145.2, 143.0, 139.9, 128.4 (2), 125.8 (2), 125.6, 121.9, 114.2, 111.1, 89.4, 58.9, 56.0, 53.6, 51.5, 44.6, 36.9, 32.0, 26.7, 22.5, 22.0, 21.8, 15.6; [α]^20^_D_ = −46.3° (CHCl_3_, c 0.65); ESI-MS 376.2 (M^+^ + H); HRMS (ES^+^) calcd for C_25_H_30_NO_2_ (M^+^ + H) 376.2271; found 376.2272.

(3R,6aS,11aS)-10-Methoxy-2-(((1S,2R)-2-phenylcyclopropyl)methyl)-1,3,4,5,6,11a-hexahydro-2H-3,6a-methanobenzofuro[2,3-c]azocine **((+)-14)**: colorless oil; yield 91.4%; ^1^H NMR (400 MHz, CDCl_3_): δ 7.27 (t, J = 7.6 Hz, 2H), 7.20 (m, 3H), 6.86 (t, J = 8.0 Hz, 1H), 6.76 (dd, J = 8.0, 0.8 Hz, 1H), 6.70 (dd, J = 7.2, 0.8 Hz, 1H), 4.22 (dd, J = 12.0, 5.2 Hz, 1H), 3.88 (s, 3H), 3.29 (dd, J = 10.4, 5.6 Hz, 1H), 3.19 (m, 1H), 3.08 (t, J = 11.6 Hz, 1H), 2.55 (dd, J = 13.2, 6.4 Hz, 1H), 2.41 (dd, J = 13.2, 6.4 Hz, 1H), 2.22 (m, 1H), 2.05 (m, 2H), 1.93 (m, 1H), 1.68 (m, 1H), 1.51 (m, 2H), 1.36 (m, 3H), 1.09 (m, 1H), 0.86 (m, 1H); ^13^C NMR (100 MHz, CDCl_3_) δ 147.5, 145.2, 140.0, 138.8, 129.0 (2), 128.0 (2), 126.0, 121.9, 114.2, 111.0, 89.4, 56.0, 53.0, 52.5, 51.0, 44.6, 36.7, 31.9, 26.6, 21.6, 21.0, 18.0, 9.0; [α]^20^_D_ = +29.0° (CHCl_3_, c 0.82); ESI-MS 376.2 (M^+^ + H); HRMS (ES^+^) calcd for C_25_H_30_NO_2_ (M^+^ + H) 376.2271; found 376.2271.

(3S,6aR,11aR)-10-Methoxy-2-(((1R,2S)-2-phenylcyclopropyl)methyl)-1,3,4,5,6,11a-hexahydro-2H-3,6a-methanobenzofuro[2,3-c]azocine (**(-)-14**): colorless oil; yield 90.6%; ^1^H NMR (400 MHz, CDCl_3_): δ 7.26 (t, J = 7.6 Hz, 2H), 7.19 (m, 3H), 6.85 (t, J = 8.0 Hz, 1H), 6.74 (d, J = 8.0 Hz, 1H), 6.69 (d, J = 7.6 Hz, 1H), 4.21 (dd, J = 12.0, 5.2 Hz, 1H), 3.87 (s, 3H), 3.27 (dd, J = 10.4, 5.6 Hz, 1H), 3.17 (m, 1H), 3.07 (t, J = 11.2 Hz, 1H), 2.53 (dd, J = 12.6, 6.4 Hz, 1H), 2.40 (dd, J = 12.6, 6.4 Hz, 1H), 2.21 (m, 1H), 2.05 (m, 2H), 1.91 (m, 1H), 1.67 (dd, J = 14.0, 5.6 Hz, 1H), 1.51 (m, 2H), 1.36 (m, 3H), 1.07 (m, 1H), 0.84 (m, 1H); ^13^C NMR (100 MHz, CDCl_3_) δ 147.4, 145.1, 140.0, 138.8, 129.0 (2), 128.0 (2), 125.9, 121.8, 114.1, 111.0, 89.4, 55.9, 52.9, 52.4, 50.9, 44.5, 36.7, 32.0, 26.5, 21.6, 21.0, 18.0, 9.0; [α]^20^_D_ = −29.4° (CHCl_3_, c 1.05); ESI-MS 376.2 (M^+^ + H); HRMS (ES^+^) calcd for C_25_H_30_NO_2_ (M^+^ + H) 376.2271; found 376.2275.

(3S,6aR,11aR)-10-Methoxy-2-(((1S,2R)-2-phenylcyclopropyl)methyl)-1,3,4,5,6,11a-hexahydro-2H-3,6a-methanobenzofuro[2,3-c]azocine **((+)-15)**: clear oil; yield 91.7%; ^1^H NMR (400 MHz, CDCl_3_): δ 7.25 (m, 4H), 7.17 (m, 1H), 6.86 (t, J = 8.0 Hz, 1H), 6.76 (dd, J = 8.0, 0.8 Hz, 1H), 6.70 (dd, J = 7.2, 0.8 Hz, 1H), 4.28 (dd, J = 12.0, 5.6 Hz, 1H), 3.88 (s, 3H), 3.55 (dd, J = 10.0, 5.6 Hz, 1H), 3.27 (m, 1H), 2.93 (s, 1H), 2.63 (dd, J = 12.6, 5.6 Hz, 1H), 2.19 (m, 2H), 2.02 (dd, J = 12.0, 2.0 Hz, 1H), 1.92 (m, 1H), 1.84 (m, 1H), 1.70 (m, 2H), 1.48 (m, 1H), 1.32 (m, 3H), 1.11 (m, 1H), 0.85 (m, 1H); ^13^C NMR (100 MHz, CDCl_3_) δ 147.5, 145.2, 139.9, 139.0, 129.1 (2), 128.0 (2), 125.9, 121.8, 114.1, 111.1, 89.3, 56.0, 54.3, 53.4, 50.8, 44.5, 36.7, 31.9, 26.6, 21.7, 20.4, 17.6, 10.1; [α]^20^_D_ = +11.0° (CHCl_3_, c 0.7); ESI-MS 376.2 (M^+^ + H); HRMS (ES^+^) calcd for C_25_H_30_NO_2_ (M^+^ + H) 376.2271; found 376.2271.

(3R,6aS,11aS)-10-Methoxy-2-(((1R,2S)-2-phenylcyclopropyl)methyl)-1,3,4,5,6,11a-hexahydro-2H-3,6a-methanobenzofuro[2,3-c]azocine **((-)-15)**: clear oil; yield 87.5%; ^1^H NMR (400 MHz, CDCl_3_): δ 7.25 (m, 4H), 7.17 (m, 1H), 6.86 (t, J = 7.6 Hz, 1H), 6.76 (d, J = 8.0 Hz, 1H), 6.70 (d, J = 7.6 Hz, 1H), 4.28 (dd, J = 12.0, 5.6 Hz, 1H), 3.88 (s, 3H), 3.55 (dd, J = 10.0, 5.6 Hz, 1H), 3.26 (m, 1H), 2.93 (s, 1H), 2.63 (dd, J = 12.6, 5.6 Hz, 1H), 2.19 (m, 2H), 2.02 (dd, J = 12.0, 1.6 Hz, 1H), 1.92 (m, 1H), 1.84 (m, 1H), 1.70 (m, 2H), 1.48 (m, 1H), 1.32 (m, 3H), 1.11 (m, 1H), 0.85 (m, 1H); ^13^C NMR (100 MHz, CDCl_3_) δ 147.5, 145.2, 140.0, 139.0, 129.2 (2), 128.0 (2), 125.9, 121.8, 114.2, 111.2, 89.4, 56.0, 54.4, 53.4, 50.8, 44.5, 36.7, 31.9, 26.7, 21.8, 20.4, 17.6, 10.1; [α]^20^_D_ = −10.9° (CHCl_3_, c 0.58); ESI-MS 376.2 (M^+^ + H); HRMS (ES^+^) calcd for C_25_H_30_NO_2_ (M^+^ + H) 376.2271; found 376.2271.

*General procedure for synthesis of (***(+)-***and***(-)-16***through***19***)* via *O-demethylation of aryl methyl ether with BBr_3_*. To a solution of BBr_3_ (5 eq.) in CHCl_3_ (10 mL) at −78 °C under N_2_ was added a solution of the arylmethyl ethers and the resulting solution was warmed to rt gradually and stirred for 1 h at rt. The solution was cooled to −78 °C and the reaction was quenched with 28% NH_4_OH. The mixture was extracted with CHCl_3_ (3 × 10 mL) and the combined extracts were washed with brine and dried over anhydrous Na_2_SO_4_. After filtration and evaporation, the crude product was purified by flash chromatography (10–40% EtOAc in hexane) to the corresponding phenols.

(3R,6aS,11aS)-2-(((1S,2S)-2-Phenylcyclopropyl)methyl)-1,3,4,5,6,11a-hexahydro-2H-3,6a-methanobenzofuro[2,3-c]azocin-10-ol **((+)-16)**: white foam; yield 83.0%; Mp 246.6–251.6 °C (HCl salt); ^1^H NMR (400 MHz, CDCl_3_): δ 7.25 (m, 2H), 7.16 (m, 1H), 7.08 (d, *J* = 7.2 Hz 2H), 6.72 (m, 2H), 6.58 (d, *J* = 6.0 Hz, 1H), 4.00 (d, *J* = 11.6 Hz 1H), 3.62 (m, 1H), 3.25 (s, 1H), 3.12 (d, *J* = 12.4 Hz, 1H), 2.99 (t, *J* = 11.2 Hz, 1H), 2.44 (t, *J* = 10.0 Hz, 1H), 2.21 (d, *J* = 12.4 Hz, 1H), 2.06 (d, *J* = 11.6 Hz, 1H), 1.88 (d, *J* = 12.0 Hz, 1H), 1.72 (m, 3H), 1.65 (m, 1H), 1.47 (m, 3H), 0.89 (m, 2H); ^13^C NMR (100 MHz, CDCl_3_) δ 146.7, 142.7, 141.6, 139.6, 128.3(2), 126.1(2), 125.5, 122.2, 116.5, 113.6, 89.4, 59.2, 54.1, 50.3, 44.1, 37.2, 31.2, 26.1, 24.3, 21.4, 20.9, 14.3; [α]^20^_D_ = +122.7° (CHCl_3_, c 0.96); ESI-MS 362.2 (M^+^ + H); HRMS (ES^+^) calcd for C_24_H_28_NO_2_ (M^+^ + H) 362.2115; found 362.2113; the free was converted into HCl salt which was crystallized in MeOH and Et_2_O. Anal. Calcd for C_24_H_27_NO_2_•HCl•H_2_O: C 69.30, H 7.27, N 3.37; found C 69.41, H 7.45, N 3.31.

(3S,6aR,11aR)-2-(((1R,2R)-2-Phenylcyclopropyl)methyl)-1,3,4,5,6,11a-hexahydro-2H-3,6a-methanobenzofuro[2,3-c]azocin-10-ol **((-)-16)**: clear oil; yield 88.6%; Mp 252.7–256.5 °C (HCl salt); ^1^H NMR (400 MHz, CDCl_3_): δ 7.26 (m, 2H), 7.17 (m, 1H), 7.08 (d, *J* = 6.4 Hz 2H), 6.73 (m, 2H), 6.58 (d, *J* = 6.0 Hz, 1H), 4.00 (d, *J* = 8.4 Hz 1H), 3.63 (m, 1H), 3.26 (s, 1H), 3.13 (d, *J* = 12.0 Hz, 1H), 2.97 (t, *J* = 10.8 Hz, 1H), 2.44 (t, *J* = 10.0 Hz, 1H), 2.21 (d, *J* = 12.4 Hz, 1H), 2.06 (d, *J* = 12.0 Hz, 1H), 1.88 (d, *J* = 12.0 Hz, 1H), 1.73 (m, 3H), 1.66 (m, 1H), 1.48 (m, 3H), 0.89 (m, 2H); ^13^C NMR (100 MHz, CDCl_3_) δ 146.7, 142.7, 141.6, 139.6, 128.3(2), 126.1(2), 125.5, 122.2, 116.6, 113.6, 89.4, 59.2, 54.1, 50.3, 44.0, 37.1, 31.1, 26.0, 24.3, 21.4, 20.8, 14.3; [α]^20^_D_ = −121.5° (CHCl_3_, c 0.92); ESI-MS 362.2 (M^+^ + H); HRMS (ES^+^) calcd for C_24_H_28_NO_2_ (M^+^ + H) 362.2115; found 362.2114; the free was converted into HCl salt which was crystallized in MeOH and Et_2_O. Anal. Calcd for C_24_H_27_NO_2_•HCl•0.6H_2_O: C 70.52, H 7.20, N 3.43; found C 70.46, H 7.35, N 3.45.

(3S,6aR,11aR)-2-(((1S,2S)-2-Phenylcyclopropyl)methyl)-1,3,4,5,6,11a-hexahydro-2H-3,6a-methanobenzofuro[2,3-c]azocin-10-ol **((+)-17)**: white foam; yield 81.4%; Mp 187.2–192.2 °C (HCl salt); ^1^H NMR (400 MHz, CDCl_3_): δ 7.25 (m, 2H), 7.17 (m, 1H), 7.06 (d, *J* = 6.4 Hz 2H), 6.77 (t, *J* = 6.8 Hz, 1H), 6.71 (d, *J* = 6.8 Hz, 1H), 6.62 (d, *J* = 6.0 Hz, 1H), 4.30 (d, *J* = 8.4 Hz 1H), 3.65 (m, 1H), 3.37 (m, 2H), 2.97 (m, 1H), 2.74 (m, 1H), 2.28 (d, *J* = 12.0 Hz, 1H), 2.11 (d, *J* = 12.0 Hz, 1H), 2.03 (d, *J* = 11.2 Hz, 1H), 1.79 (m, 3H), 1.65 (m, 1H), 1.49 (m, 2H), 1.36 (m, 1H), 1.02 (m, 1H), 0.93 (m, 1H); ^13^C NMR (100 MHz, CDCl_3_) δ 146.5, 142.7, 141.6, 139.5, 128.4(2), 125.8(2), 125.6, 122.2, 116.4, 113.3, 88.6, 58.6, 52.3, 51.5, 44.3, 36.4, 31.6, 26.2, 22.3, 21.6, 21.4, 15.5; [α]^20^_D_ = +23.6° (CHCl_3_, c 0.9); ESI-MS 362.2 (M^+^ + H); HRMS (ES^+^) calcd for C_24_H_28_NO_2_ (M^+^ + H) 362.2115; found 362.2113; the free was converted into HCl salt which was crystallized in MeOH and Et_2_O. Anal. Calcd for C_24_H_27_NO_2_•HCl•H_2_O: C 69.30, H 7.27, N 3.37; found C 69.38, H 7.05, N 3.44.

(3R,6aS,11aS)-2-(((1R,2R)-2-Phenylcyclopropyl)methyl)-1,3,4,5,6,11a-hexahydro-2H-3,6a-methanobenzofuro[2,3-c]azocin-10-ol (**(-)-17)**: clear oil; yield 87.0%; Mp 208.2–213.2 °C (HCl salt); ^1^H NMR (400 MHz, CDCl_3_): δ 7.26 (t, *J* = 6.8 Hz, 2H), 7.16 (t, *J* = 7.2 Hz, 1H), 7.06 (d, *J* = 7.2 Hz 2H), 6.77 (t, *J* = 7.2 Hz, 1H), 6.71 (d, *J* = 7.6 Hz, 1H), 6.62 (d, *J* = 6.4 Hz, 1H), 4.28 (d, *J* = 10.8 Hz 1H), 3.64 (m, 1H), 3.36 (m, 2H), 2.98 (m, 1H), 2.74 (m, 1H), 2.28 (d, *J* = 12.8 Hz, 1H), 2.11 (d, *J* = 12.0 Hz, 1H), 2.00 (d, *J* = 12.0 Hz, 1H), 1.79 (m, 3H), 1.65 (m, 1H), 1.49 (m, 2H), 1.33 (m, 1H), 1.03 (d, *J* = 4.0 Hz, 1H), 0.93 (d, *J* = 4.0 Hz, 1H); ^13^C NMR (100 MHz, CDCl_3_) δ 146.5, 142.8, 141.6, 139.6, 128.4(2), 125.8(2), 125.6, 122.2, 116.3, 113.5, 88.9, 58.7, 52.5, 51.5, 44.4, 36.6, 31.7, 26.2, 22.3, 21.7, 21.6, 15.6; [α]^20^_D_ = −229.6° (CHCl_3_, c 0.63); ESI-MS 362.2 (M^+^ + H); HRMS (ES^+^) calcd for C_24_H_28_NO_2_ (M^+^ + H) 362.2115; found 362.2115; the free was converted into HCl salt which was crystallized in MeOH and Et_2_O. Anal. Calcd for C_24_H_27_NO_2_•HCl•0.5H_2_O: C 70.84, H 7.18, N 3.44; found C 70.71, H 7.33, N 3.46.

(3R,6aS,11aS)-2-(((1S,2R)-2-Phenylcyclopropyl)methyl)-1,3,4,5,6,11a-hexahydro-2H-3,6a-methanobenzofuro[2,3-c]azocin-10-ol **((+)-18)**: light yellow syrup; yield 84.8%; Mp 204.4–208.5 °C (HCl salt); ^1^H NMR (400 MHz, CDCl_3_): δ 7.29 (t, *J* = 7.6 Hz, 2H), 7.20 (m, 3H), 6.74 (m, 2H), 6.58 (dd, *J* = 7.2, 1.6 Hz, 1H), 4.20 (dd, *J* = 12.0, 5.6 Hz, 1H), 3.45 (dd, *J* = 10.4, 5.6 Hz, 1H), 3.35 (s, 1H), 3.01 (d, *J* = 11.2 Hz, 1H), 2.68 (dd, *J* = 12.6, 5.6 Hz, 1H), 2.42 (dd, *J* = 12.6, 7.2 Hz, 1H), 2.25 (m, 1H), 2.06 (m, 2H), 1.98 (d, *J* = 12.6 Hz, 1H), 1.69 (d, *J* = 10.0 Hz, 1H), 1.47 (m, 5H), 1.12 (m, 1H), 0.91 (m, 1H); ^13^C NMR (100 MHz, CDCl_3_) δ 146.5, 141.6, 139.4, 138.6, 128.9 (2), 128.1 (2), 126.1, 122.2, 116.3, 113.4, 88.6, 53.2, 52.8, 51.0, 44.2, 36.2, 31.6, 25.9, 21.3, 20.8, 17.0, 9.2; [α]^20^_D_ = +16.3° (CHCl_3_, c 0.7); ESI-MS 362.2 (M^+^ + H); HRMS (ES^+^) calcd for C_24_H_28_NO_2_ (M^+^ + H) 362.2115; found 362.2113; Anal. Calcd for C_24_H_27_NO_2_•HCl•H_2_O: C 69.30, H 7.27, N 3.37; found C 69.58, H 7.67, N 3.32.

(3S,6aR,11aR)-2-(((1R,2S)-2-Phenylcyclopropyl)methyl)-1,3,4,5,6,11a-hexahydro-2H-3,6a-methanobenzofuro[2,3-c]azocin-10-ol **((-)-18)**: white foam; yield 80.4%; Mp 193.9–198.5 °C (HCl salt); ^1^H NMR (400 MHz, CDCl_3_): 7.29 (t, *J* = 7.6 Hz, 2H), 7.20 (m, 3H), 6.76 (t, *J* = 7.6 Hz, 1H), 6.70 (d, *J* = 8.0 Hz, 1H), 6.59 (d, *J* = 7.2 Hz, 1H), 4.17 (dd, *J* = 12.0, 6.0 Hz, 1H), 3.41 (dd, *J* = 9.6, 5.2 Hz, 1H), 3.32 (s, 1H), 3.02 (d, *J* = 11.2 Hz, 1H), 2.67 (dd, *J* = 12.6, 5.2 Hz, 1H), 2.37 (dd, *J* = 12.6, 7.2 Hz, 1H), 2.23 (m, 1H), 2.06 (m, 2H), 1.93 (d, *J* = 12.0 Hz, 1H), 1.70 (d, *J* = 13.2 Hz, 1H), 1.53 (m, 2H), 1.42 (m, 3H), 1.11 (m, 1H), 0.90 (m, 1H); ^13^C NMR (100 MHz, CDCl_3_) δ 146.6, 141.6, 139.7, 138.7, 129.0 (2), 128.1 (2), 126.0, 122.2, 116.3, 113.4, 89.0, 53.2, 52.3, 51.0, 44.3, 36.5, 31.6, 26.0, 21.4, 20.7, 17.2, 9.3; [α]^20^_D_ = −16.7° (CHCl_3_, c 1.05); ESI-MS 362.2 (M^+^ + H); HRMS (ES^+^) calcd for C_24_H_28_NO_2_ (M^+^ + H) 362.2115; found 362.2114; the free was converted into HCl salt which was crystallized in *^i^*PrOH and Et_2_O. Anal. Calcd for C_24_H_27_NO_2_•HCl•0.8*^i^*PrOH: C 71.09, H 7.77, N 3.14; Found C 70.82, H 7.98, N 3.41.

(3S,6aR,11aR)-2-(((1S,2R)-2-Phenylcyclopropyl)methyl)-1,3,4,5,6,11a-hexahydro-2H-3,6a-methanobenzofuro[2,3-c]azocin-10-ol **((+)-19)**: light yellow syrup; yield 76.7%; Mp 183.1–187.7 °C (HCl salt); ^1^H NMR (400 MHz, CDCl_3_): δ 7.24 (m, 5H), 6.77 (t, *J* = 8.0 Hz, 1H), 6.72 (d, *J* = 7.2 Hz, 1H), 6.60 (d, *J* = 6.8 Hz, 1H), 4.22 (dd, *J* = 11.6, 4.8 Hz, 1H), 3.58 (dd, *J* = 10.0, 5.6 Hz, 1H), 3.24 (t, *J* = 11.2 Hz, 1H), 3.09 (s, 1H), 2.75 (dd, *J* = 12.8, 4.8 Hz, 1H), 2.17 (m, 2H), 2.04 (d, *J* = 11.2 Hz, 1H), 1.96 (d, *J* = 11.6 Hz, 1H), 1.89 (d, *J* = 9.2 Hz, 1H), 1.71 (m, 2H), 1.53 (m, 1H), 1.34 (m, 3H), 1.14 (m, 1H), 0.91 (m, 1H); ^13^C NMR (100 MHz, CDCl_3_) δ 146.4, 141.4, 139.6, 138.8, 129.2 (2), 128.1 (2), 126.1, 122.2, 115.9, 113.6, 88.9, 54.6, 53.4, 51.0, 44.4, 36.4, 31.7, 26.3, 21.6, 20.4, 17.0, 10.3; [α]^20^_D_ = +9.9° (CHCl_3_, c 0.96); ESI-MS 362.2 (M^+^ + H); HRMS (ES^+^) calcd for C_24_H_28_NO_2_ (M^+^ + H) 362.2115; found 362.2114; Anal. Calcd for C_24_H_27_NO_2_•HCl•0.6H_2_O: C 70.52, H 7.20, N 3.43; found C 70.62, H 7.54, N 3.44.

(3R,6aS,11aS)-2-(((1R,2S)-2-phenylcyclopropyl)methyl)-1,3,4,5,6,11a-hexahydro-2H-3,6a-methanobenzofuro[2,3-c]azocin-10-ol **((-)-19)**: colorless syrup; yield 90.4%; Mp 193.3–198.3 °C (HBr salt); ^1^H NMR (400 MHz, CDCl_3_): δ 7.24 (m, 5H), 6.77 (t, *J* = 8.0 Hz, 1H), 6.72 (d, *J* = 8.0 Hz, 1H), 6.61 (d, *J* = 7.2 Hz, 1H), 4.21 (dd, *J* = 12.0, 5.2 Hz, 1H), 3.55 (dd, *J* = 10.0, 5.2 Hz, 1H), 3.23 (t, *J* = 11.6 Hz, 1H), 3.05 (s, 1H), 2.73 (dd, *J* = 12.8, 4.8 Hz, 1H), 2.16 (m, 2H), 2.03 (d, *J* = 12.0 Hz, 1H), 1.91 (m, 2H), 1.70 (m, 2H), 1.53 (m, 1H), 1.36 (m, 3H), 1.14 (m, 1H), 0.89 (m, 1H); ^13^C NMR (100 MHz, CDCl_3_) δ 146.4, 141.4, 139.7, 138.9, 129.2 (2), 128.1 (2), 126.0, 122.2, 115.8, 113.6, 89.2, 54.5, 53.3, 51.0, 44.5, 36.6, 31.8, 26.4, 21.7, 20.4, 17.1, 10.3; [α]^20^_D_ = +10.5° (CHCl_3_, c 1.6); ESI-MS 362.2 (M^+^ + H); HRMS (ES^+^) calcd for C_24_H_28_NO_2_ (M^+^ + H) 362.2115; found 362.2115; the free was converted into HBr salt which was crystallized in EtOH and Et_2_O. Anal. Calcd for C_24_H_27_NO_2_•HBr•EtOH: C 63.93, H 7.02, N 2.87; found C 63.95, H 7.31, N 3.03.

General procedure for synthesis of 2-(E)-cinnamyl-10-methoxy-1,3,4,5,6,11a-hexahydro-2H-3,6a-methanobenzofuro[2,3-c]azocine **(+)-** and **(-)-20**. A mixture of **(+)-** or **(-)-6** (80 mg, 0.33 mmol), K_2_CO_3_ (91 mg, 0.66 mmol), (E)-cinnamyl bromide (96 mg, 0.49 mmol) and DMF (10 mL) was stirred at rt overnight. The mixture was diluted with H_2_O (20 mL) and extracted with Et_2_O (3 × 20 mL). The combined extracts were washed with H_2_O and brined and dried over anhydrous Na_2_SO_4_. After filtration and evaporation, the crude product was purified by flash chromatography (10–30% EtOAc in hexane) to give 10-methoxy-2-phenethyl-1,3,4,5,6,11a-hexahydro-2H-3,6a-methanobenzofuro[2,3-c]-azocine **(+)-** or **(-)-20** as clear oil.

**(-)-20**: clear oil; 88.2%; ^1^H NMR (400 MHz, CDCl_3_): δ 7.40 (d, *J* = 7.6 Hz, 2H), 7.33 (t, *J* = 7.6 Hz, 2H), 7.24 (t, *J* = 7.6 Hz, 1H), 6.90 (t, *J* = 8.0 Hz, 1H), 6.79 (d, *J* = 8.0 Hz, 1H), 6.75 (d, *J* = 7.2 Hz, 1H), 6.61 (d, *J* = 16 Hz, 1H), 6.31 (dt, *J* = 15.6, 6.8 Hz, 1H), 4.34 (m, 1H), 3.90 (s, 3H), 3.60 (dd, *J* = 13.6, 6.8 Hz, 1H), 3.50 (dd, *J* = 13.6, 6.4 Hz, 1H), 3.44 (m, 2H), 3.25 (s, 1H), 2.25 (d, *J* = 12.8 Hz, 1H), 2.15 (dd, *J* = 12.4, 1.6 Hz, 1H), 2.03 (dt, *J* = 12.0, 2.0 Hz, 1H), 1.84 (m, 2H), 1.66 (m, 1H), 1.47 (m, 2H); ^13^C NMR (100 MHz, CDCl_3_) δ 147.5, 145.2, 139.8, 137.1, 132.2, 128.6 (2), 128.0, 127.5, 126.4 (2), 121.9, 114.2, 111.3, 89.2, 57.1, 56.0, 52.5, 51.5, 44.6, 36.8, 32.0, 26.6, 21.9; [α]^20^_D_ = −37.8° (CHCl_3_, c 1.2); ESI-MS 362.2 (M^+^ + H); HRMS (ES^+^) calcd for C_24_H_28_NO_2_ (M^+^ + H) 362.2115; found 362.2113.

**(+)-20**: clear oil; 83.3%; ^1^H NMR (400 MHz, CDCl_3_): δ 7.39 (d, *J* = 7.6 Hz, 2H), 7.32 (t, *J* = 7.6 Hz, 2H), 7.23 (t, *J* = 7.2 Hz, 1H), 6.89 (t, *J* = 7.6 Hz, 1H), 6.78 (d, *J* = 8.0 Hz, 1H), 6.74 (d, *J* = 7.2 Hz, 1H), 6.60 (d, *J* = 16 Hz, 1H), 6.30 (dt, *J* = 15.6, 6.8 Hz, 1H), 4.33 (m, 1H), 3.89 (s, 3H), 3.59 (dd, *J* = 13.6, 6.8 Hz, 1H), 3.49 (dd, *J* = 13.6, 6.4 Hz, 1H), 3.43 (m, 2H), 3.24 (s, 1H), 2.25 (d, *J* = 12.8 Hz, 1H), 2.15 (dd, *J* = 12.4, 1.6 Hz, 1H), 2.02 (dt, *J* = 12.4, 2.0 Hz, 1H), 1.86 (m, 2H), 1.66 (m, 1H), 1.44 (m, 2H); ^13^C NMR (100 MHz, CDCl_3_) δ 147.5, 145.2, 139.8, 137.1, 132.2, 128.7 (2), 128.0, 127.5, 126.4 (2), 121.9, 114.2, 111.3, 89.2, 57.1, 56.0, 52.6, 51.6, 44.6, 36.8, 32.0, 26.6, 21.9; [α]^20^_D_ = +37.4° (CHCl_3_, c 1.3); ESI-MS 362.2 (M^+^ + H); HRMS (ES^+^) calcd for C_24_H_28_NO_2_ (M^+^ + H) 362.2115; found 362.2116.

Compounds **(+)-** and **(-)- 21** were prepared through the same procedure as the synthesis of compounds **16**–**19** via O-demethylation with BBr_3_:

**(-)-21:** white solid; 87.1%; Mp 199.0–202.9 °C (HCl salt); ^1^H NMR (400 MHz, CDCl_3_+CD_3_OD): δ 7.34 (d, *J* = 7.2 Hz, 2H), 7.26 (t, *J* = 7.6 Hz, 2H), 7.18 (t, *J* = 7.2 Hz, 1H), 6.72 (t, *J* = 8.0 Hz, 1H), 6.60 (d, *J* = 8.0 Hz, 1H), 6.58 (d, *J* = 6.8 Hz, 1H), 6.55 (d, *J* = 15.6 Hz, 1H), 6.25 (dt, *J* = 15.6, 6.8 Hz, 1H), 4.22 (m, 1H), 3.52 (dd, *J* = 13.6, 6.8 Hz, 1H), 3.43 (dd, *J* = 13.6, 6.8 Hz, 1H), 3.32 (m, 2H), 3.18 (s, 1H), 2.19 (d, *J* = 12.8 Hz, 1H), 2.09 (d, *J* = 11.6 Hz, 1H), 1.95 (d, *J* = 12.0Hz, 1H), 1.75 (m, 2H), 1.60 (m, 1H), 1.42 (m, 2H); ^13^C NMR (100 MHz, CDCl_3_+CD_3_OD) δ 146.2, 141.6, 139.6, 136.9, 132.8, 128.6 (2), 127.6, 127.1, 126.4 (2), 122.0, 115.6, 113.2, 88.8, 57.1, 52.7, 51.4, 44.5, 36.5, 31.7, 26.4, 21.7; [α]^20^_D_ = −34.0° (CHCl_3_, c 0.99); ESI-MS 348.2 (M^+^ + H); HRMS (ES^+^) calcd for C_23_H_26_NO_2_ (M^+^ + H) 348.1958; found 348.1961; the free was converted into HCl salt which was crystallized in MeOH and Et_2_O. Anal. Calcd for C_24_H_27_NO_2_•HCl•0.8H_2_O: C 69.35, H 6.98, N 3.52; found C 69.49, H 7.28, N 3.55.

**(+)-21**: white foam; 88.5%; Mp 220–225 °C (HCl salt); ^1^H NMR (400 MHz, CDCl_3_+CD_3_OD): δ 7.34 (d, *J* = 7.6 Hz, 2H), 7.26 (t, *J* = 7.6 Hz, 2H), 7.18 (t, *J* = 7.2 Hz, 1H), 6.72 (t, *J* = 7.6 Hz, 1H), 6.66 (d, *J* = 8.0 Hz, 1H), 6.58 (d, *J* = 6.0 Hz, 1H), 6.50 (d, *J* = 15.2 Hz, 1H), 6.25 (dt, *J* = 16.0, 6.8 Hz, 1H), 4.22 (m, 1H), 3.52 (dd, *J* = 13.6, 6.8 Hz, 1H), 3.43 (dd, *J* = 13.6, 6.8 Hz, 1H), 3.33 (m, 2H), 3.19 (s, 1H), 2.20 (d, *J* = 13.6 Hz, 1H), 2.08 (d, *J* = 12.0 Hz, 1H), 1.96 (d, *J* = 12.4 Hz, 1H), 1.75 (m, 2H), 1.60 (m, 1H), 1.42 (m, 2H); ^13^C NMR (100 MHz, CDCl_3_+CD_3_OD) δ 146.2, 141.6, 139.5, 136.8, 132.8, 128.6 (2), 127.6, 127.0, 126.4 (2), 122.0, 115.6, 113.1, 88.7, 57.1, 52.7, 51.4, 44.4, 36.4, 31.7, 26.3, 21.6; [α]^20^_D_ = +33.6° (CHCl_3_, c 1.2); ESI-MS 348.2 (M^+^ + H); HRMS (ES^+^) calcd for C_23_H_26_NO_2_ (M^+^ + H) 348.1958; found 348.1959; the free was converted into HCl salt. Anal. Calcd for C_24_H_27_NO_2_•HCl•H_2_O•0.4CH_2_Cl_2_: C 64.48, H 6.66, N 3.21; found C 64.35, H 6.81, N 3.17.

**(-)-22**: clear oil; 92.3%; ^1^H NMR (400 MHz, CDCl_3_): δ 7.37 (m, 2H), 7.28 (m, 3H), 6.88 (m, 1H), 6.71 (m, 3H), 6.10 (m, 1H), 5.12 (m, 1H), 4.41 (m, 1H), 3.95 (m, 1H), 3.85 (m, 3H), 3.58 (m, 1H), 2.05 (m, 2H), 1.84 (m, 1H), 1.61 (m, 3H), 1.45 (m, 1H), 1.29 (m, 1H); ^13^C NMR (100 MHz, CDCl_3_) δ 169.7, 169.6, 147.2, 146.9, 145.2, 145.1, 138.4, 135.8, 135.3, 133.2, 133.1, 128.7, 128.6, 128.4, 128.2, 124.1, 123.7, 122.5, 122.4, 114.2, 114.1, 111.6, 111.4, 87.5, 87.4, 56.1, 56.0, 51.6, 47.7, 46.0, 44.9, 44.6, 43.5, 36.0, 35.5, 32.0, 31.3, 29.6, 21.04, 20.98; [α]^20^_D_ = −42.1° (CHCl_3_, c 1.2); ESI-MS 376.2 (M^+^ + H); HRMS (ES^+^) calcd for C_24_H_26_NO_3_ (M^+^ + H) 376.1907; found 376.1908.

**(+)-22**: clear oil; 85.7%; ^1^H NMR (400 MHz, CDCl_3_): δ 7.36 (m, 2H), 7.27 (m, 3H), 6.87 (m, 1H), 6.71 (m, 3H), 6.09 (m, 1H), 5.11 (m, 1H), 4.40 (m, 1H), 3.92 (m, 1H), 3.84 (m, 3H), 3.59 (m, 1H), 2.04 (m, 2H), 1.83 (m, 1H), 1.60 (m, 3H), 1.43 (m, 1H), 1.29 (m, 1H); ^13^C NMR (100 MHz, CDCl_3_) δ 169.6, 169.5, 147.2, 146.9, 145.2, 145.1, 138.42, 138.38, 135.8, 135.3, 133.2, 133.1, 128.64, 128.60, 128.57, 128.3, 128.1, 124.1, 123.7, 122.5, 122.3, 114.2, 114.0, 111.6, 111.4, 87.5, 87.3, 56.0, 55.9, 51.5, 47.6, 45.8, 44.9, 44.6, 43.5, 36.0, 35.5, 32.0, 31.3, 29.6, 21.0, 20.9; [α]^20^_D_ = +42.4° (CHCl_3_, c 1.06); ESI-MS 376.2 (M^+^ + H); HRMS (ES^+^) calcd for C_24_H_26_NO_3_ (M^+^ + H) 376.1907; found 376.1908.

**(-)-23**: white amorphous solid; 92.3%; ^1^H NMR (400 MHz, CDCl_3_): δ 7.37 (m, 2H), 7.28 (m, 3H), 6.68 (m, 4H), 6.11 (d, *J* = 12.8 Hz, 1H), 5.68 and 5.46 (brs, 1H, -OH), 5.13 (m, 1H), 4.38 (m, 1H), 3.92 (m, 1H), 3.59 (m, 1H), 2.06 (m, 2H), 1.84 (m, 1H), 1.61 (m, 3H), 1.46 (m, 1H), 1.40 (m, 1H); ^13^C NMR (100 MHz, CDCl_3_) δ 169.9, 169.8, 145.8, 145.6, 141.4, 141.2, 138.4, 138.2, 135.8, 135.3, 133.42, 133.39, 128.83, 128.77, 128.74, 128.71, 128.4, 128.2, 124.1, 123.5, 122.7, 122.6, 115.8, 115.6, 113.8, 113.6, 87.7, 87.6, 51.7, 47.7, 46.0, 45.1, 44.9, 43.6, 36.1, 35.6, 32.0, 31.3, 29.6, 21.1, 21.0; [α]^20^_D_ = −42.1° (CHCl_3_, c 1.2); ESI-MS 376.2 (M^+^ + H); HRMS (ES^+^) calcd for C_24_H_26_NO_3_ (M^+^ + H) 376.1907; found 376.1908.

**(+)-23**: white amorphous solid; 88.2%; ^1^H NMR (400 MHz, CDCl_3_): δ 7.36 (m, 2H), 7.28 (m, 3H), 6.68 (m, 4H), 6.10 (d, *J* = 12.8 Hz, 1H), 5.94 and 5.75 (brs, 1H, -OH), 5.13 (m, 1H), 4.38 (m, 1H), 3.91 (m, 1H), 3.59 (m, 1H), 2.07 (m, 2H), 1.82 (m, 1H), 1.65 (m, 3H), 1.40 (m, 2H); ^13^C NMR (100 MHz, CDCl_3_) δ 169.92, 169.88, 145.8, 145.7, 141.5, 141.3, 138.4, 138.2, 135.8, 135.3, 133.45, 133.42, 128.82, 128.76, 128.74, 128.70, 128.4, 128.2, 124.1, 123.5, 122.6, 122.6, 115.8, 115.7, 113.7, 113.5, 87.7, 87.6, 51.7, 47.8, 46.1, 45.1, 44.8, 43.6, 36.0, 35.6, 32.0, 31.3, 29.6, 21.1, 21.0; [α]^20^_D_ = +39.7° (CHCl_3_, c 0.76); ESI-MS 362.2 (M^+^ + H); HRMS (ES^+^) calcd for C_23_H_24_NO_3_ (M^+^ + H) 362.1751; found 362.1750.

**(-)-24**: clear oil; 33.3%; Mp 167–171 °C (HCl salt); ^1^H NMR (400 MHz, CDCl_3_+CD_3_OD): δ 7.36 (t, *J* = 8.0 Hz, 2H), 7.26 (m, 3H), 6.77 (t, *J* = 8.0 Hz, 1H), 6.71 (d, *J* = 7.2 Hz, 1H), 6.62 (m, 2H), 5.87 (m, 1H), 4.28 (dd, *J* = 12.0, 5.6 Hz, 1H), 3.68 (m, 2H), 3.44 (dd, *J* = 10.0, 5.6 Hz, 1H), 3.34 (t, *J* = 11.6 Hz, 1H), 3.27 (s, 1H), 2.11 (d, *J* = 12.0 Hz, 1H), 2.00 (m, 2H), 1.74 (m, 2H), 1.55 (m, 1H), 1.40 (m, 2H); ^13^C NMR (100 MHz, CDCl_3_+CD_3_OD) δ 146.2, 141.6, 139.4, 136.9, 131.8, 128.9 (2), 128.4, 128.3 (2), 127.1, 122.2, 115.7, 113.2, 88.6, 52.9, 52.1, 51.5, 44.4, 36.3, 31.6, 26.2, 21.6; [α]^20^_D_ = −14.9° (CHCl_3_, c 1.01); ESI-MS 376.2 (M^+^ + H); HRMS (ES^+^) calcd for C_23_H_26_NO_2_ (M^+^ + H) 348.1958; found 348.1959; the free was converted into HCl salt. Anal. Calcd for C_23_H_25_NO_2_•HCl•1.3H_2_O: C 67.82, H 7.08, N 3.44; found C 67.86, H 7.41, N 3.43.

**(+)-24**: light yellow oil; 25.8%; Mp 170–174.4 °C (HCl salt); ^1^H NMR (400 MHz, CDCl_3_): δ 7.36 (t, *J* = 8.0 Hz, 2H), 7.27 (m, 3H), 6.79 (t, *J* = 8.0 Hz, 1H), 6.73 (d, *J* = 8.0 Hz, 1H), 6.62 (m, 2H), 5.87 (m, 1H), 4.27 (dd, *J* = 12.0, 5.6 Hz, 1H), 3.66 (m, 2H), 3.45 (dd, *J* = 10.0, 5.6 Hz, 1H), 3.33 (t, *J* = 11.6 Hz, 1H), 3.25 (s, 1H), 2.10 (d, *J* = 12.0 Hz, 1H), 2.00 (m, 2H), 1.76 (m, 2H), 1.56 (m, 1H), 1.43 (m, 2H); ^13^C NMR (100 MHz, CDCl_3_) δ 146.3, 141.3, 139.6, 137.2, 131.5, 130.2, 129.0 (2), 128.3 (2), 127.1, 122.3, 115.7, 113.8, 89.4, 52.7, 52.2, 51.4, 44.6, 36.7, 31.8, 26.4, 21.8; [α]^20^_D_ = +14.5° (CHCl_3_, c 0.9); ESI-MS 348.2 (M^+^ + H); HRMS (ES^+^) calcd for C_23_H_26_NO_2_ (M^+^ + H) 348.1958; found 348.1960; the free was converted into HCl salt. Anal. Calcd for C_23_H_25_NO_2_•HCl•0.25CH_2_Cl_2_: C 68.93, H 6.59, N 3.46; found C 68.81, H 6.52, N 3.63.

**(+)-25:** clear oil; 84.7%; Mp 161–163 °C (HCl salt); ^1^H NMR (400 MHz, CDCl_3_): δ 7.28 (t, *J* = 8.0 Hz, 2H), 7.18 (m, 3H), 6.79 (t, *J* = 7.6 Hz, 1H), 6.73 (d, *J* = 7.6 Hz, 1H), 6.64 (d, *J* = 7.2 Hz, 1H), 4.26 (dd, *J* = 12.0, 5.6 Hz, 1H), 3.41 (dd, *J* = 10.0, 5.6 Hz, 1H), 3.32 (t, *J* = 11.6 Hz, 1H), 3.17 (s, 1H), 2.74 (m, 2H), 2.66 (t, *J* = 7.6 Hz, 2H), 2.18 (d, *J* = 13.2 Hz, 1H), 2.11 (dd, *J* = 12.4, 1.2 Hz, 1H), 1.90 (m, 3H), 1.75 (m, 2H), 1.62 (m, 1H), 1.45 (m, 2H); ^13^C NMR (100 MHz, CDCl_3_) δ 146.4, 142.2, 141.3, 139.7, 128.6 (2), 128.5 (2), 125.9, 122.2, 115.8, 113.8, 89.4, 53.8, 52.7, 51.3, 44.6, 36.8, 33.7, 31.8, 29.5, 26.4, 21.8; [α]^20^_D_ = +33.4° (CHCl_3_, c 0.94); ESI-MS 350.2 (M^+^ + H); HRMS (ES^+^) calcd for C_23_H_28_NO_2_ (M^+^ + H) 350.2115; found 350.2113; the free was converted into HCl salt. Anal. Calcd for C_23_H_27_NO_2_•HCl•0.7H_2_O: C 69.32, H 7.44, N 3.51; found C 69.41, H 7.57, N 3.42.

**(-)-25**: clear oil; 88.7%; Mp 163–166 °C (HCl salt); ^1^H NMR (400 MHz, CDCl_3_): δ 7.28 (t, *J* = 8.0 Hz, 2H), 7.19 (m, 3H), 6.77 (t, *J* = 8.0 Hz, 1H), 6.71 (d, *J* = 8.0 Hz, 1H), 6.62 (d, *J* = 7.2 Hz, 1H), 4.24 (dd, *J* = 12.0, 5.6 Hz, 1H), 3.44 (dd, *J* = 10.0, 5.6 Hz, 1H), 3.31 (t, *J* = 11.6 Hz, 1H), 3.19 (s, 1H), 2.76 (m, 2H), 2.66 (t, *J* = 8.0 Hz, 2H), 2.18 (d, *J* = 13.2 Hz, 1H), 2.10 (dd, *J* = 12.0, 1.2 Hz, 1H), 1.92 (m, 3H), 1.75 (m, 2H), 1.62 (m, 1H), 1.44 (m, 2H); ^13^C NMR (100 MHz, CDCl_3_) δ 146.4, 142.1, 141.4, 139.6, 128.5 (2), 128.4 (2), 125.9, 122.2, 116.1, 113.6, 89.1, 53.9, 52.6, 51.3, 44.5, 36.7, 33.8, 31.7, 29.3, 26.2, 21.8; [α]^20^_D_ = +33.4° (CHCl_3_, c 0.94); ESI-MS 350.2 (M^+^ + H); HRMS (ES^+^) calcd for C_23_H_28_NO_2_ (M^+^ + H) 350.2115; found 350.2114; the free was converted into HCl salt. Anal. Calcd for C_23_H_27_NO_2_•HCl•0.7H_2_O: C 69.32, H 7.44, N 3.51; found C 69.40, H 7.51, N 3.51.

### 3.3. In Vitro Pharmacology

#### 3.3.1. Opioid Receptor Binding Assays

Frozen whole rat brains excluding cerebellum were thawed on ice, homogenized in 50 mM Tris HCl, pH 7.5 using a Brinkman Polytron (setting 6 for 20 s), and centrifuged at 30,000× *g* for 10 min at 4 °C. The supernatant was discarded, and the pellet was re-suspended in a fresh buffer and spun at 30,000× *g* for 10 min. The supernatant was discarded, and the pellet was re-suspended to give 100 mg/mL original wet weight. Ligand binding experiments were conducted in polypropylene assay tubes containing 0.5 mL Tris HCl buffer for 60 min at room temperature. [^3^H]DADLE (final concentration 1 nM, PolyPeptide Laboratories, San Diego, CA, USA), [^3^H]DAMGO (final concentration 1 nM, PolyPeptide Laboratories, San Diego, CA, USA) or [^3^H]U69,593 (final concentration 1 nM, Perkin Elmer Life Sciences, Waltham, MA, USA) were used to determine binding at δ-, μ- and κ-opioid receptor sites, respectively. Unlabeled DAMGO (final concentration, 30 nM) was added to the delta assay tubes to block μ-receptor binding. All assay tubes contained 100 μL homogenate suspension. Nonspecific binding was determined in all assays using 0.01 mM naloxone. Incubations were terminated by rapid filtration through Whatman GF/B filters, presoaked in 0.1% polyethyleneimine, using a Brandel R48 filtering manifold (Brandel Instruments Gaithersburg, Maryland). The filters were washed twice with 5 mL cold buffer and transferred to scintillation vials. Cytoscint (MP Biologicals) (3.0 mL) was added, and the vials were counted the next day using a Perkin Elmer TriCarb liquid scintillation counter. Data were analyzed with GraphPad Prism software (San Diego, CA, USA).

#### 3.3.2. Forskolin-Induced cAMP Accumulation Assays

Cell lines and cell culture: cAMP Hunter^TM^ Chinese hamster ovary cells (CHO-K1) that express human μ-opioid receptor (OPRM1), human κ-opioid receptor (OPRMK1), and human δ-receptor (OPRMD1) were purchased from Eurofins DiscoverX (Fremont, CA, USA). Cell culture was performed as previously described [18]. Briefly, cells were plated in a 384-well white tissue culture microplate at 10,000 cells/well density and incubated overnight at 37 °C in 5% CO_2_. Compounds were first dissolved in DMSO to form stock solutions (5 mM concentration), and then 9 doses of 100× solutions were prepared by serial dilution with DMSO. Then, 5× solutions were prepared by diluting 100× solutions with buffer consisting of Hank’s Buffered Salt Solution, HEPES, and forskolin. In the agonist assay, cells were treated with compounds (at 1× final concentration) and incubated at 37 °C for 30 min. In the antagonist assay [17], cells were pretreated with compounds for 15 min at 37 °C followed by 30 min incubation at 37 °C with selected agonists at their EC_50_ or EC_90_ dose. The HitHunter cAMP Assay for Small Molecules by Eurofins DiscoverX (Fremont, CA, USA) was then used according to manufacturer’s directions and the BioTek Synergy H1 hybrid plate reader (BioTek, Winooski, VT, USA) and Gen5 Software version 2.01 (BioTek, Winooski, VT, USA) were used to quantify luminescence [19].

### 3.4. Single-Crystal X-ray Diffraction Analysis of (R)-(-)-p-methylmandelate of (3S,6aR,11aR)-**(-)-**10-methoxy-1,3,4,5,6,11a-hexahydro-2H-3,6a-methano-benzofuro[2,3-c]azocine **(-)-**6

C_24_H_31_NO_6_, FW = 429.50, orthorhombic, P2_1_2_1_2_1_, a = 6.7324(6) Å, b = 9.4370(6) Å, c = 34.612(4) Å, *α* = 90°, *β* = 90°, *γ* = 90°, V = 2199.0(3) Å^3^, Z = 4, *ρ*_calc_ (293K) = 1.297 Mg/m^3^, *μ* = 0.759 mm^−1^, *F*(000) = 920, R_1_ = 0.0466 for 4249 observed (I > 2σI) reflections and 0.0480 for all 4436 reflections, Goodness-of-fit = 1.091, 291 parameters.

A colorless plate crystal of dimensions 0.211 × 0.102 × 0.040 mm was mounted on a MiteGen MicroMesh using a small amount of Cargille Immersion Oil. Data were collected on a Bruker three-circle platform diffractometer equipped with a PHOTON II CPAD detector. The crystals were irradiated using a 1 μs microfocus CuK_α_ source (λ = 1.54178) with Montel optics. Data were collected at room temperature (20 °C).

Data collection was performed and the unit cell was initially refined using the software *APEX3* (v2015.52, Madison, WI, USA). Data reduction was performed using software *SAINT* (v8.34A, Madison, WI, USA) and *XPREP* (v2014/2, Madison, WI, USA). Corrections were applied for Lorentz, polarization, and absorption effects using the software *SADABS* (v2014/2, Madison, WI, USA). The structure was solved and refined with the aid of the program SHELXL-2014/7 (Madison, WI, USA). The full-matrix least-squares refinement on F^2^ included atomic coordinates and anisotropic thermal parameters for all non-H atoms. Hydrogen atoms were located from the difference electron-density maps and added using a riding model.

Crystal data and atomic coordinates can be found in the Supplementary Materials.

## 4. Conclusions

Enantiopure stereoisomers of *N*-substituted ortho-C oxide-bridged phenylmorphans, were synthesized. (3*R*,6a*S*,11a*S*)-10-Methoxy-1,3,4,5,6,11a-hexahydro-2*H*-3,6a-methano-benzofuro[2,3-*c*]azocine **(+)-6** and its enantiomer, (3*S*, 6a*R*, 11a*R*)*-***(-)-6**, respectively, were the common intermediates, and the absolute configuration of the *R*)-(-)-*p*-methylmandelate salt of (3*S*, 6a*R*, 11a*R*)*-***(-)-6** was determined by single-crystal X-ray analysis. The enantiomeric secondary amines were reacted with *N*-(2-phenylcyclopropyl)methyl derivatives, the 2-(*E*)-cinnamyl bromide, (Z)-3-phenylacrylic acid. These products led to all of the desired N-derivatives of the ortho-C oxide-bridged phenylmorphans. Of these 14 compounds, three were found to have MOR binding affinity <50 nM. In comparison with the MOR affinity of the N-phenethyl ortho-C oxide-bridged phenylmorphan **(-)-1**, the *N*-propyl isomer **(-)-**25 had 20-fold less affinity at MOR. Both were partial agonists with little efficacy in the cAMP assay. One compound, **(+)17**, the (3S,6aR,11aR)-2-(((1S,2S)-2-phenylcyclopropyl)methyl)-1,3,4,5,6,11a-hexahydro-2H-3,6a-methanobenzofuro[2,3-c]azocin-10-ol isomer was a little more potent than naltrexone at MOR as an antagonist. Pharmacokinetic analysis, however, indicated that it had inhibitory effects on P450 isozymes that may cause potential drug–drug interactions. Further structural modification of **(+)-17** might eliminate those interactions.

## Figures and Tables

**Table 1:** Opioid receptor binding data for the enantiomeric ortho-C-phenylmorphans.

Receptor Binding (*K*i ± SEM, nM) ^a^				
	Structure	MOR Binding	DOR Binding	KOR Binding
(-) FL 3-134 **(-)-1**	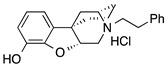	1.11 ± 0.15	34.8 ± 4.4	13.0 ± 1.2
(+)-FL-3-137 **(+)-16**	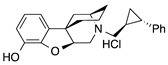	NT	NT	NT
(-) FL 3-147 **(-)-16**	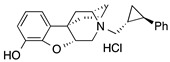	109 ± 5.5	NT	NT
(+) FL 3-138 **(+)-17**	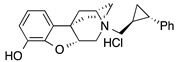	11.9 ± 1.6	49.4 ± 5.1	100 ± 10
(-)-FL-3-146 **(-)-17**	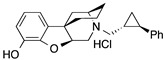	NT	NT	NT
(+)-FL-3-162 **(+)-18**	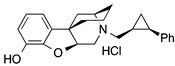	NT	NT	NT
(-) FL 3-169 **(-)-18**	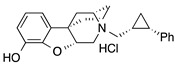	41.8 ± 4.4	89.0 ± 13.4	109 ± 16
(+) FL 3-163 **(+)-19**	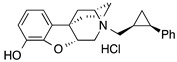	52.4 ± 5.1	NT	67.3 ± 7.9
(-)-FL-3-166 **(-)-19**	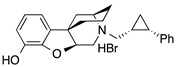	NT	NT	NT
(+)-FL-3-174 **(+)-21**	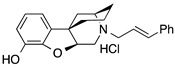	NT	NT	NT
(-)-FL-3-172 **(-)-21**	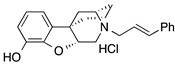	NT	NT	NT
(+)-FL 3-178 **(+)-24**	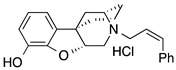	89.3 ± 5.3	NT	NT
(-)-FL-3-153 **(-)-24**	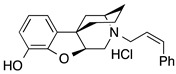	NT	NT	NT
(+)-FL-3-181 **(+)-25**	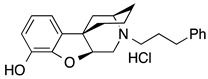	NT	NT	NT
(-)-FL-3-182 **(-)-25**	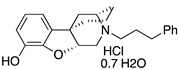	21.8 ± 1.3	69.9 ± 9.5	135 ± 10

^a^ Binding assays were typically conducted in at least three independent experiments, each performed with triplicate observations using whole rat brains excluding cerebellum; *K*i ± SEM (nM); NT = not tested—inactive (<50% activity at 100 nM concentration in exploratory binding assays (displaced less than half of radioligand). Compounds with low binding affinity (>50 nM) were not further examined in functional assays).

**Table 2:** Opioid receptor activity measured in the forskolin-induced cAMP accumulation assay ^a^.

		MOR		DOR		KOR	
		Agonist	Antagonist ^b^	Agonist	Antagonist ^c^	Agonist	Antagonist ^d^
Name	Structure	EC_50_ ± SEM (nM) (%E_max_ ± SEM)	IC_50_ ± SEM (nM) (%I_max_ ± SEM)	EC_50_ ± SEM (nM) (%E_max_ ± SEM)	IC_50_ ± SEM (nM) (%I_max_ ± SEM)	EC_50_ ± SEM (nM) (%E_max_ ± SEM)	IC_50_ ± SEM (nM) (%I_max_ ± SEM)
(-)-FL 3-134 **(-)-1**	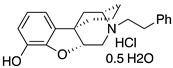	2.66 ± 0.47 (35.0 ± 5.8%)	4.67 ± 1.03 (66.9 ± 6.7%)	>10,000	408.83 ± 138.24 (86.5 ± 6.4%)	>10,000	102.03 ± 65.82 (109.3 ± 8.1%)
(+)-FL 3-138 **(+)-17**	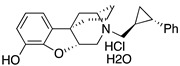	>10,000	6.92 ± 1.47 (153.4 ± 19.2%)	>10,000	487.28 ± 157.43 (160.9 ± 4.0%)	>10,000	206.20 ± 63.16 (106.1 ± 5.5%)
(-)-FL-3-182 **(-)-25**	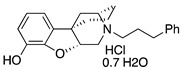	21.62 ± 12.41 (22.5 ± 2.1%)	159.70 ± 65.87 (92.4 ± 0.3%)	>10,000	>10,000	>10,000	1275.29 ± 340.44 (105.9 ± 4.6%)
	Naltrexone	2.14 ± 1.2 (29.6 ± 6.4%)	10.8 ± 1.0 (103.5 ± 0.6%)	>10,000	295.1 ± 47.5 (99.4 ± 1.1%)	0.64 ± 0.32 (56.5 ± 7.2%)	5.53 ± 1.02 (41.3 ± 6.8%)
	Morphine	6.28 ± 0.43 (102.1 ± 0.2%)					

^a^ Inhibition of forskolin-induced cAMP accumulation; cAMP Hunter^TM^ Chinese hamster ovary cells (CHO-K1) that express human μ-opioid receptor (OPRM1), human κ-opioid receptor (OPRK1), and human δ-opioid receptor (OPRD1) were used for the forskolin-induced cAMP accumulation assay to determine potency and efficacy of the compounds following the previously established methods [17]. To determine % efficacy in forskolin-induced cAMP assays, data were blank subtracted with the vehicle control, followed by normalization to the forskolin control. Data were then analyzed in GraphPad Prism 8 (GraphPad, LaJolla, CA, USA) using nonlinear regression; values are expressed as the mean ± SEM of at least three independent experiments; ^b^ MOR antagonist potency (IC50) determined versus EC90 of fentanyl; degree of antagonism (Imax) normalized to naltrexone. ^c^ DOR antagonist potency (IC50) determined versus EC50 of SNC80; degree of antagonism (Imax) normalized to naltrexone. ^d^ KOR antagonist potency (IC50) determined versus EC90 of U50488H; degree of antagonism (Imax) normalized to nor-BNI.

**Table 3:** Effect of test compounds at 10 µM on P450 inhibition.

Compound	1A2	2C9	2C19	2D6	3A
	%Inhibition	%Inhibition	%Inhibition	%Inhibition	%Inhibition
**(+)-17** (+)-FL-3-138	6.8	36.6	46.6	66.8	24.7
Naltrexone	1.0	0.0	0.0	36.7	0.0
Naloxone	2.0	0.0	0.0	17.9	1.4
α-Naphthoflavone	88.3				
Sulfaphenazole		85.2			
(+)-*N*-3-benzylnirvanol			83.4		
Quinidine				94.2	
Ketoconazole					98.8

**Table 4:** Microsomal metabolic stability study of test compounds with human and mouse liver microsomes.

**Compound**	**HLM 0.5**					
	**R^2 a^**	**T_1/2_ ^b^** **(min)**	**CL_int(mic)_** **(μL/min/mg) ^c^**	**CL_int(liver)_** **(mL/min/kg) ^d^**	**Remaining** **(T = 60 min)**	**Remaining** **(NCF = 60 min) ^e^**
**(+)-17** (+)-FL-3-138	0.9976	20.1	68.9	62.0	13.0%	98.5%
Naltrexone	0.0812	>145	<9.6	<8.6	97.1%	99.8%
Naloxone	0.6305	>145	<9.6	<8.6	78.4%	107.6%
Testosterone	0.8887	12.9	107.1	96.4	3.9%	86.7%
Diclofenac	0.9986	7.7	180.4	162.4	0.4%	95.1%
Propafenone	0.9447	6.0	230.4	207.4	0.1%	95.8%
**Compound**	**MLM 0.5**					
	**R^2 a^**	**T_1/2_ ^b^** **(min)**	**CL_int(mic)_** **(μL/min/mg) ^c^**	**CL_int(liver)_** **(mL/min/kg) ^d^**	**Remaining** **(T = 60 min)**	**Remaining** **(NCF = 60 min) ^e^**
**(+)-17** (+)-FL-3-138	0.9456	6.9	202.2	800.8	0.2%	94.4%
Naltrexone	0.9970	57.4	24.1	95.5	48.1%	84.3%
Naloxone	0.9939	14.4	96.4	381.6	5.2%	112.5%
Testosterone	0.9989	3.2	435.2	1723.4	0.0%	97.1%
Diclofenac	0.9801	39.2	35.4	140.0	33.5%	99.4%
Propafenone	1.0000	1.8	788.6	3122.8	0.0%	105.6%

^a^ R^2^: correlation coefficient of the linear regression for the determination of kinetic constant. ^b^ T_1/2_: half-life. ^c^ CL_int(mic)_: intrinsic clearance, CL_int(mic)_ = 0.693/T_1/2_/mg microsome protein per mL. ^d^ CL_int(liver)_ = CL_int(mic)_ × mg microsomal protein/g liver weight× *g* liver weight/kg body weight. ^e^ NCF: no co-factor. No NADPH is added to NCF samples (replaced by buffer) during the 60-min incubation. If the NCF remaining is less than 60%, then possibly non-NADPH-dependent metabolism occurs.

## Data Availability

The publicly available Cambridge Structural Database was used to store the crystal structure for compound **(-)-6**.

## Supplementary Materials

The following are available at https://www.mdpi.com/article/10.3390/molecules27248808/s1, ^1^H and ^13^C-NMR spectra of novel compounds, Tables S1–S7: X-ray crystal data for **(-)-6**.

## Abbreviations

MOR: mu-opioid receptor; DOR, delta-opioid receptor; KOR, kappa-opioid receptor; cAMP, cyclic adenosine monophosphate; DAMGO, [D-Ala2; N-Me-Phe4; Gly5-ol]-enkephalin.

## References

[1] Machelska H., Celik M.Ö. (2018). Advances in Achieving Opioid Analgesia without Side Effects. Front. Pharmacol..

[2] Günther T., Dasgupta P., Mann A., Miess E., Kliewer A., Fritzwanker S., Steinborn R., Schulz S. (2018). Targeting multiple opioid receptors—Improved analgesics with reduced side effects?. Br. J. Pharmacol..

[3] Azevedo Neto J., Costanzini A., De Giorgio R., Lambert D.G., Ruzza C., Calò G. (2020). Biased versus Partial Agonism in the Search for Safer Opioid Analgesics. Molecules.

[4] Stahl E.L., Bohn L.M. (2022). Low Intrinsic Efficacy Alone Cannot Explain the Improved Side Effect Profiles of New Opioid Agonists. Biochemistry.

[5] Burke T.R., Jacobson A.E., Rice K.C., Weissman B.A., Silverton J.V., Harris L.S. (1984). Probes for narcotic receptor mediated phenomena 3. Oxide bridged 5-phenylmorphans. Problems of Drug Dependence 1983.

[6] Yamada K., Flippen-Anderson J.L., Jacobson A.E., Rice K.C. (2002). Probes for Narcotic Receptor Mediated Phenomena; 29: Synthesis of *rac*-(4*R*,6a*R*,11b*R*)-3-Methyl 2,3,4,5,6,6a hexahydro-1*H* 4,11b-methanobenzofuro[3,2-*d*]azocin-10-ol, the *para*-a Oxide-bridged Phenylmorphan Isomer, and a New Route to *rac*-(4*R*,6a*R*,11b*R*)-3-Methyl-2,3,4,5,6,6a-hexahydro-1*H*-4,11b-methanobenzofuro[3,2-*d*]azocin-8-ol, the *ortho*-a oxide-bridged phenylmorphan isomer. Synth.-Stuttg..

[7] Kurimura M., Liu H., Sulima A., Przybyl A.K., Ohshima E., Kodato S., Deschamps J.R., Dersch C., Rothman R.B., Lee Y.S. (2008). Probes for narcotic receptor mediated phenomena. 37. Synthesis and opioid binding affinity of the final pair of oxide-bridged phenylmorphans, the *ortho*- and *para*-b isomers and their *N*-phenethyl analogues, and the synthesis of the *N*-phenethyl analogues of the *ortho*- and *para*-d isomers. J. Med. Chem..

[8] Tadic D., Linders J.T.M., Flippen-Anderson J.L., Jacobson A.E., Rice K.C. (2003). Probes for narcotic receptor mediated phenomena. Part 31: Synthesis of *rac*-(3*R*,6a*S*,11a*S*)-2-methyl-1,3,4,5,6,11a-hexahydro-2*H*-3,6a-methanobenzofuro[2,3-*c*]azocine-10-ol, and azocine-8-ol, the *ortho*-c and the *para*-c oxide-bridged phenylmorphan isomers. Tetrahedron.

[9] Burke T.R., Jacobson A.E., Rice K.C., Weissman B.A., Huang H.C., Silverton J.V. (1986). Probes for narcotic receptor mediated phenomena. 9. Synthesis of (±)-(3α,6aα,11aβ)-1,3,4,5,6,11a-hexahydro-2-methyl-2*H*-3,6a-methanobenzofuro[2,3-*c*]azocin-10-ol, an oxide-bridged 5-(*m*-hydroxyphenyl)morphan. J. Med. Chem..

[10] Linders J.T.M., Mirsadeghi S., Flippen-Anderson J.L., George C., Jacobson A.E., Rice K.C. (2003). Probes for narcotic receptor mediated phenomena. Part 30. Synthesis of *rac*-(3*R*,6a*S*,11a*R*)-2-methyl-1,3,4,5,6,11a-hexahydro-2*H*-3,6a-methanobenzofuro[2,3-*c*]azocin-8-ol, an epoxy isomer of 5-phenylmorphan. Helv. Chim. Acta.

[11] Zezula J., Singer L.B., Przybyl A.K., Hashimoto A., Dersch C.M., Rothman R.B., Deschamps J., Lee Y.S., Jacobson A.E., Rice K.C. (2008). Synthesis and pharmacological effects of the enantiomers of the *N*-phenethyl analogues of the *ortho* and *para* e- and f-oxide-bridged phenylmorphans. Org. Biomol. Chem..

[12] Hashimoto A., Przybyl A.K., Linders J.T.M., Kodato S., Tian X.R., Deschamps J.R., George C., Flippen-Anderson J.L., Jacobson A.E., Rice K.C. (2004). Probes for narcotic receptor-mediated phenomena. 33. Construction of a strained trans-5,6-ring system by displacement of a nitro-activated aromatic fluorine. Synthesis of the penultimate oxide-bridged phenylmorphans. J. Org. Chem..

[13] Kodato S., Linders J.T.M., Gu X.-H., Yamada K., Flippen-Anderson J.L., Deschamps J.R., Jacobson A.E., Rice K.C. (2004). Synthesis of *rac*-(1*R*,4a*R*,9a*R*)-2-methyl-1,3,4,9a-tetrahydro-2*H*-1,4a-propanobenzofuro[2.3-*c*]pyridin-6-ol. An Unusual Double Rearrangement Leading to the *ortho*- and *para*–f Oxide-Bridged Phenylmorphan Isomers. Org. Biomolec. Chem..

[14] Kim J.H., Deschamps J.R., Rothman R.B., Dersch C.M., Folk J.E., Cheng K., Jacobson A.E., Rice K.C. (2011). Probes for narcotic receptor mediated phenomena. Part 42: Synthesis and in vitro pharmacological characterization of the N-methyl and N-phenethyl analogues of the racemic ortho-c and para-c oxide-bridged phenylmorphans. Bioorg. Med. Chem..

[15] Cheng K., Kim I.J., Lee M.J., Adah S.A., Raymond T.J., Bilsky E.J., Aceto M.D., May E.L., Harris L.S., Coop A. (2007). Opioid Ligands with Mixed Properties from Substituted Enantiomeric N-Phenethyl-5-phenylmorphans. Synthesis of a μ-Agonist δ-Antagonist and δ-Inverse Agonists. Org. Biomolec. Chem..

[16] Cheng K., Lee Y.S., Rothman R.B., Dersch C.M., Bittman R.W., Jacobson A.E., Rice K.C. (2011). Probes for Narcotic Receptor Mediated Phenomena. 41. Unusual Inverse μ-Agonists and Potent μ-Opioid Antagonists by Modification of the N-Substituent in Enantiomeric 5-(3-Hydroxyphenyl)morphans. J. Med. Chem..

[17] Hedrick S.L., Luo D., Kaska S., Niloy K.K., Jackson K., Sarma R., Horn J., Baynard C., Leggas M., Eduardo R. (2021). Design, synthesis, and preliminary evaluation of a potential synthetic opioid rescue agent. J. Biomed. Sci..

[18] Crowley R.S., Riley A.P., Alder A.F., Anderson R.J., Luo D., Kaska S., Maynez P., Kivell B.M., Prisinzano T.E. (2020). Synthetic Studies of Neoclerodane Diterpenes from Salvia divinorum: Design, Synthesis, and Evaluation of Analogues with Improved Potency and G-protein Activation Bias at the μ-Opioid Receptor. ACS Chemical. Neurosci..

[19] Riley A.P., Groer C.E., Young D., Ewald A.W., Kivell B.M., Prisinzano T.E. (2014). Synthesis and κ-Opioid Receptor Activity of Furan-Substituted Salvinorin A Analogues. J. Med. Chem..

