# Supplementary material for: Synthesis and Pharmacological Evaluation of Enantiopure N-Substituted Ortho-c Oxide-Bridged 5-Phenylmorphans
Source: Molecules. 2022 Dec 12;27(24):8808. doi: 10.3390/molecules27248808 (PMC9785231; doi:10.3390/molecules27248808)
Supplement: Supplementary file 1 [file molecules-27-08808-s001.zip › molecules-2019928-supplementary.pdf]

SUPPLEMENTARY MATERIALS

Fuying Li[1], Theresa A. Kopajtic[2], Jonathan L. Katz[2], Dan Luo[3], Thomas E. Prisinzano[3], Gregory H. Imler[4], Jeffrey R. Deschamps[4], Arthur E. Jacobson[1,*], Kenner C. Rice[1,*]

[1]Drug Design and Synthesis Section, Molecular Targets and Medications Discovery Branch, National Institute on Drug Abuse and the National Institute on Alcohol Abuse and Alcoholism, National Institutes of Health, Department of Health and Human Services, 5625 Fishers, Rockville, MD 20852, USA

[2]Psychobiology Section, Molecular Neuropsychiatry Research Branch, Intramural Research Program, National Institute on Drug Abuse, National Institutes of Health, Baltimore, MD 21224, USA

[3]Department of Pharmaceutical Sciences, College of Pharmacy, University of Kentucky, 789 S. Limestone Street, Lexington, Kentucky 40536, USA

[4]Center for Biomolecular Science and Engineering, Naval Research Laboratory, Washington DC 20375, USA

*Correspondence: kennerr@nida.nih.gov; Tel.: 301-451-4799, arthurj@nida.nih.gov; Tel.: 301-451-5028

Table of Contents

1) ^{1}H (top of page) & ^{13}C NMR (bottom of page) 3 - 17

2) Table S1 – S7. Crystal data, atomic coordinates, etc, for compound **(-)-6** 18 - 26

Compound **5**

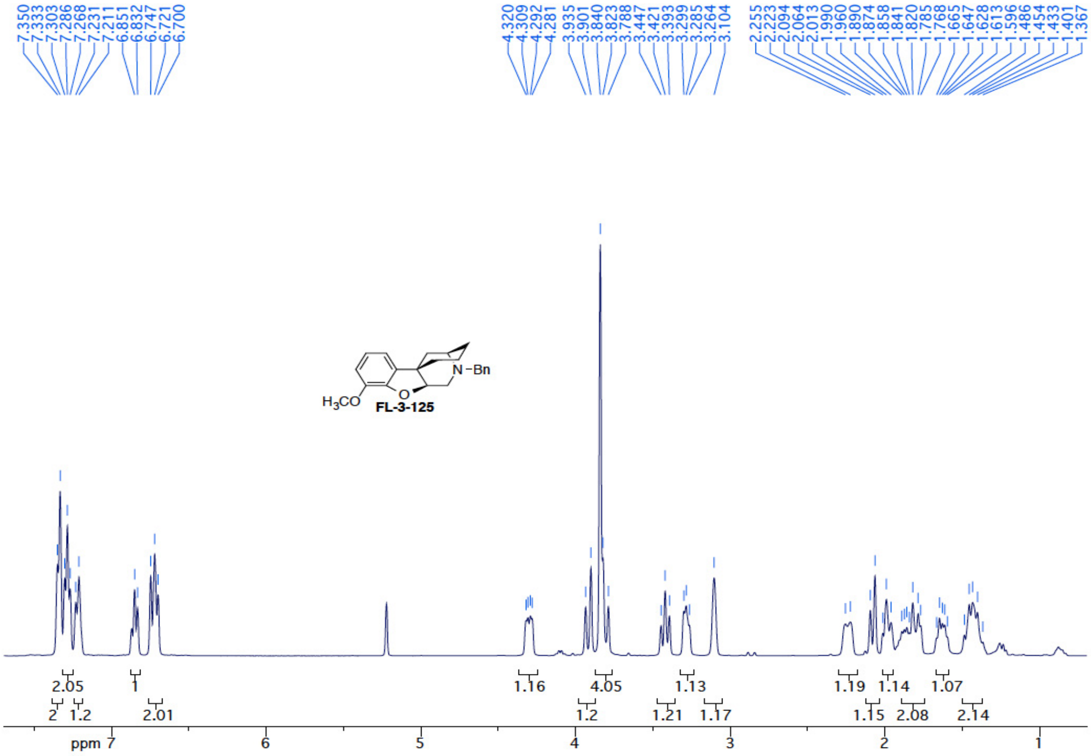
7.350
7.333
7.303
7.286
7.268
7.231
7.211
6.851
6.832
6.747
6.721
6.700
4.320
4.309
4.292
4.281
3.935
3.901
3.840
3.823
3.788
3.447
3.421
3.393
3.299
3.285
3.264
3.104
2.255
2.223
2.094
2.064
2.013
1.990
1.960
1.890
1.874
1.858
1.841
1.820
1.785
1.768
1.665
1.647
1.628
1.613
1.596
1.486
1.454
1.433
1.401
1.367
N-Bn
H3CO
O
FL-3-125
2.05
1
1.16
4.05
1.13
1.19
1.14
1.07
2
1.2
2.01
1.2
1.21
1.17
1.15
2.08
2.14
ppm 7
6
5
4
3
2
1

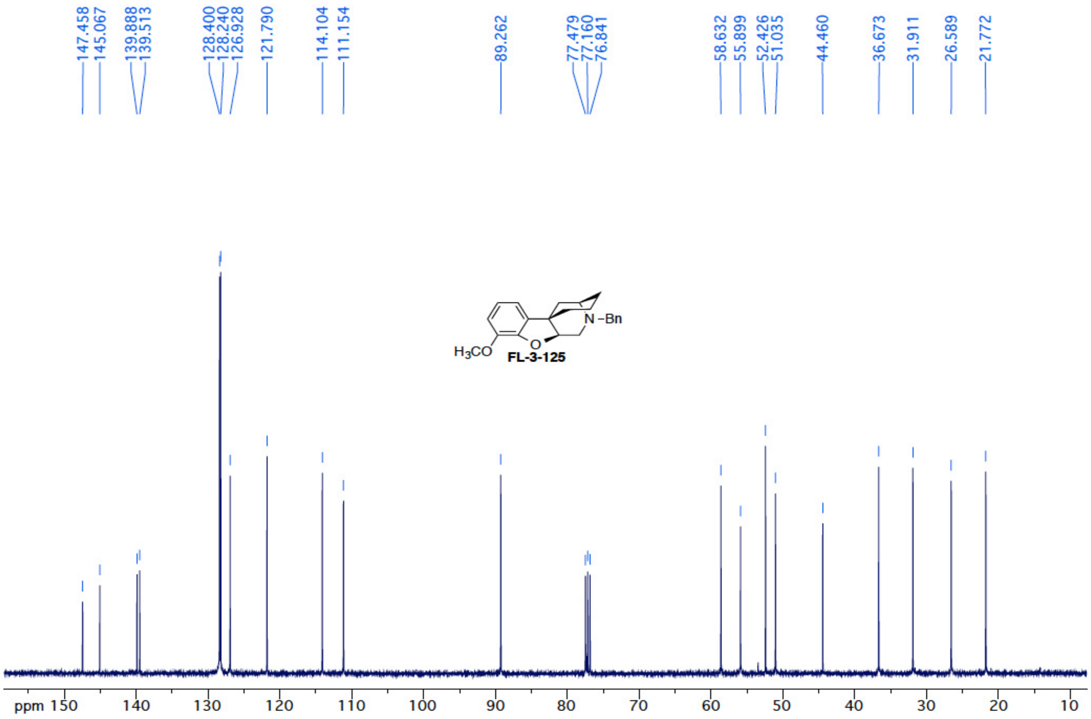
147.458
145.067
139.888
139.513
128.400
128.240
126.928
121.790
114.104
111.154
89.262
77.479
77.160
76.841
58.632
55.899
52.426
51.035
44.460
36.673
31.911
26.589
21.772
N-Bn
H3CO
O
FL-3-125
ppm 150
140
130
120
110
100
90
80
70
60
50
40
30
20
10

Compound **6**

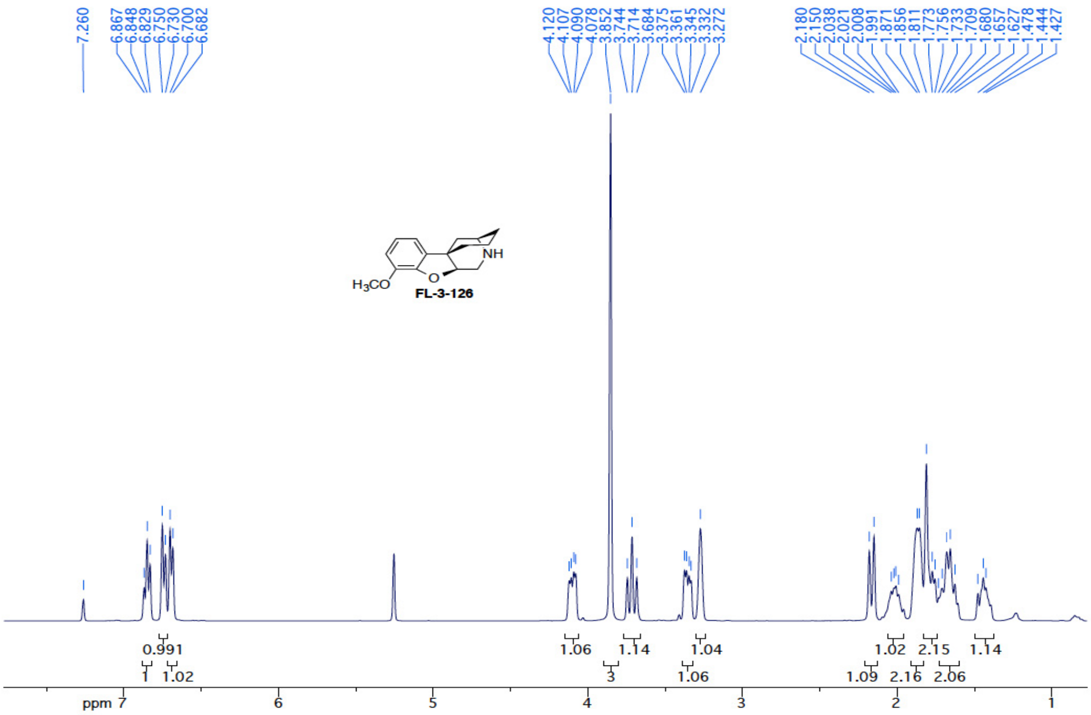

7.260
6.867
6.848
6.829
6.750
6.730
6.700
6.682
4.120
4.107
4.090
4.078
3.852
3.744
3.714
3.684
3.375
3.361
3.345
3.332
3.272
2.180
2.150
2.038
2.021
2.008
1.991
1.871
1.856
1.811
1.773
1.756
1.733
1.709
1.680
1.657
1.627
1.478
1.444
1.427
H3CO
NH
O
FL-3-126
0.991
1
1.02
1.06
3
1.14
1.06
1.04
1.09
1.02
2.16
2.15
2.06
1.14
ppm 7
6
5
4
3
2
1

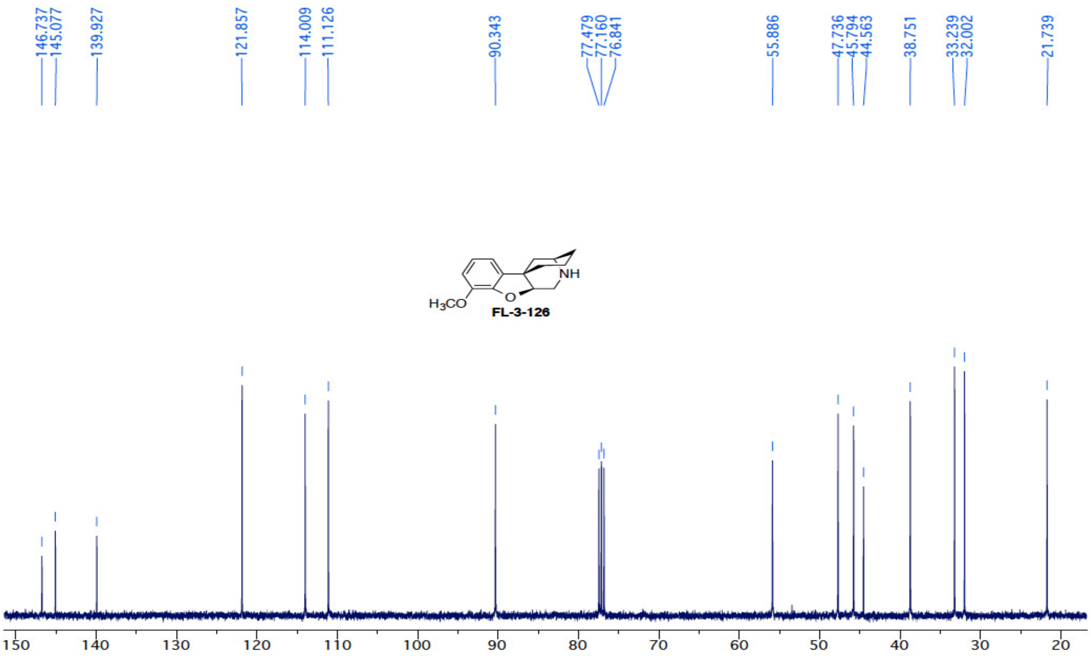

146.737
145.077
139.927
121.857
114.009
111.126
90.343
77.479
77.160
76.841
55.886
47.736
45.794
44.563
38.751
33.239
32.002
21.739
H3CO
NH
O
FL-3-126
150
140
130
120
110
100
90
80
70
60
50
40
30
20

Compound **7**

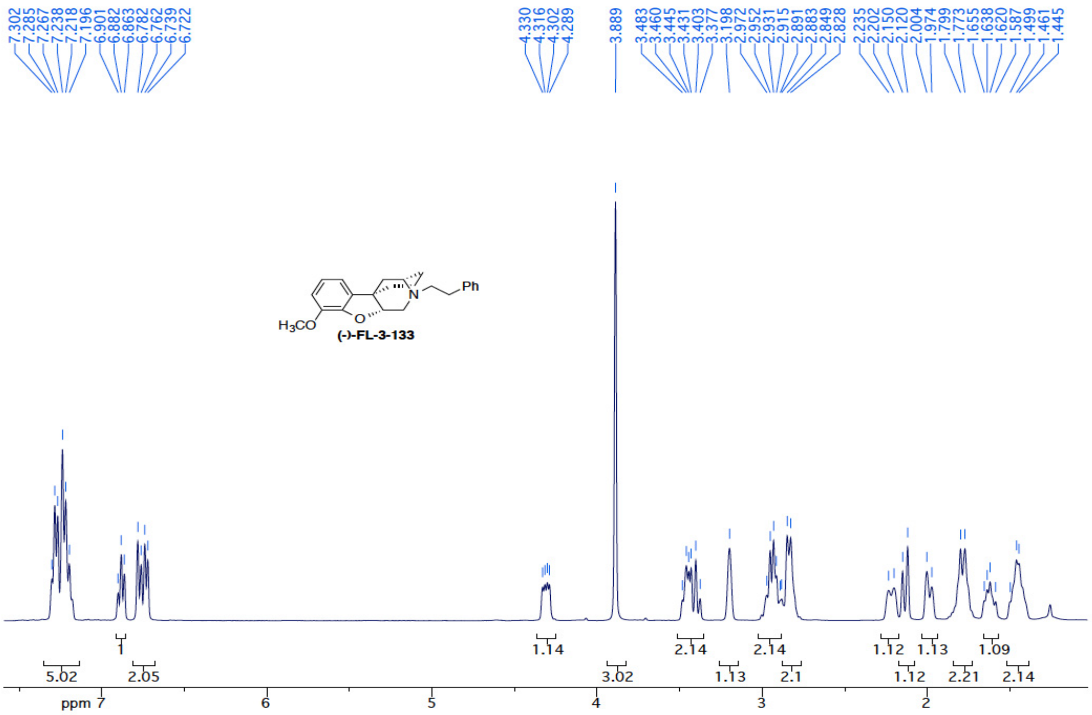

7.302
7.285
7.267
7.238
7.218
7.196
6.901
6.882
6.863
6.782
6.762
6.739
6.722
4.330
4.316
4.302
4.289
3.889
3.483
3.460
3.445
3.431
3.403
3.377
3.198
2.972
2.952
2.931
2.915
2.891
2.883
2.849
2.828
2.235
2.202
2.150
2.120
2.004
1.974
1.799
1.773
1.655
1.638
1.620
1.587
1.499
1.461
1.445
H3CO
O
N
Ph
(-)-FL-3-133
5.02
1
2.05
1.14
3.02
2.14
1.13
2.14
2.1
1.12
1.12
1.13
2.21
1.09
2.14
ppm
7
6
5
4
3
2

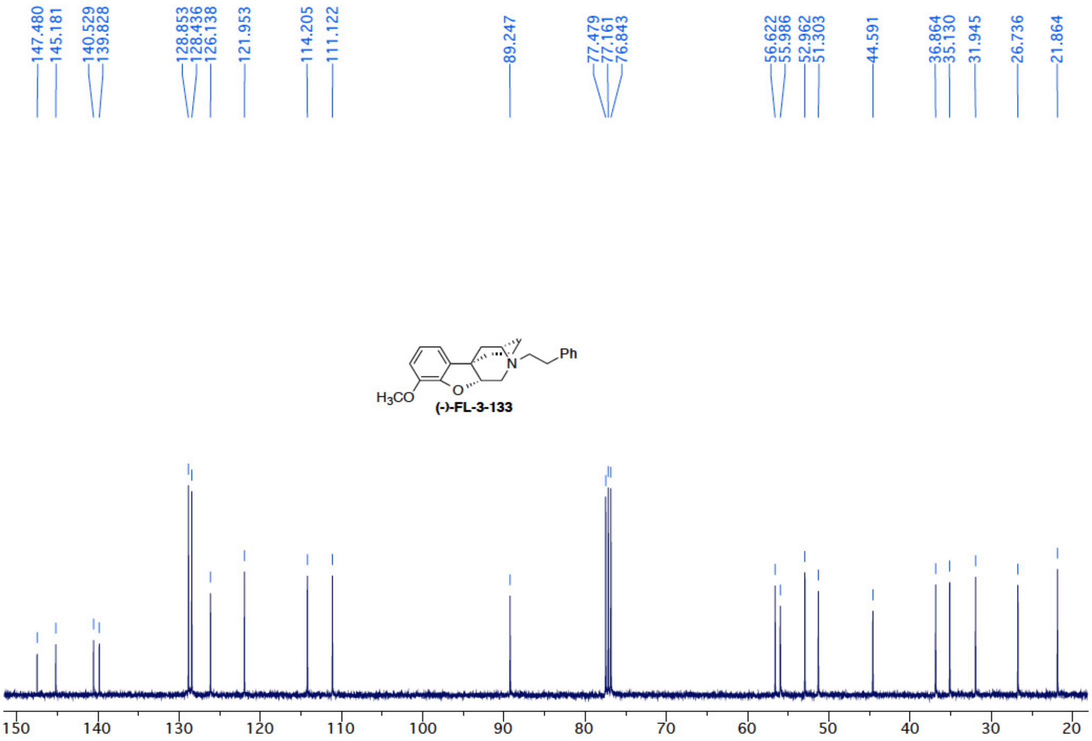

147.480
145.181
140.529
139.828
128.853
128.436
126.138
121.953
114.205
111.122
89.247
77.479
77.161
76.843
56.622
55.986
52.962
51.303
44.591
36.864
35.130
31.945
26.736
21.864
H3CO
O
N
Ph
(-)-FL-3-133
150
140
130
120
110
100
90
80
70
60
50
40
30
20

Compound (+)-8

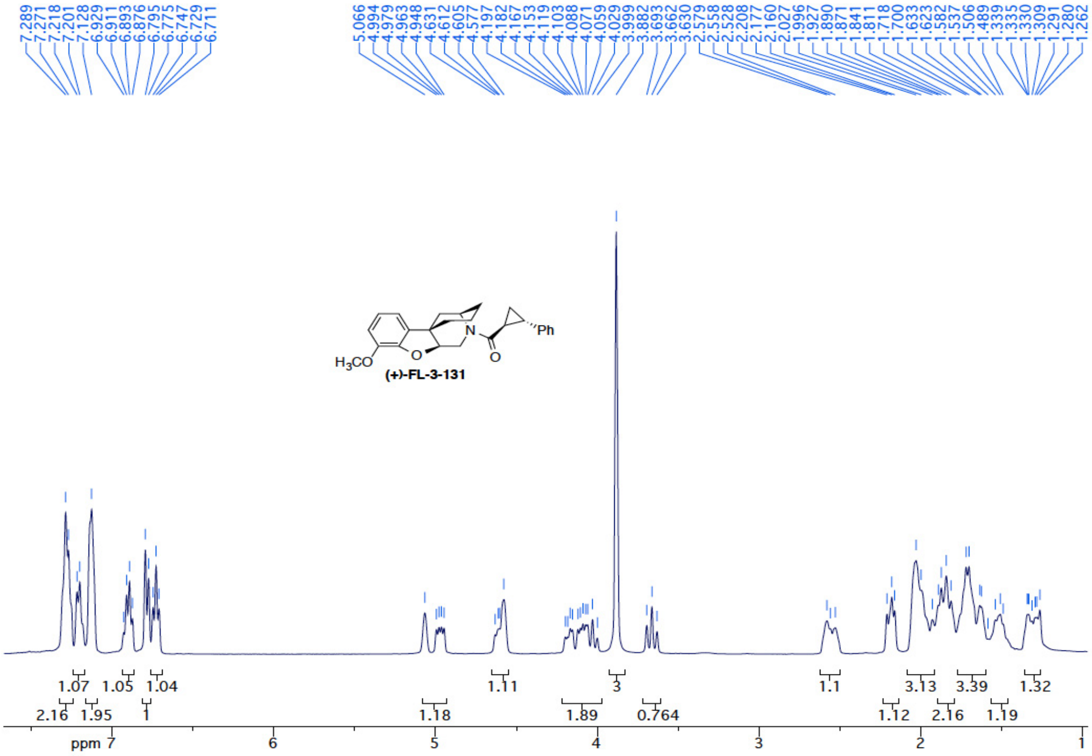
7.289 7.271 7.218 7.201 7.128 6.929 6.911 6.893 6.876 6.795 6.775 6.747 6.729 6.711
5.066 4.994 4.979 4.963 4.948 4.631 4.612 4.605 4.577 4.197 4.182 4.167 4.153 4.119 4.103 4.088 4.071 4.059 4.029 3.999 3.882 3.693 3.662 3.630 2.579 2.558 2.528 2.208 2.177 2.160 2.027 1.996 1.927 1.890 1.871 1.841 1.811 1.718 1.700 1.633 1.623 1.582 1.537 1.506 1.489 1.339 1.335 1.330 1.309 1.291 1.280 1.262
H3CO
N
O
Ph
(+)-FL-3-131
1.07 1.05 1.04 1.11 3 1.1 3.13 3.39 1.32
2.16 1.95 1 1.18 1.89 0.764 1.12 2.16 1.19
ppm 7 6 5 4 3 2 1

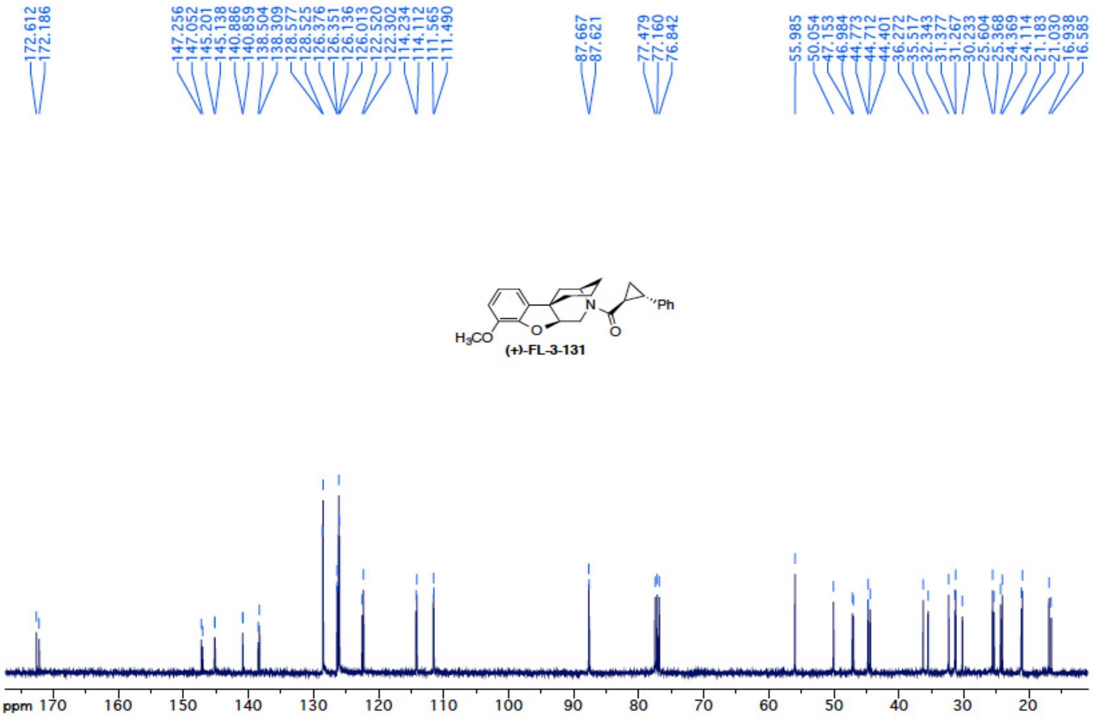
172.612 172.186
147.256 147.052 145.201 145.138 140.886 140.859 138.504 138.309 128.577 128.525 126.376 126.351 126.136 126.013 122.520 122.302 114.234 114.112 111.565 111.490
87.667 87.621
77.479 77.160 76.842
55.985 50.054 47.153 46.984 44.773 44.712 44.401 36.272 35.517 32.343 31.377 31.267 30.233 25.604 25.368 24.369 24.114 21.183 21.030 16.938 16.585
H3CO
N
O
Ph
(+)-FL-3-131
ppm 170 160 150 140 130 120 110 100 90 80 70 60 50 40 30 20

Compound (-)-**8**

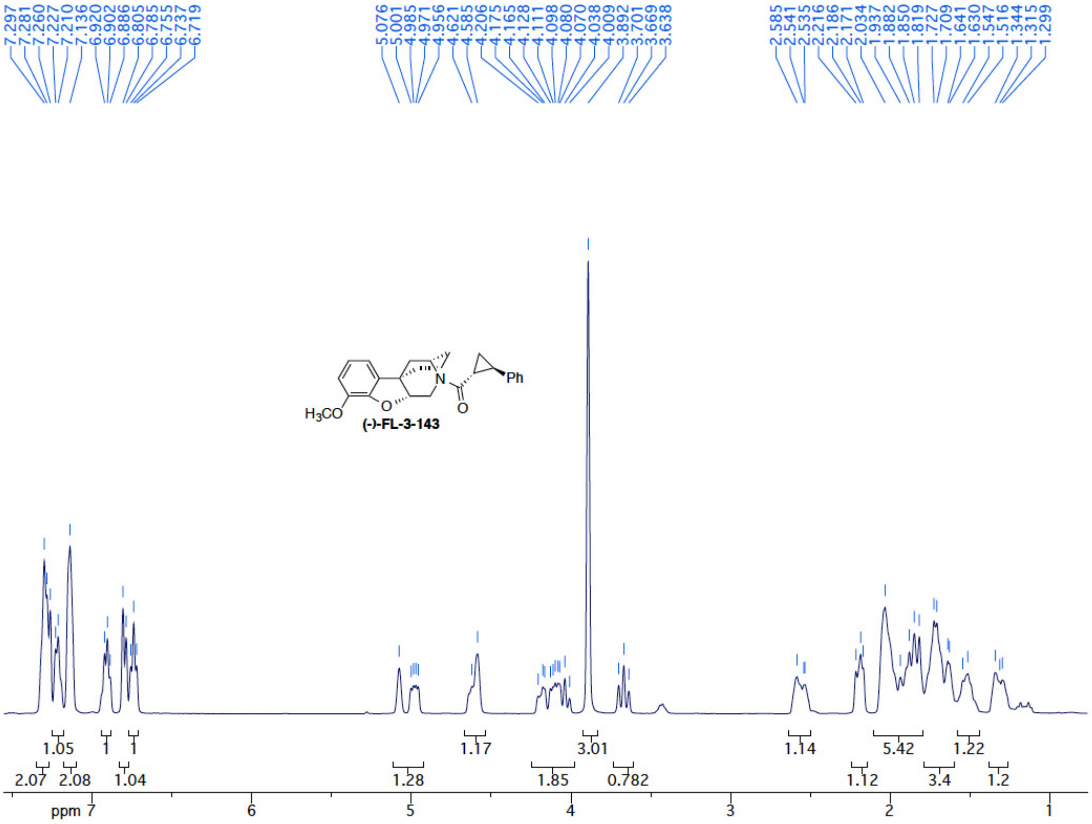
7.297
7.281
7.260
7.227
7.210
7.136
6.920
6.902
6.886
6.805
6.785
6.755
6.737
6.719
5.076
5.001
4.985
4.971
4.956
4.621
4.585
4.206
4.175
4.165
4.128
4.111
4.098
4.080
4.070
4.038
4.009
3.892
3.701
3.669
3.638
2.585
2.541
2.535
2.216
2.186
2.171
2.034
1.937
1.882
1.850
1.819
1.727
1.709
1.641
1.630
1.547
1.516
1.344
1.315
1.299
H3CO
O
N
O
Ph
(-)-FL-3-143
1.05
1
1
1.17
3.01
1.14
5.42
1.22
2.07
2.08
1.04
1.28
1.85
0.782
1.12
3.4
1.2
ppm 7
6
5
4
3
2
1

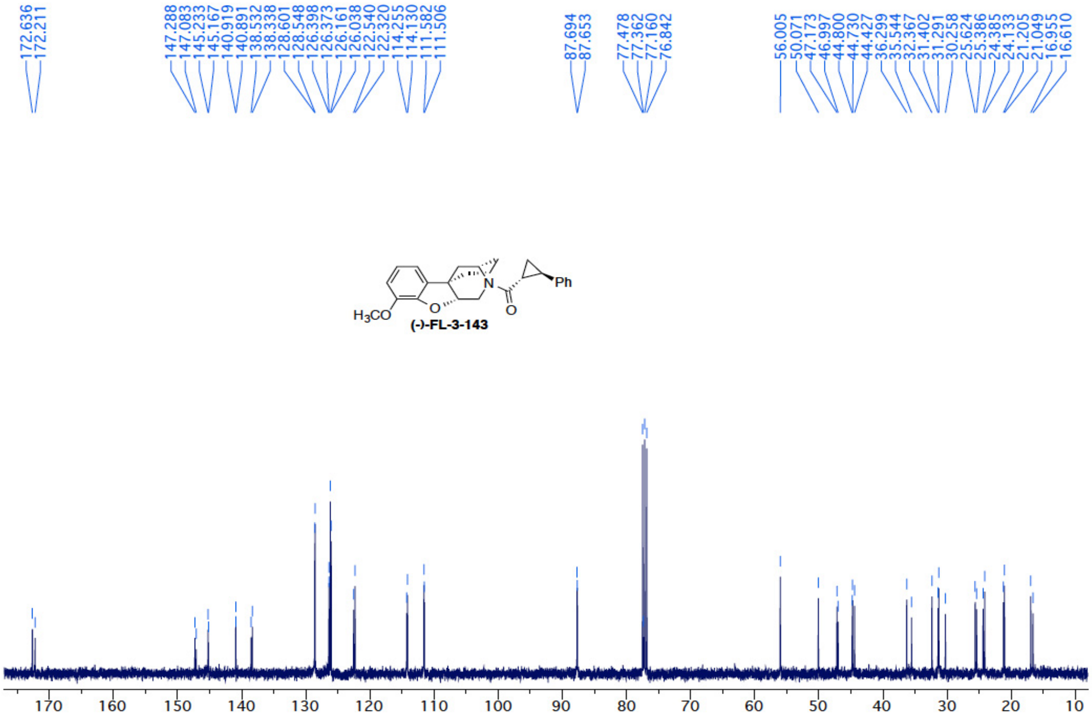
172.636
172.211
147.288
147.083
145.233
145.167
140.919
140.891
138.532
138.338
128.601
128.548
126.398
126.373
126.161
126.038
122.540
122.320
114.255
114.130
111.582
111.506
87.694
87.653
77.478
77.362
77.160
76.842
56.005
50.071
47.173
46.997
44.800
44.730
44.427
36.299
35.544
32.367
31.402
31.291
30.258
25.624
25.386
24.385
24.133
21.205
21.049
16.955
16.610
H3CO
O
N
O
Ph
(-)-FL-3-143
170
160
150
140
130
120
110
100
90
80
70
60
50
40
30
20
10

Compound (+)-**9**

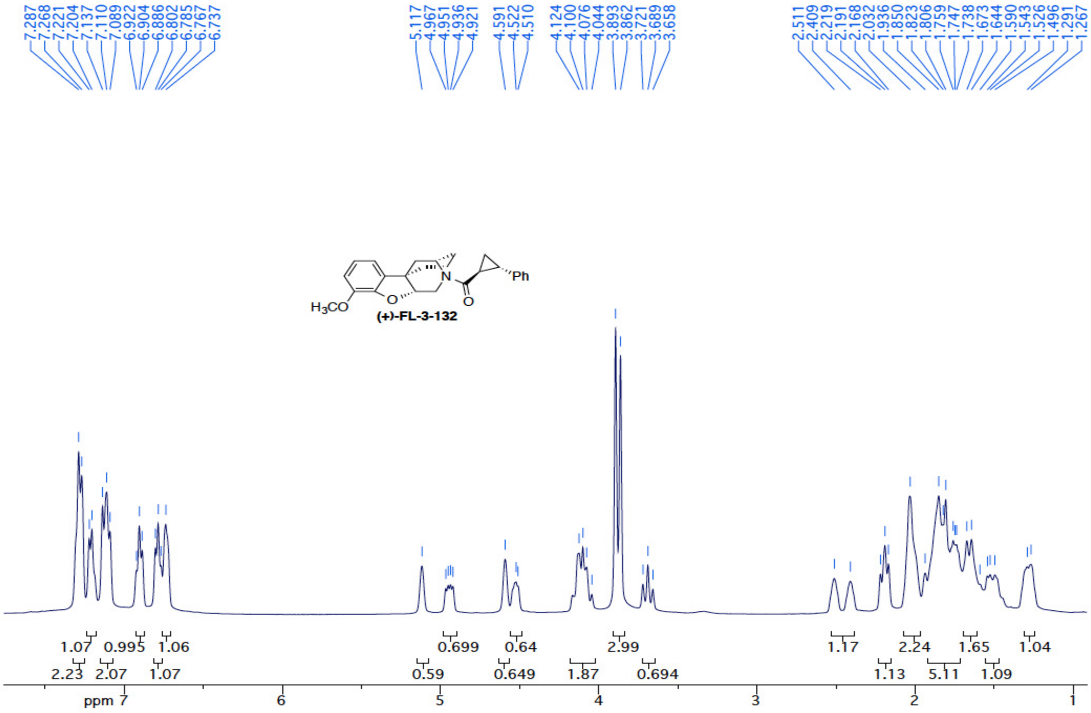
7.287
7.268
7.221
7.204
7.137
7.110
7.089
6.922
6.904
6.886
6.802
6.785
6.767
6.737
5.117
4.967
4.951
4.936
4.921
4.591
4.522
4.510
4.124
4.100
4.076
4.044
3.893
3.862
3.721
3.689
3.658
2.511
2.409
2.219
2.191
2.168
2.032
1.936
1.850
1.823
1.806
1.759
1.747
1.738
1.673
1.644
1.590
1.543
1.526
1.496
1.291
1.267
H3CO
O
N
O
Ph
(+)-FL-3-132
1.07
0.995
1.06
2.23
2.07
1.07
0.699
0.64
2.99
0.59
0.649
1.87
0.694
1.17
2.24
1.65
1.04
1.13
5.11
1.09
ppm 7
6
5
4
3
2
1

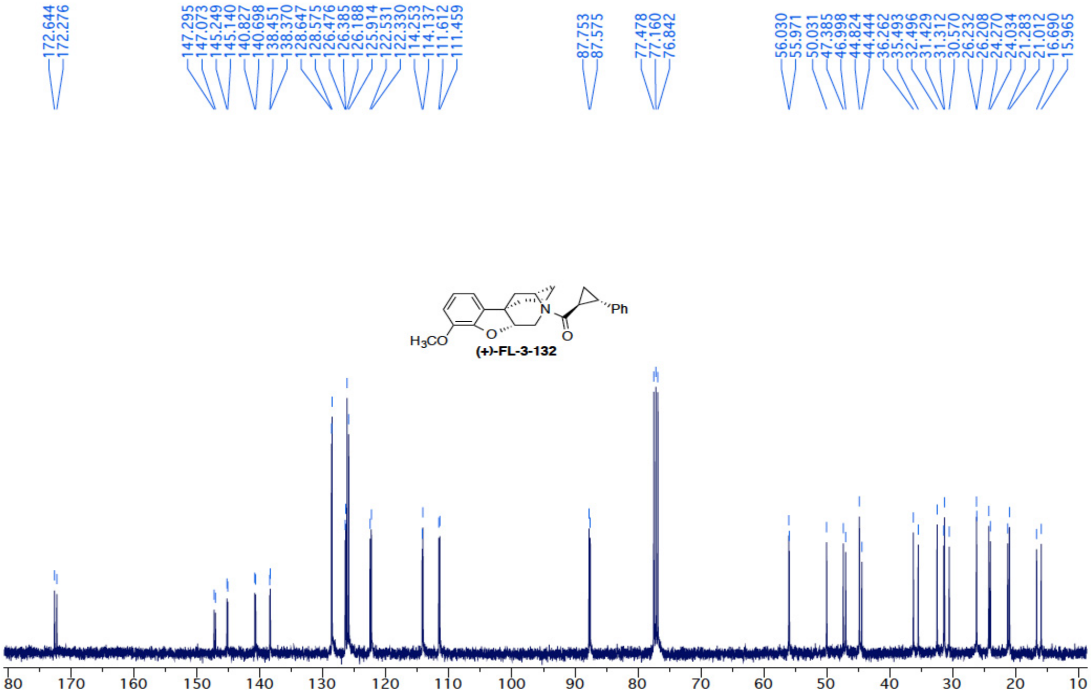
172.644
172.276
147.295
147.073
145.249
145.140
140.827
140.698
138.451
138.370
128.647
128.575
126.476
126.385
126.188
125.914
122.531
122.330
114.253
114.137
111.612
111.459
87.753
87.575
77.478
77.160
76.842
56.030
55.971
50.031
47.385
46.998
44.824
44.444
36.262
35.493
32.496
31.429
31.312
30.570
26.232
26.208
24.270
24.034
21.283
21.012
16.690
15.965
H3CO
O
N
O
Ph
(+)-FL-3-132
80
170
160
150
140
130
120
110
100
90
80
70
60
50
40
30
20
10

Compound (-)-**9**

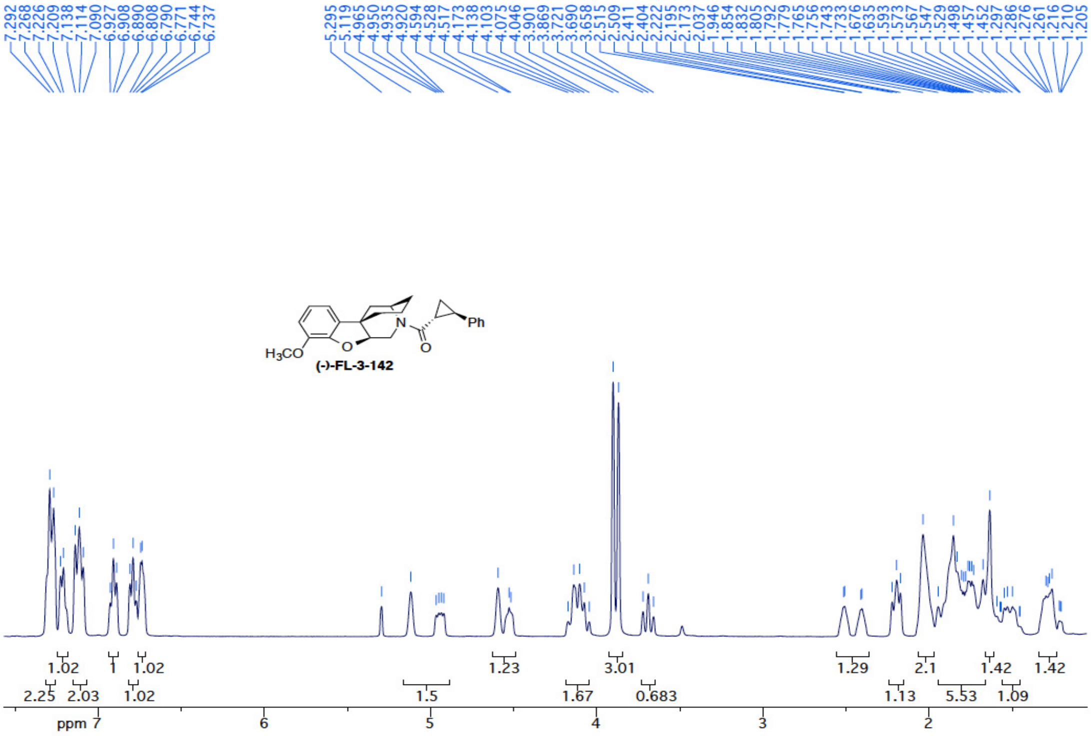

7.292
7.268
7.226
7.209
7.138
7.114
7.090
6.927
6.908
6.890
6.808
6.790
6.771
6.744
6.737
5.295
5.119
4.965
4.950
4.935
4.920
4.594
4.528
4.517
4.173
4.138
4.103
4.075
4.046
3.901
3.869
3.721
3.690
3.658
2.515
2.509
2.411
2.404
2.222
2.195
2.173
2.037
1.946
1.854
1.832
1.805
1.792
1.779
1.765
1.756
1.743
1.733
1.676
1.635
1.593
1.573
1.567
1.547
1.529
1.498
1.457
1.452
1.297
1.286
1.276
1.261
1.216
1.210
1.205
H3CO
Ph
N
O
(-)-FL-3-142
1.02
1
1.02
2.25
2.03
1.02
1.5
1.23
1.67
3.01
0.683
1.29
2.1
1.42
1.42
1.13
5.53
1.09
ppm 7
6
5
4
3
2

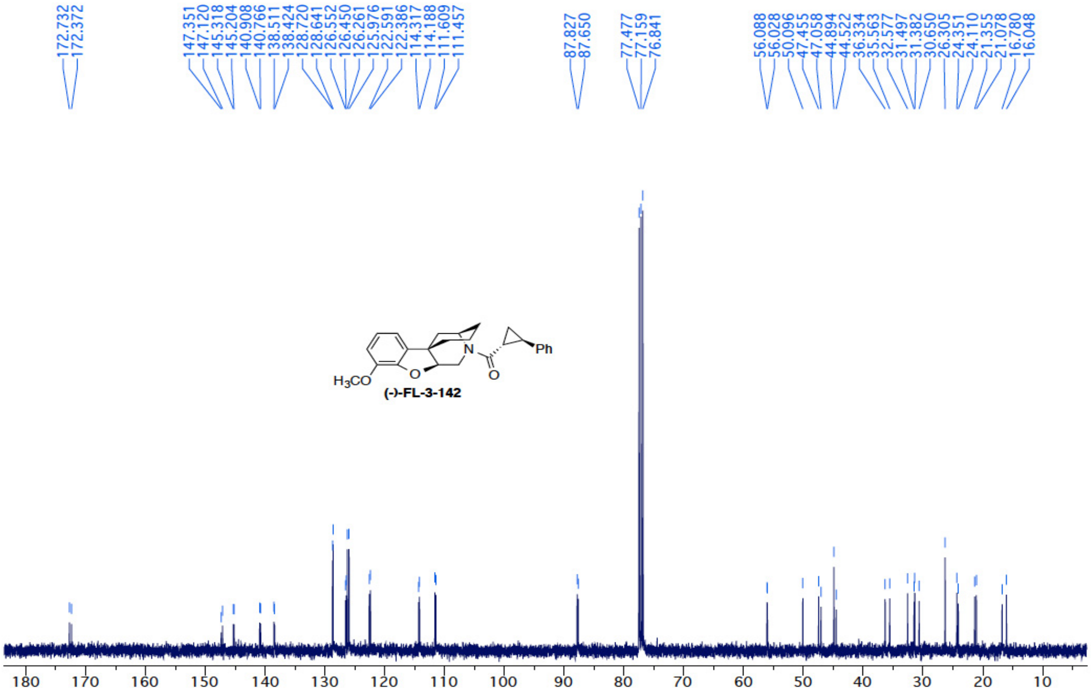

172.732
172.372
147.351
147.120
145.318
145.204
140.908
140.766
138.511
138.424
128.720
128.641
126.552
126.450
126.261
125.976
122.591
122.386
114.317
114.188
111.609
111.457
87.827
87.650
77.477
77.159
76.841
56.088
56.028
50.096
47.455
47.058
44.894
44.522
36.334
35.563
32.577
31.497
31.382
30.650
26.305
24.351
24.110
21.355
21.078
16.780
16.048
H3CO
Ph
N
O
(-)-FL-3-142
180
170
160
150
140
130
120
110
100
90
80
70
60
50
40
30
20
10

Compound (+)-**12**

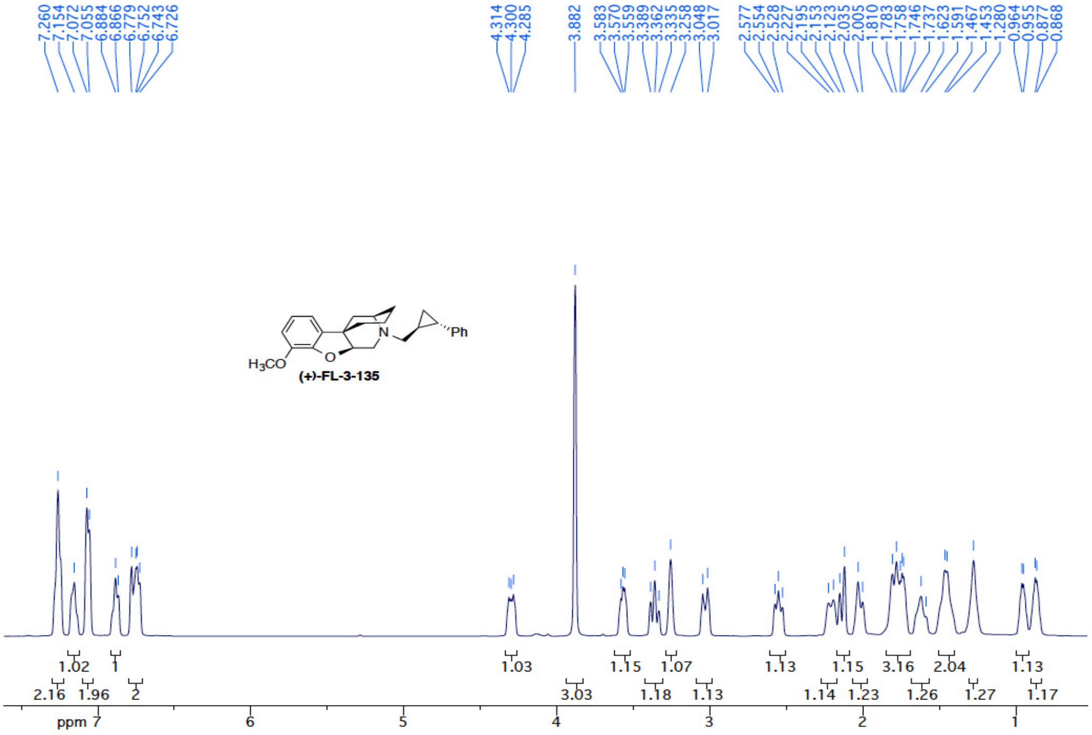
7.260
7.154
7.072
7.055
6.884
6.866
6.779
6.752
6.743
6.726
4.314
4.300
4.285
3.882
3.583
3.570
3.559
3.389
3.362
3.335
3.258
3.048
3.017
2.577
2.554
2.528
2.227
2.195
2.153
2.123
2.035
2.005
1.810
1.783
1.758
1.746
1.737
1.623
1.591
1.467
1.453
1.280
0.964
0.955
0.877
0.868
H3CO
N
O
Ph
(+)-FL-3-135
1.02
1
2.16
1.96
2
1.03
3.03
1.15
1.07
1.18
1.13
1.13
1.15
3.16
2.04
1.13
1.14
1.23
1.26
1.27
1.17
ppm 7
6
5
4
3
2
1

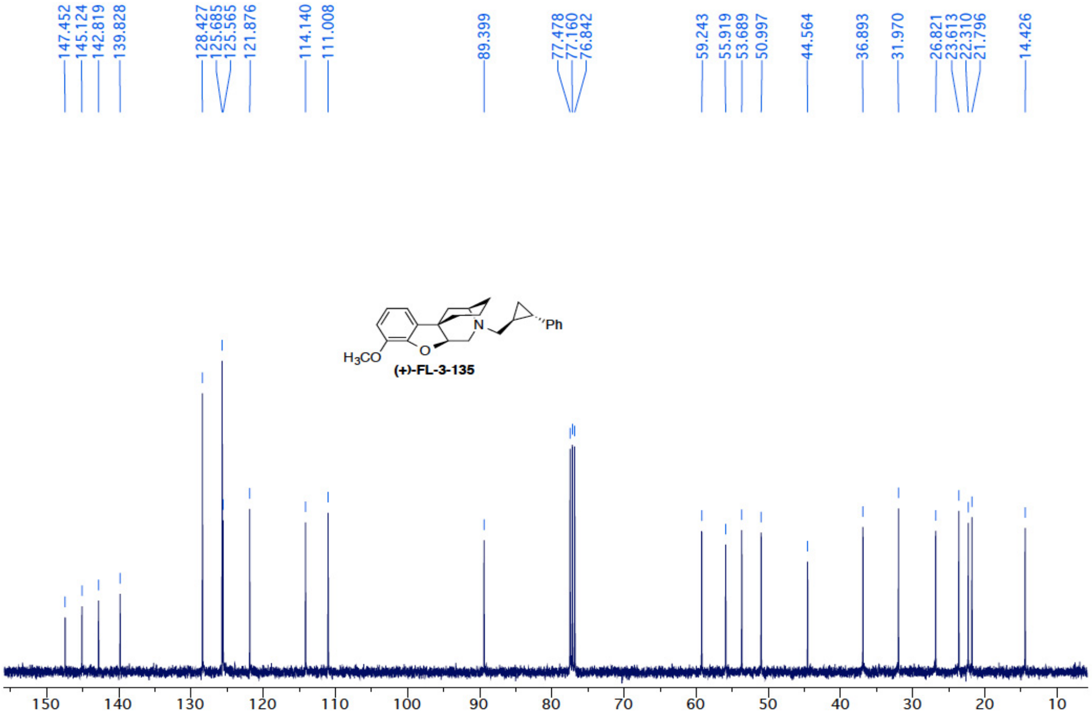
147.452
145.124
142.819
139.828
128.427
125.685
125.565
121.876
114.140
111.008
89.399
77.478
77.160
76.842
59.243
55.919
53.689
50.997
44.564
36.893
31.970
26.821
23.613
22.310
21.796
14.426
H3CO
N
O
Ph
(+)-FL-3-135
150
140
130
120
110
100
90
80
70
60
50
40
30
20
10

Compound (-)-**12**

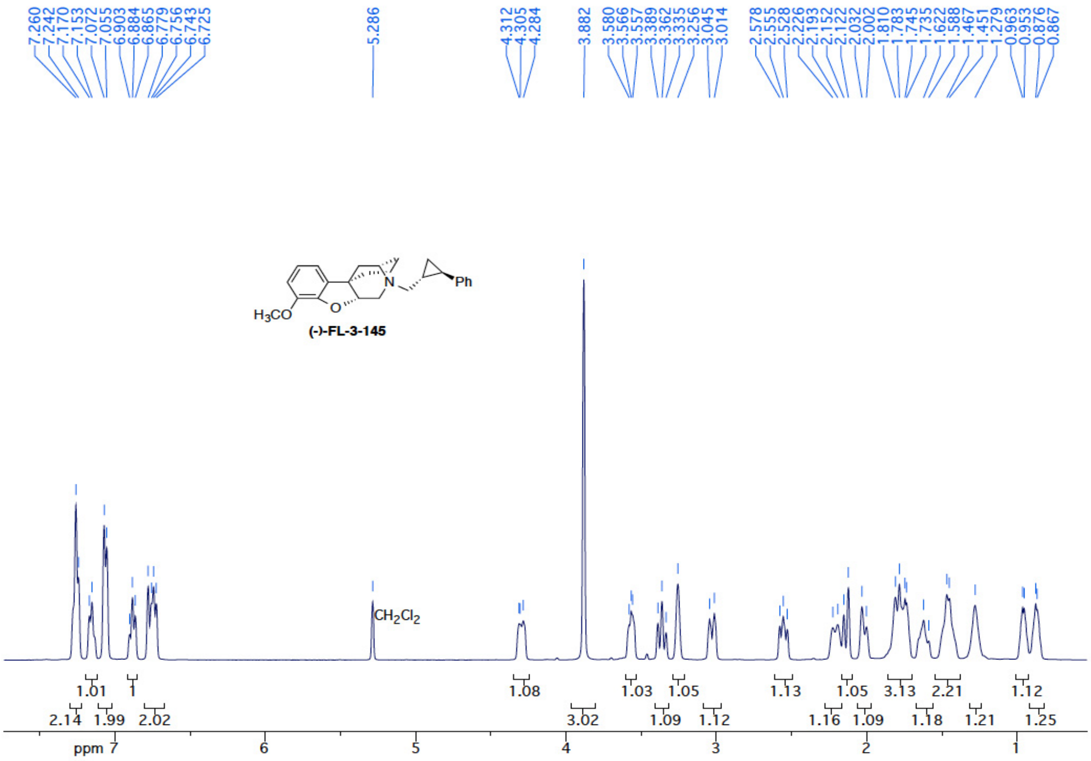
7.260 7.242 7.170 7.153 7.072 7.055 6.903 6.884 6.865 6.779 6.756 6.743 6.725
5.286
4.312 4.305 4.284
3.882 3.580 3.566 3.557 3.389 3.362 3.335 3.256 3.045 3.014
2.578 2.555 2.528 2.226 2.193 2.152 2.122 2.032 2.002 1.810 1.783 1.745 1.735 1.622 1.588 1.467 1.451 1.279 0.963 0.953 0.876 0.867
H_3CO
N
O
Ph
(-)-FL-3-145
CH_2Cl_2
1.01 1 1.08 1.03 1.05 1.13 1.05 3.13 2.21 1.12
2.14 1.99 2.02 3.02 1.09 1.12 1.16 1.09 1.18 1.21 1.25
ppm 7 6 5 4 3 2 1

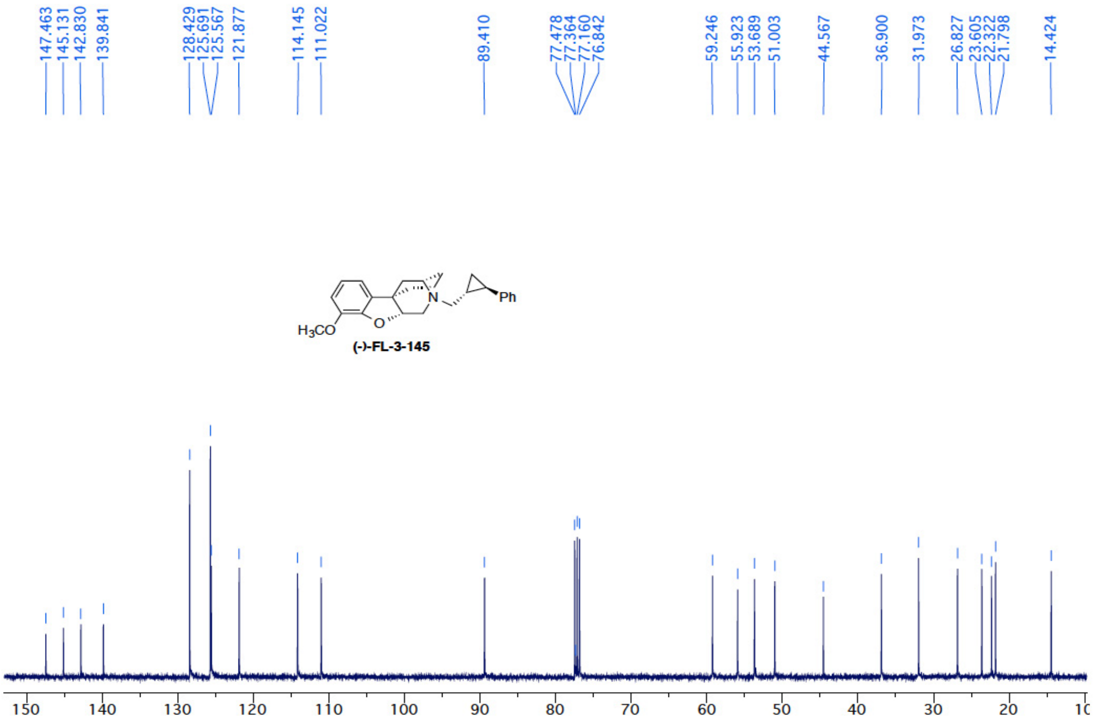
147.463 145.131 142.830 139.841
128.429 125.691 125.567 121.877
114.145 111.022
89.410
77.478 77.364 77.160 76.842
59.246 55.923 53.689 51.003
44.567
36.900 31.973
26.827 23.605 22.322 21.798
14.424
H_3CO
N
O
Ph
(-)-FL-3-145
150 140 130 120 110 100 90 80 70 60 50 40 30 20 10

Compound (+)-**13**

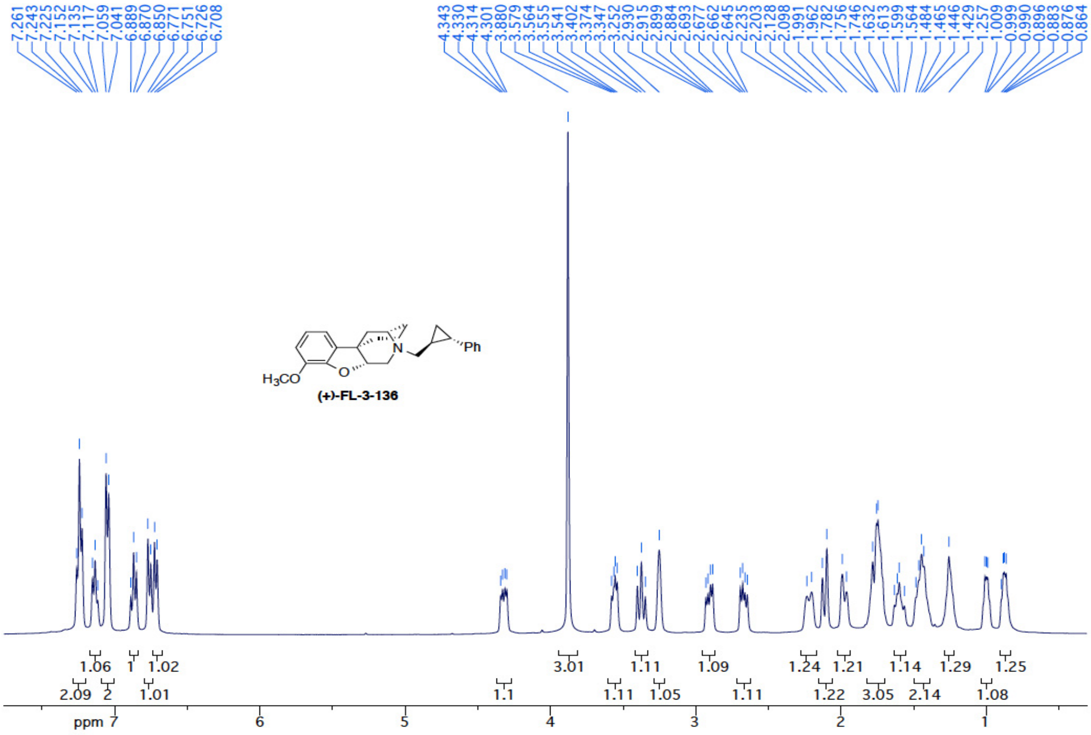

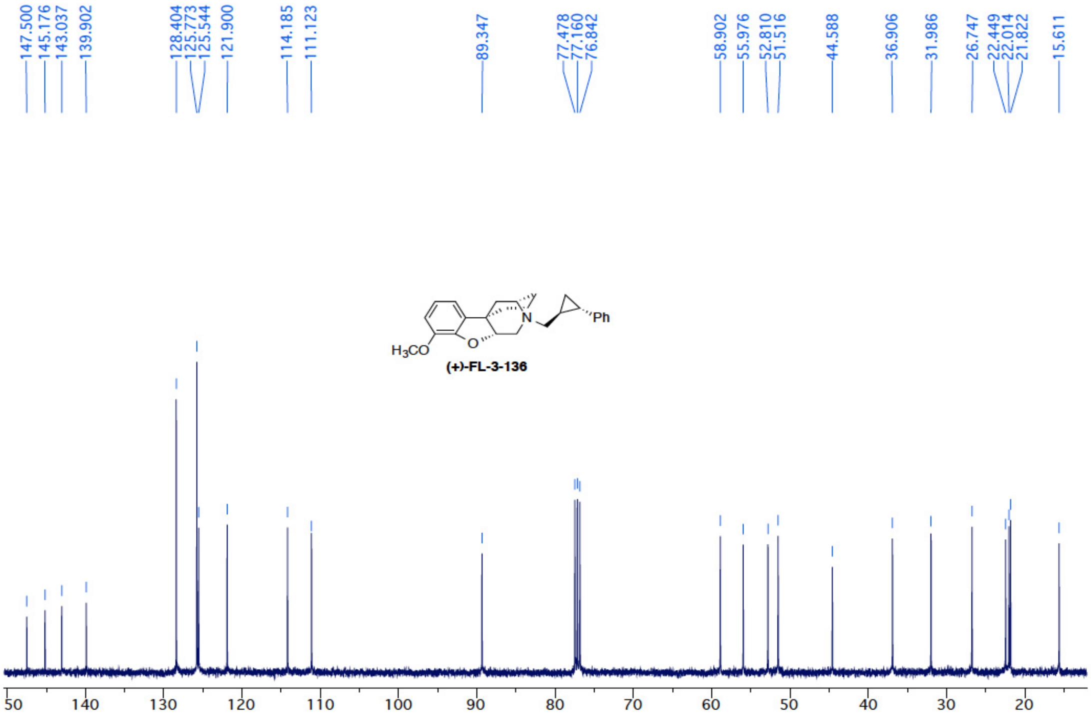

Compound (-)-**13**

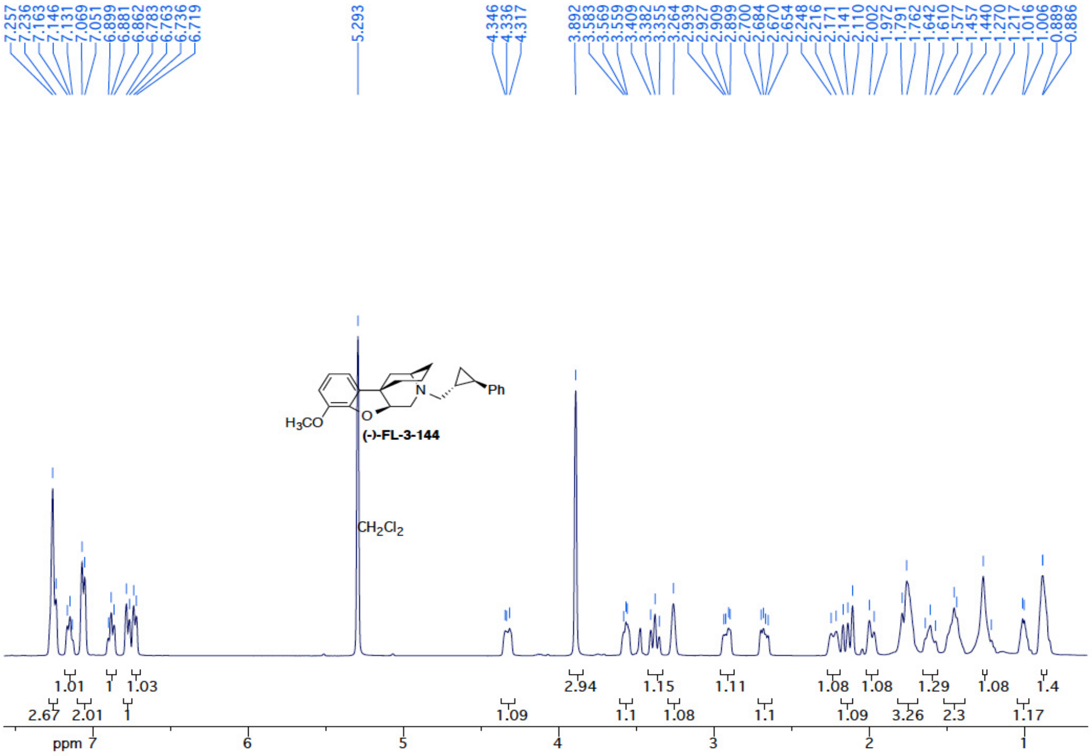
7.257
7.236
7.163
7.146
7.131
7.069
7.051
6.899
6.881
6.862
6.783
6.763
6.736
6.719
5.293
4.346
4.336
4.317
3.892
3.583
3.569
3.559
3.409
3.382
3.355
3.264
2.939
2.927
2.909
2.899
2.700
2.684
2.670
2.654
2.248
2.216
2.171
2.141
2.110
2.002
1.972
1.791
1.762
1.642
1.610
1.577
1.457
1.440
1.270
1.217
1.016
1.006
0.889
0.886
H3CO
O
N
Ph
(-)-FL-3-144
CH2Cl2
1.01
1
1.03
2.67
2.01
1
1.09
2.94
1.1
1.15
1.08
1.11
1.1
1.08
1.09
1.08
3.26
1.29
2.3
1.08
1.17
1.4
ppm 7
6
5
4
3
2
1

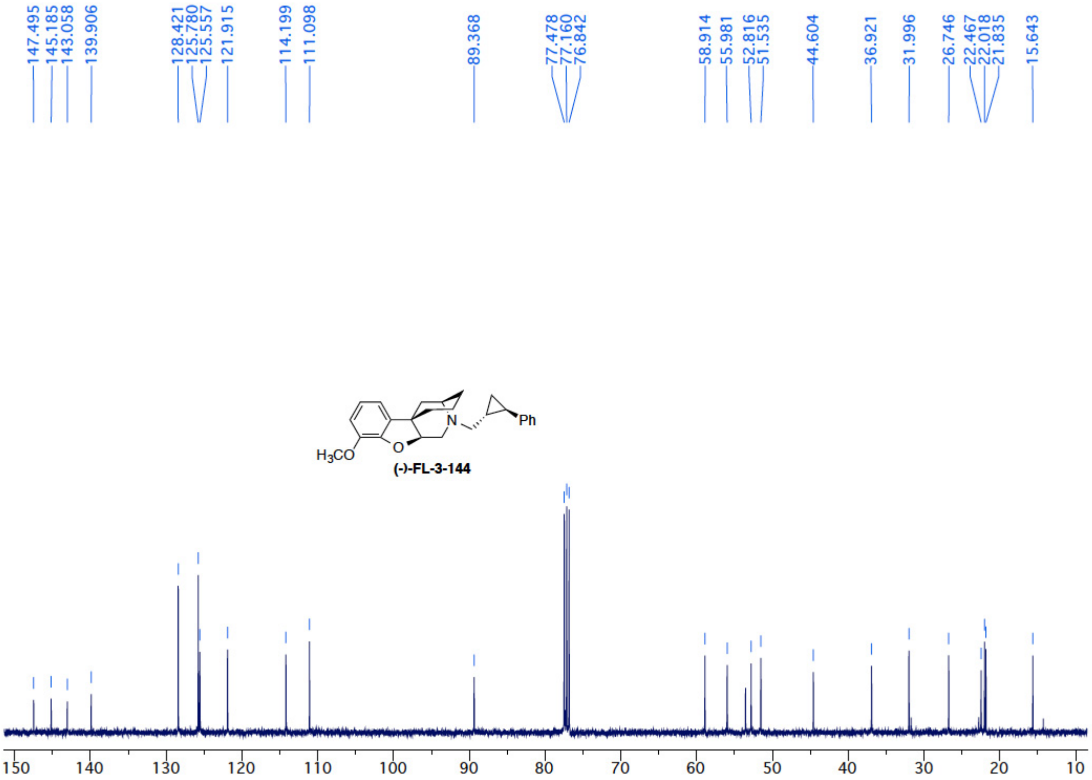
147.495
145.185
143.058
139.906
128.421
125.780
125.557
121.915
114.199
111.098
89.368
77.478
77.160
76.842
58.914
55.981
52.816
51.535
44.604
36.921
31.996
26.746
22.467
22.018
21.835
15.643
H3CO
O
N
Ph
(-)-FL-3-144
150
140
130
120
110
100
90
80
70
60
50
40
30
20
10

Compound (+)-**16**

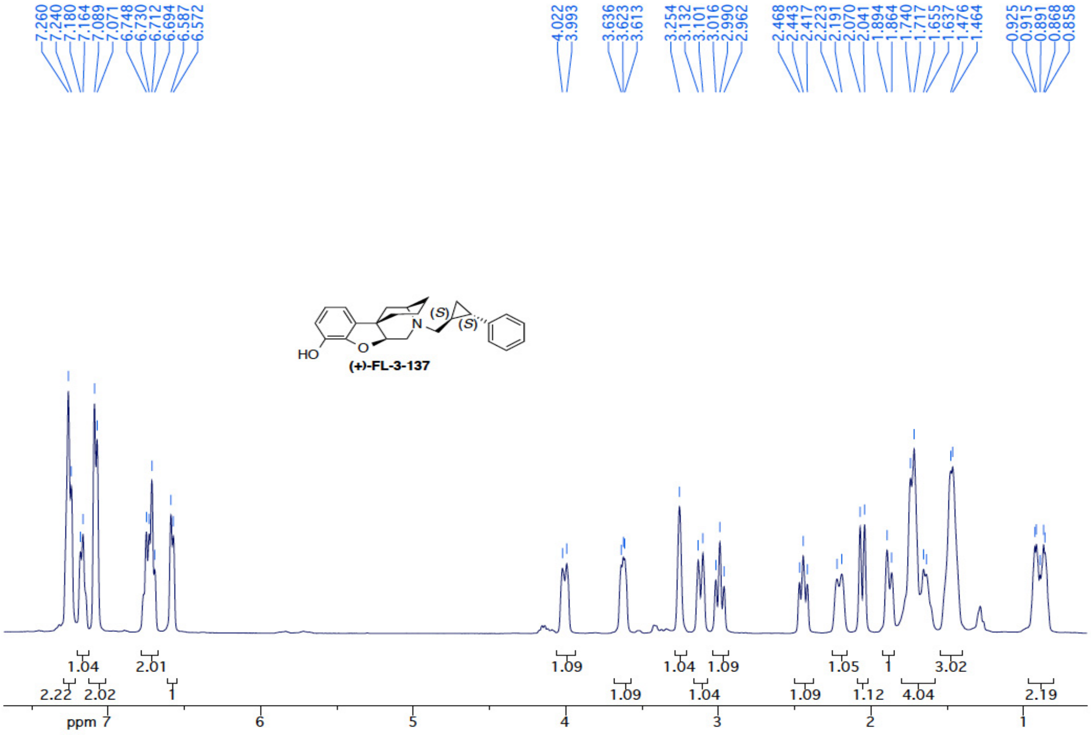
7.260
7.240
7.180
7.164
7.089
7.071
6.748
6.730
6.712
6.694
6.587
6.572
4.022
3.993
3.636
3.623
3.613
3.254
3.132
3.101
3.016
2.990
2.962
2.468
2.443
2.417
2.223
2.191
2.070
2.041
1.894
1.864
1.740
1.717
1.655
1.637
1.476
1.464
0.925
0.915
0.891
0.868
0.858
HO
O
N
(S)
(S)
(+)-FL-3-137
1.04
2.01
1.09
1.04
1.09
1.05
1
3.02
2.22
2.02
1
1.09
1.04
1.09
1.12
4.04
2.19
ppm 7
6
5
4
3
2
1

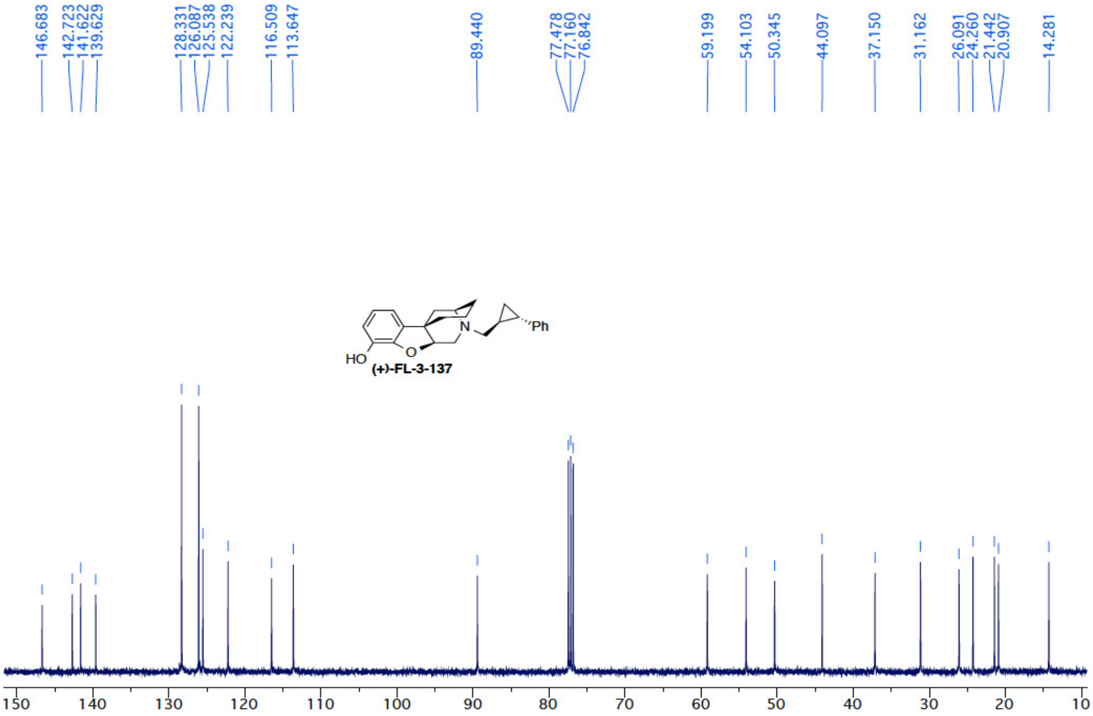
146.683
142.723
141.622
139.629
128.331
126.087
125.538
122.239
116.509
113.647
89.440
77.478
77.160
76.842
59.199
54.103
50.345
44.097
37.150
31.162
26.091
24.260
21.442
20.907
14.281
HO
O
N
Ph
(+)-FL-3-137
150
140
130
120
110
100
90
80
70
60
50
40
30
20
10

Compound (-)-**16**

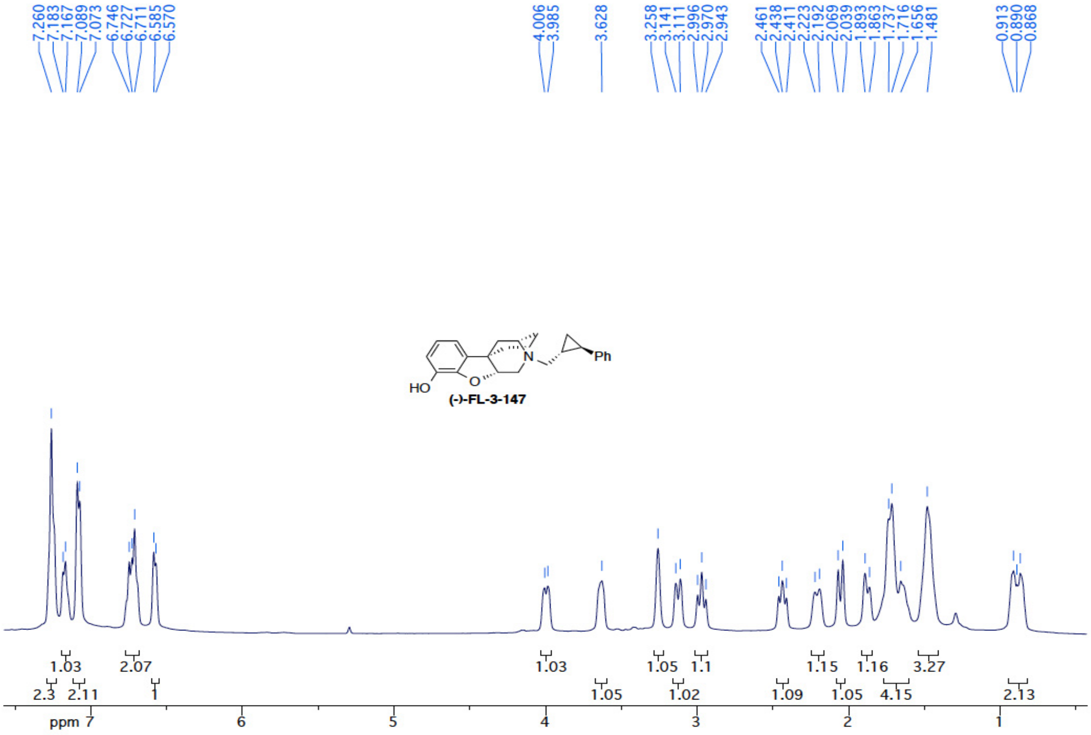

7.260 7.183 7.167 7.089 7.073 6.746 6.727 6.711 6.585 6.570
4.006 3.985
3.628
3.258 3.141 3.111 2.996 2.970 2.943
2.461 2.438 2.411 2.223 2.192 2.069 2.039 1.893 1.863 1.737 1.716 1.656 1.481
0.913 0.890 0.868
HO
Ph
N
O
(-)-FL-3-147
ppm

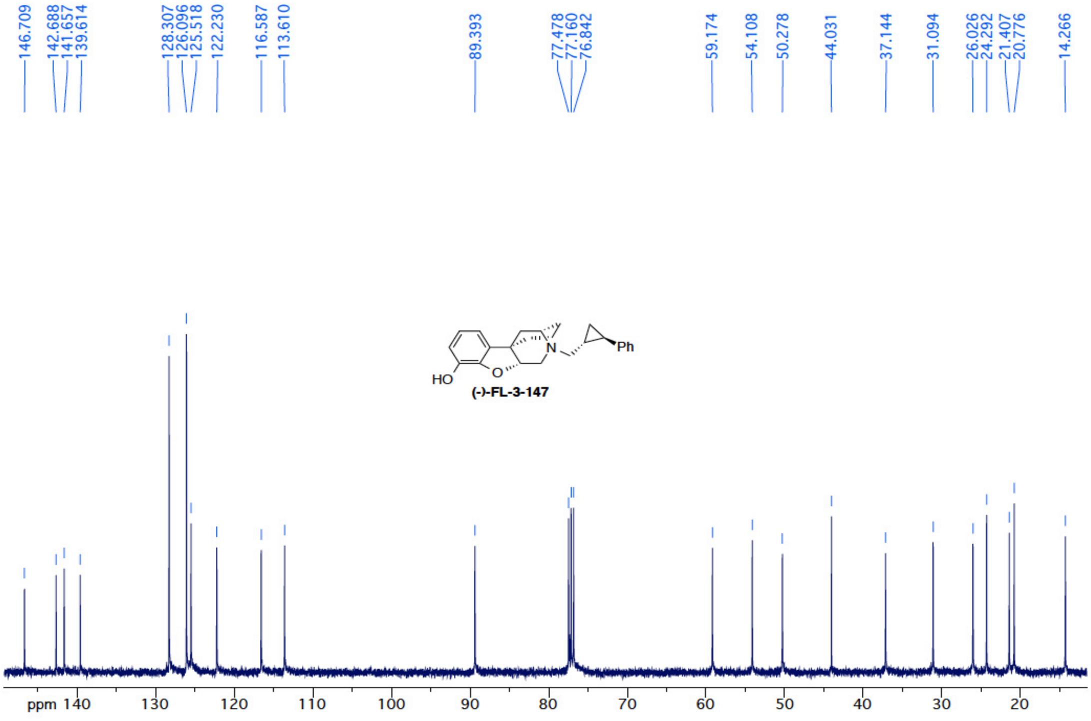

146.709 142.688 141.657 139.614
128.307 126.096 125.518 122.230
116.587 113.610
89.393
77.478 77.160 76.842
59.174 54.108 50.278 44.031 37.144 31.094 26.026 24.292 21.407 20.776 14.266
HO
Ph
N
O
(-)-FL-3-147
ppm

Compound (+)-**17**

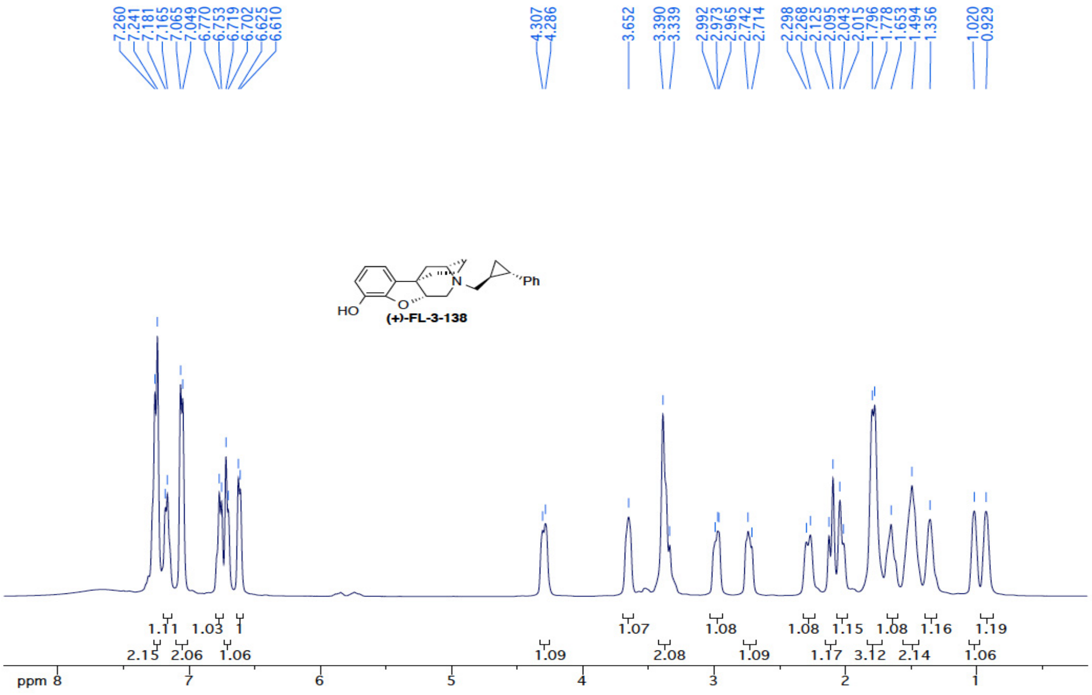

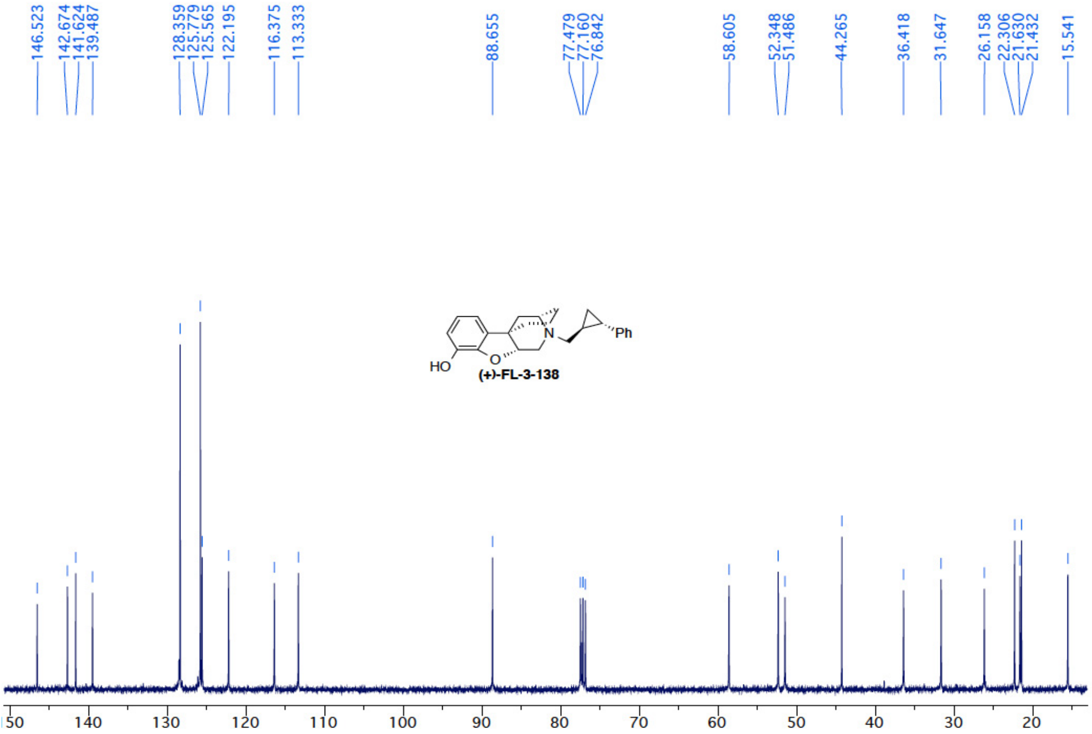

Compound (-)-**17**

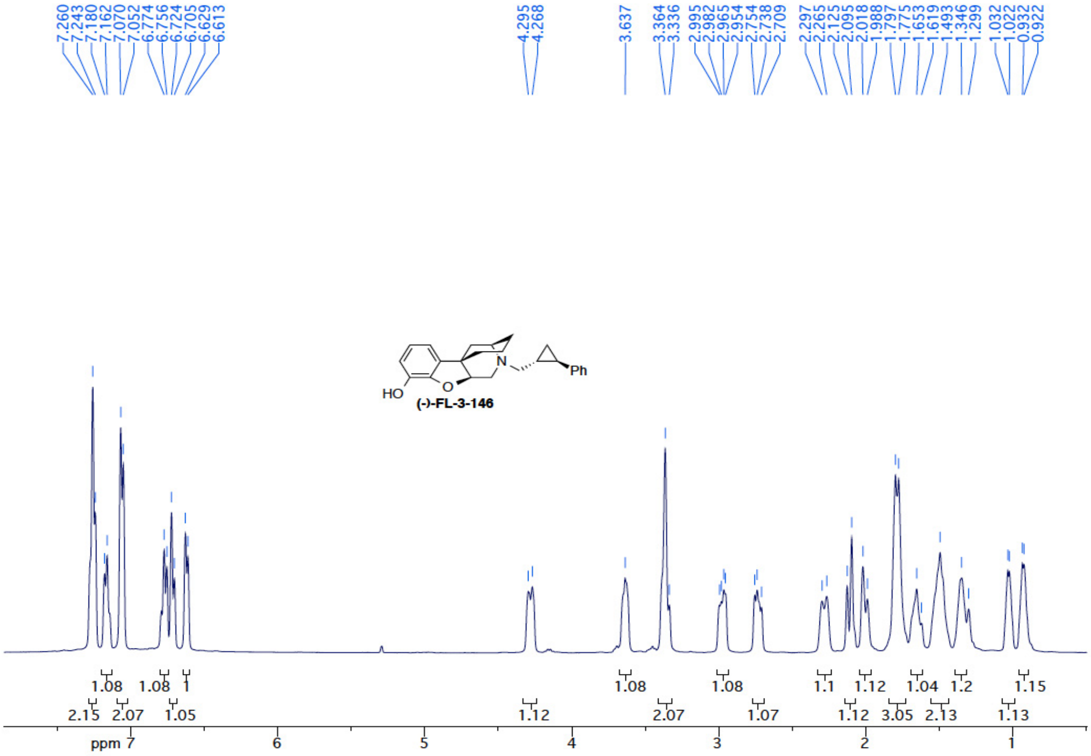
7.260
7.243
7.180
7.162
7.070
7.052
6.774
6.756
6.724
6.705
6.629
6.613
4.295
4.268
3.637
3.364
3.336
2.995
2.982
2.965
2.954
2.754
2.738
2.709
2.297
2.265
2.125
2.095
2.018
1.988
1.797
1.775
1.653
1.619
1.493
1.346
1.299
1.032
1.022
0.932
0.922
HO
O
N
Ph
(-)-FL-3-146
1.08
1.08
1
2.15
2.07
1.05
1.12
1.08
2.07
1.08
1.07
1.1
1.12
1.12
3.05
1.04
2.13
1.2
1.15
1.13
ppm 7
6
5
4
3
2
1

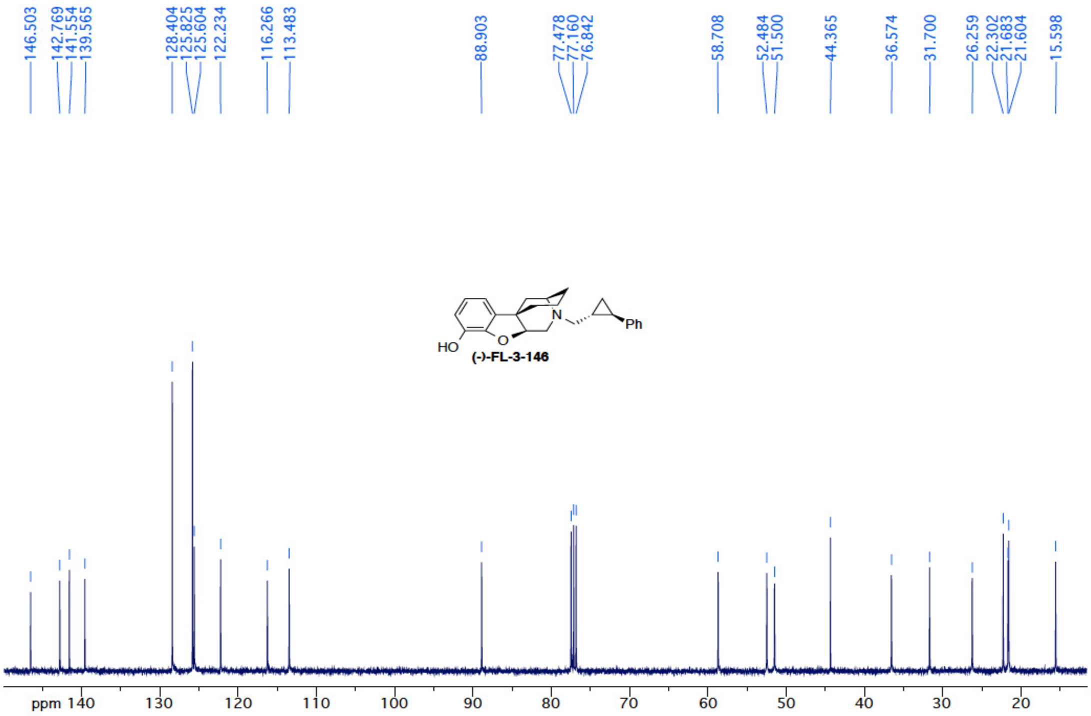
146.503
142.769
141.554
139.565
128.404
125.825
125.604
122.234
116.266
113.483
88.903
77.478
77.160
76.842
58.708
52.484
51.500
44.365
36.574
31.700
26.259
22.302
21.683
21.604
15.598
HO
O
N
Ph
(-)-FL-3-146
ppm 140
130
120
110
100
90
80
70
60
50
40
30
20

Table S1. Crystal data and structure refinement for **(-)-6**

Identification code	knih103	
Empirical formula	$C_{24}H_{31}NO_6$	
Formula weight	429.50	
Temperature	293(2) K	
Wavelength	1.54178 Å	
Crystal system	Orthorhombic	
Space group	$P2_12_12_1$	
Unit cell dimensions	a = 6.7324(6) Å	〈= 90°.
	b = 9.4370(6) Å	®= 90°.
	c = 34.612(4) Å	© = 90°.
Volume	2199.0(3) $Å^3$	
Z	4	
Density (20°C)	1.297 Mg/m^3	
Absorption coefficient	0.759 mm^{-1}	
F(000)	920	
Crystal size	0.211 x 0.102 x 0.040 mm^3	
Theta range for data collection	2.553 to 74.508°.	
Index ranges	-6<=h<=8, -11<=k<=10, -40<=l<=42	
Reflections collected	16601	
Independent reflections	4436 [R_{int} = 0.0558]	
Completeness to theta = 25.000°	98.8 %	
Absorption correction	Semi-empirical from equivalents	
Max. and min. transmission	0.7538 and 0.5283	
Refinement method	Full-matrix least-squares on F^2	
Data / restraints / parameters	4436 / 3 / 291	
Goodness-of-fit on F^2	1.091	
Final R indices [I>2sigma(I)]	R_1 = 0.0466, wR_2 = 0.1120	
R indices (all data)	R_1 = 0.0480, wR_2 = 0.1137	
Absolute structure parameter	0.10(8)	
Largest diff. peak and hole	0.298 and -0.388 e.$Å^{-3}$	

Table S2. Atomic coordinates (x 10^4) and equivalent isotropic displacement parameters (Å^{2}x 10^3) for **(-)-6**. U(eq) is defined as one third of the trace of the orthogonalized U^{ij} tensor.

	x	y	z	U(eq)
C(1)	3200(3)	4745(2)	3486(1)	35(1)
N(2)	3254(3)	5165(2)	3067(1)	35(1)
C(3)	3074(3)	6735(2)	2972(1)	38(1)
C(3A)	4569(3)	7609(2)	3206(1)	37(1)
C(4)	966(4)	7266(3)	3039(1)	48(1)
C(5)	362(3)	7318(3)	3464(1)	49(1)
C(6)	2024(3)	7758(2)	3739(1)	38(1)
C(6A)	4167(3)	7304(2)	3631(1)	29(1)
C(6B)	5542(3)	7847(2)	3946(1)	32(1)
C(7)	6423(3)	9149(2)	4017(1)	41(1)
C(8)	7506(4)	9301(3)	4354(1)	48(1)
C(9)	7648(3)	8205(3)	4623(1)	46(1)
C(10)	6713(3)	6909(2)	4559(1)	36(1)
O(10)	6645(3)	5783(2)	4807(1)	46(1)
C(10A)	7629(5)	5932(4)	5167(1)	70(1)
C(11)	5708(3)	6754(2)	4211(1)	31(1)
O(11)	4768(2)	5518(2)	4097(1)	35(1)
C(11A)	4616(3)	5733(2)	3685(1)	29(1)
C(1')	-1562(3)	3347(2)	2951(1)	31(1)
O(1')	200(2)	3430(2)	2839(1)	39(1)
O(1'')	-2911(3)	4195(2)	2871(1)	47(1)
C(2')	-2045(3)	2040(2)	3210(1)	37(1)
O(2')	-4099(3)	1862(2)	3280(1)	49(1)
C(3')	-991(3)	2215(2)	3594(1)	34(1)
C(4')	886(3)	1654(2)	3659(1)	39(1)
C(5')	1885(4)	1908(2)	4002(1)	44(1)
C(6')	1054(4)	2736(3)	4291(1)	45(1)
C(7')	-850(4)	3260(3)	4230(1)	47(1)
C(8')	-1859(4)	3002(2)	3889(1)	42(1)
C(9')	2176(6)	3086(4)	4655(1)	71(1)
O(1S)	3135(3)	1293(3)	2704(1)	62(1)

Table S3. Bond lengths [Å] and angles [°] for **(-)-6**
.

C(1)-C(11A)	1.501(3)	C(1)-N(2)	1.507(3)
C(1)-H(1A)	0.9700	C(1)-H(1B)	0.9700
N(2)-C(3)	1.522(3)	N(2)-H(2A)	0.8900
N(2)-H(2B)	0.8900	C(3)-C(4)	1.523(3)
C(3)-C(3A)	1.533(3)	C(3)-H(3)	0.9800
C(3A)-C(6A)	1.523(3)	C(3A)-H(3A)	0.9700
C(3A)-H(3B)	0.9700	C(4)-C(5)	1.526(4)
C(4)-H(4A)	0.9700	C(4)-H(4B)	0.9700
C(5)-C(6)	1.528(3)	C(5)-H(5A)	0.9700
C(5)-H(5B)	0.9700	C(6)-C(6A)	1.551(3)
C(6)-H(6A)	0.9700	C(6)-H(6B)	0.9700
C(6A)-C(6B)	1.518(3)	C(6A)-C(11A)	1.525(3)
C(6B)-C(7)	1.386(3)	C(6B)-C(11)	1.386(3)
C(7)-C(8)	1.385(4)	C(7)-H(7)	0.9300
C(8)-C(9)	1.395(4)	C(8)-H(8)	0.9300
C(9)-C(10)	1.393(3)	C(9)-H(9)	0.9300
C(10)-O(10)	1.367(3)	C(10)-C(11)	1.390(3)
O(10)-C(10A)	1.419(3)	C(10A)-H(10A)	0.9600
C(10A)-H(10B)	0.9600	C(10A)-H(10C)	0.9600
C(11)-O(11)	1.384(2)	O(11)-C(11A)	1.443(2)
C(11A)-H(11A)	0.9800	C(1')-O(1'')	1.242(3)
C(1')-O(1')	1.251(3)	C(1')-C(2')	1.558(3)
C(2')-O(2')	1.414(3)	C(2')-C(3')	1.517(3)
C(2')-H(2')	0.9800	O(2')-H(2C)	0.85(4)
C(3')-C(4')	1.388(3)	C(3')-C(8')	1.392(3)
C(4')-C(5')	1.386(3)	C(4')-H(4')	0.9300
C(5')-C(6')	1.387(4)	C(5')-H(5')	0.9300
C(6')-C(7')	1.390(4)	C(6')-C(9')	1.508(4)
C(7')-C(8')	1.383(4)	C(7')-H(7')	0.9300
C(8')-H(8')	0.9300	C(9')-H(9D)	0.9600
C(9')-H(9E)	0.9600	C(9')-H(9F)	0.9600
O(1S)-H(1S)	0.8199(14)	O(1S)-H(2S)	0.8201(14)
C(11A)-C(1)-N(2)	105.32(17)	C(11A)-C(1)-H(1A)	110.7
N(2)-C(1)-H(1A)	110.7	C(11A)-C(1)-H(1B)	110.7
N(2)-C(1)-H(1B)	110.7	H(1A)-C(1)-H(1B)	108.8
C(1)-N(2)-C(3)	117.49(16)	C(1)-N(2)-H(2A)	107.9
C(3)-N(2)-H(2A)	107.9	C(1)-N(2)-H(2B)	107.9
C(3)-N(2)-H(2B)	107.9	H(2A)-N(2)-H(2B)	107.2
N(2)-C(3)-C(4)	111.2(2)	N(2)-C(3)-C(3A)	110.98(17)
C(4)-C(3)-C(3A)	110.75(19)	N(2)-C(3)-H(3)	107.9
C(4)-C(3)-H(3)	107.9	C(3A)-C(3)-H(3)	107.9
C(6A)-C(3A)-C(3)	106.95(17)	C(6A)-C(3A)-H(3A)	110.3
C(3)-C(3A)-H(3A)	110.3	C(6A)-C(3A)-H(3B)	110.3

Table S3. (continued).

C(3)-C(3A)-H(3B)	110.3	H(3A)-C(3A)-H(3B)	108.6
C(3)-C(4)-C(5)	113.90(19)	C(3)-C(4)-H(4A)	108.8
C(5)-C(4)-H(4A)	108.8	C(3)-C(4)-H(4B)	108.8
C(5)-C(4)-H(4B)	108.8	H(4A)-C(4)-H(4B)	107.7
C(4)-C(5)-C(6)	114.5(2)	C(4)-C(5)-H(5A)	108.6
C(6)-C(5)-H(5A)	108.6	C(4)-C(5)-H(5B)	108.6
C(6)-C(5)-H(5B)	108.6	H(5A)-C(5)-H(5B)	107.6
C(5)-C(6)-C(6A)	117.08(18)	C(5)-C(6)-H(6A)	108.0
C(6A)-C(6)-H(6A)	108.0	C(5)-C(6)-H(6B)	108.0
C(6A)-C(6)-H(6B)	108.0	H(6A)-C(6)-H(6B)	107.3
C(6B)-C(6A)-C(3A)	121.34(17)	C(6B)-C(6A)-C(11A)	96.83(15)
C(3A)-C(6A)-C(11A)	105.57(17)	C(6B)-C(6A)-C(6)	107.49(16)
C(3A)-C(6A)-C(6)	110.25(17)	C(11A)-C(6A)-C(6)	115.05(17)
C(7)-C(6B)-C(11)	120.5(2)	C(7)-C(6B)-C(6A)	133.5(2)
C(11)-C(6B)-C(6A)	105.86(17)	C(8)-C(7)-C(6B)	117.9(2)
C(8)-C(7)-H(7)	121.1	C(6B)-C(7)-H(7)	121.1
C(7)-C(8)-C(9)	121.5(2)	C(7)-C(8)-H(8)	119.3
C(9)-C(8)-H(8)	119.3	C(10)-C(9)-C(8)	120.9(2)
C(10)-C(9)-H(9)	119.5	C(8)-C(9)-H(9)	119.5
O(10)-C(10)-C(11)	116.51(18)	O(10)-C(10)-C(9)	126.7(2)
C(11)-C(10)-C(9)	116.8(2)	C(10)-O(10)-C(10A)	117.3(2)
O(10)-C(10A)-H(10A)	109.5	O(10)-C(10A)-H(10B)	109.5
H(10A)-C(10A)-H(10B)	109.5	O(10)-C(10A)-H(10C)	109.5
H(10A)-C(10A)-H(10C)	109.5	H(10B)-C(10A)-H(10C)	
	109.5		
O(11)-C(11)-C(6B)	113.68(17)	O(11)-C(11)-C(10)	123.91(18)
C(6B)-C(11)-C(10)	122.40(19)	C(11)-O(11)-C(11A)	101.25(15)
O(11)-C(11A)-C(1)	114.29(16)	O(11)-C(11A)-C(6A)	105.82(15)
C(1)-C(11A)-C(6A)	114.90(17)	O(11)-C(11A)-H(11A)	107.1
C(1)-C(11A)-H(11A)	107.1	C(6A)-C(11A)-H(11A)	107.1
O(1")-C(1')-O(1')	125.7(2)	O(1")-C(1')-C(2')	119.09(18)
O(1')-C(1')-C(2')	115.23(19)	O(2')-C(2')-C(3')	108.64(19)
O(2')-C(2')-C(1')	113.41(18)	C(3')-C(2')-C(1')	108.72(16)
O(2')-C(2')-H(2')	108.7	C(3')-C(2')-H(2')	108.7
C(1')-C(2')-H(2')	108.7	C(2')-O(2')-H(2C)	107(3)
C(4')-C(3')-C(8')	117.9(2)	C(4')-C(3')-C(2')	121.7(2)
C(8')-C(3')-C(2')	120.4(2)	C(5')-C(4')-C(3')	120.9(2)
C(5')-C(4')-H(4')	119.5	C(3')-C(4')-H(4')	119.5
C(4')-C(5')-C(6')	121.3(2)	C(4')-C(5')-H(5')	119.4
C(6')-C(5')-H(5')	119.4	C(5')-C(6')-C(7')	117.7(2)
C(5')-C(6')-C(9')	121.7(3)	C(7')-C(6')-C(9')	120.6(3)
C(8')-C(7')-C(6')	121.2(2)	C(8')-C(7')-H(7')	119.4
C(6')-C(7')-H(7')	119.4	C(7')-C(8')-C(3')	120.9(2)
C(7')-C(8')-H(8')	119.5	C(3')-C(8')-H(8')	119.5

Table S3. (continued).

C(6')-C(9')-H(9D)	109.5	C(6')-C(9')-H(9E)	109.5
H(9D)-C(9')-H(9E)	109.5	C(6')-C(9')-H(9F)	109.5
H(9D)-C(9')-H(9F)	109.5	H(9E)-C(9')-H(9F)	109.5
H(1S)-O(1S)-H(2S)	104.5(2)		

Table S4. Anisotropic displacement parameters ($Å^2$x 10^3) for **(-)-6**. The anisotropic displacement factor exponent takes the form: $-2 \pi^2[h^2a^{*2}U^{11} + ... + 2 h k a^* b^* U^{12}]$

	U^{11}	U^{22}	U^{33}	U^{23}	U^{13}	U^{12}
C(1)	38(1)	32(1)	37(1)	-1(1)	-6(1)	-4(1)
N(2)	29(1)	43(1)	34(1)	-7(1)	-1(1)	-1(1)
C(3)	39(1)	45(1)	31(1)	5(1)	0(1)	-1(1)
C(3A)	39(1)	37(1)	36(1)	7(1)	4(1)	-4(1)
C(4)	43(1)	56(1)	45(1)	4(1)	-12(1)	11(1)
C(5)	31(1)	64(2)	52(1)	-3(1)	-2(1)	13(1)
C(6)	36(1)	38(1)	42(1)	-3(1)	2(1)	7(1)
C(6A)	29(1)	26(1)	33(1)	2(1)	1(1)	-1(1)
C(6B)	30(1)	29(1)	37(1)	-3(1)	2(1)	-1(1)
C(7)	39(1)	31(1)	52(1)	-3(1)	6(1)	-7(1)
C(8)	41(1)	41(1)	63(2)	-13(1)	1(1)	-14(1)
C(9)	38(1)	51(1)	49(1)	-15(1)	-6(1)	-6(1)
C(10)	32(1)	41(1)	35(1)	-5(1)	-2(1)	1(1)
O(10)	53(1)	50(1)	36(1)	1(1)	-10(1)	0(1)
C(10A)	74(2)	82(2)	53(2)	10(2)	-28(2)	-6(2)
C(11)	28(1)	31(1)	35(1)	-4(1)	-1(1)	-2(1)
O(11)	43(1)	28(1)	32(1)	2(1)	-7(1)	-4(1)
C(11A)	31(1)	26(1)	32(1)	0(1)	-1(1)	1(1)
C(1')	31(1)	34(1)	29(1)	-4(1)	-3(1)	-2(1)
O(1')	34(1)	46(1)	38(1)	-4(1)	0(1)	-3(1)
O(1'')	41(1)	49(1)	51(1)	15(1)	7(1)	10(1)
C(2')	40(1)	32(1)	39(1)	-1(1)	-4(1)	-3(1)
O(2')	42(1)	60(1)	45(1)	5(1)	-7(1)	-18(1)
C(3')	39(1)	27(1)	37(1)	4(1)	-3(1)	-1(1)
C(4')	40(1)	35(1)	41(1)	5(1)	0(1)	4(1)
C(5')	39(1)	44(1)	48(1)	13(1)	-6(1)	3(1)
C(6')	55(1)	40(1)	39(1)	9(1)	-10(1)	-7(1)
C(7')	61(2)	42(1)	39(1)	-3(1)	-4(1)	6(1)
C(8')	44(1)	39(1)	43(1)	0(1)	-4(1)	11(1)
C(9')	84(2)	77(2)	50(2)	8(1)	-25(2)	-15(2)
O(1S)	43(1)	83(1)	62(1)	-31(1)	-15(1)	14(1)

Table S5. Hydrogen coordinates (x 10^4) and isotropic displacement parameters (Å^{2}x 10^3) for **(-)-6**.

	x	y	z	U(eq)
H(1A)	1870	4849	3590	42
H(1B)	3619	3769	3518	42
H(2A)	2274	4712	2946	42
H(2B)	4390	4852	2967	42
H(3)	3385	6858	2697	46
H(3A)	5917	7341	3139	45
H(3B)	4401	8611	3153	45
H(4A)	50	6653	2901	57
H(4B)	845	8209	2930	57
H(5A)	-115	6388	3539	58
H(5B)	-733	7978	3492	58
H(6A)	1722	7374	3992	46
H(6B)	2001	8782	3762	46
H(7)	6291	9896	3843	49
H(8)	8153	10153	4403	58
H(9)	8376	8342	4849	55
H(10A)	9034	6015	5125	104
H(10B)	7367	5116	5325	104
H(10C)	7154	6767	5296	104
H(11A)	5936	5559	3576	35
H(2')	-1538	1186	3083	44
H(2C)	-4640(60)	1690(40)	3062(12)	74
H(4')	1480	1100	3469	46
H(5')	3138	1516	4039	52
H(7')	-1456	3795	4423	57
H(8')	-3136	3359	3857	50
H(9D)	2006	4072	4715	106
H(9E)	3562	2888	4619	106
H(9F)	1675	2522	4864	106
H(1S)	2710(50)	650(20)	2569(9)	94
H(2S)	2220(30)	1860(20)	2717(12)	94

Table S6. Torsion angles [°] for **(-)-6**.

C(11A)-C(1)-N(2)-C(3)	-47.6(2)	C(1)-N(2)-C(3)-C(4)	-72.5(2)
C(1)-N(2)-C(3)-C(3A)	51.3(3)	N(2)-C(3)-C(3A)-C(6A)	-57.3(2)
C(4)-C(3)-C(3A)-C(6A)	66.7(2)	N(2)-C(3)-C(4)-C(5)	69.3(3)
C(3A)-C(3)-C(4)-C(5)	-54.6(3)	C(3)-C(4)-C(5)-C(6)	36.8(3)
C(4)-C(5)-C(6)-C(6A)	-33.1(3)	C(3)-C(3A)-C(6A)-C(6B)	172.16(18)
C(3)-C(3A)-C(6A)-C(11A)	63.9(2)	C(3)-C(3A)-C(6A)-C(6)	-61.0(2)
C(5)-C(6)-C(6A)-C(6B)	-179.6(2)	C(5)-C(6)-C(6A)-C(3A)	46.1(3)
C(5)-C(6)-C(6A)-C(11A)	-73.1(3)	C(3A)-C(6A)-C(6B)-C(7)	46.1(3)
C(11A)-C(6A)-C(6B)-C(7)	159.0(2)	C(6)-C(6A)-C(6B)-C(7)	-82.0(3)
C(3A)-C(6A)-C(6B)-C(11)	-139.50(19)	C(11A)-C(6A)-C(6B)-C(11)	-26.59(19)
C(6)-C(6A)-C(6B)-C(11)	92.42(19)	C(11)-C(6B)-C(7)-C(8)	1.7(3)
C(6A)-C(6B)-C(7)-C(8)	175.4(2)	C(6B)-C(7)-C(8)-C(9)	-2.6(4)
C(7)-C(8)-C(9)-C(10)	0.5(4)	C(8)-C(9)-C(10)-O(10)	-176.2(2)
C(8)-C(9)-C(10)-C(11)	2.3(3)	C(11)-C(10)-O(10)-C(10A)	-178.8(2)
C(9)-C(10)-O(10)-C(10A)	-0.3(4)	C(7)-C(6B)-C(11)-O(11)	-179.36(19)
C(6A)-C(6B)-C(11)-O(11)	5.4(2)	C(7)-C(6B)-C(11)-C(10)	1.3(3)
C(6A)-C(6B)-C(11)-C(10)	-174.00(19)	O(10)-C(10)-C(11)-O(11)	-3.9(3)
C(9)-C(10)-C(11)-O(11)	177.47(19)	O(10)-C(10)-C(11)-C(6B)	175.42(19)
C(9)-C(10)-C(11)-C(6B)	-3.2(3)	C(6B)-C(11)-O(11)-C(11A)	20.1(2)
C(10)-C(11)-O(11)-C(11A)	-160.52(19)	C(11)-O(11)-C(11A)-C(1)	-165.13(17)
C(11)-O(11)-C(11A)-C(6A)	-37.68(19)	N(2)-C(1)-C(11A)-O(11)	179.24(16)
N(2)-C(1)-C(11A)-C(6A)	56.6(2)	C(6B)-C(6A)-C(11A)-O(11)	39.56(18)
C(3A)-C(6A)-C(11A)-O(11)	164.81(16)	C(6)-C(6A)-C(11A)-O(11)	-73.4(2)
C(6B)-C(6A)-C(11A)-C(1)	166.64(17)	C(3A)-C(6A)-C(11A)-C(1)	-68.1(2)
C(6)-C(6A)-C(11A)-C(1)	53.7(2)	O(1")-C(1')-C(2')-O(2')	8.8(3)
O(1')-C(1')-C(2')-O(2')	-170.61(19)	O(1")-C(1')-C(2')-C(3')	-112.2(2)
O(1')-C(1')-C(2')-C(3')	68.4(2)	O(2')-C(2')-C(3')-C(4')	143.6(2)
C(1')-C(2')-C(3')-C(4')	-92.5(2)	O(2')-C(2')-C(3')-C(8')	-39.1(3)
C(1')-C(2')-C(3')-C(8')	84.8(2)	C(8')-C(3')-C(4')-C(5')	-2.0(3)
C(2')-C(3')-C(4')-C(5')	175.4(2)	C(3')-C(4')-C(5')-C(6')	-0.4(3)
C(4')-C(5')-C(6')-C(7')	2.3(3)	C(4')-C(5')-C(6')-C(9')	-176.5(2)
C(5')-C(6')-C(7')-C(8')	-1.9(4)	C(9')-C(6')-C(7')-C(8')	176.9(3)
C(6')-C(7')-C(8')-C(3')	-0.5(4)	C(4')-C(3')-C(8')-C(7')	2.4(3)
C(2')-C(3')-C(8')-C(7')	-175.0(2)		

Table S7. Hydrogen bonds for **(-)-6** [Å and °].

D-H...A	d(D-H)	d(H...A)	d(D...A)	<(DHA)
C(1)-H(1B)...O(2')#1	0.97	2.51	3.349(3)	145.4
N(2)-H(2A)...O(1')	0.89	1.88	2.744(2)	161.8
N(2)-H(2B)...O(1")#1	0.89	1.95	2.822(2)	166.8
C(9)-H(9)...O(10)#2	0.93	2.63	3.469(3)	149.6
O(2')-H(2C)...O(1S)#3	0.85(4)	1.98(4)	2.781(3)	156(4)
O(1S)-H(1S)...O(1")#4	0.8199(14)	2.052(17)	2.810(3)	154(4)
O(1S)-H(2S)...O(1')	0.8201(14)	2.053(7)	2.862(3)	169(3)

Symmetry transformations used to generate equivalent atoms:
#1 x+1,y,z #2 x+1/2,-y+3/2,-z+1 #3 x-1,y,z
#4 -x,y-1/2,-z+1/2
